# Multicomponent synthesis and photophysical study of novel α,β-unsaturated carbonyl depsipeptides and peptoids

**DOI:** 10.3389/fchem.2023.1245941

**Published:** 2023-08-17

**Authors:** Ricelia González, Juliana Murillo-López, Walter Rabanal-León, Luis Prent-Peñaloza, Odette Concepción, Pedro Olivares, Yorley Duarte, Alexander F. de la Torre, Margarita Gutiérrez, Julio Caballero

**Affiliations:** ^1^ Doctorado en Ciencias Mención I + D de Productos Bioactivos, Laboratorio de Síntesis Orgánica y Actividad Biológica, Instituto de Química de Recursos Naturales, Universidad de Talca, Talca, Chile; ^2^ Departamento de Química Orgánica, Facultad de Ciencias Químicas, Universidad de Concepción, Concepción, Chile; ^3^ Departamento de Ciencias Químicas, Facultad de Ciencias Exactas, Universidad Andrés Bello, Viña del Mar, Chile; ^4^ Centro de Bioinformática y Biología Integrativa, Facultad de Ciencias de la Vida, Universidad Andrés Bello, Santiago, Chile; ^5^ Laboratorio de Síntesis Orgánica y Actividad Biológica, Instituto de Química de Recursos Naturales, Universidad de Talca, Talca, Chile; ^6^ Departamento de Bioinformática, Facultad de Ingeniería, Centro de Bioinformática, Simulación y Modelado (CBSM), Universidad de Talca, Talca, Chile

**Keywords:** multicomponent reactions, depsipeptides, peptoids, time-dependent density functional theory (TD-DFT), peptidomimetics

## Abstract

Multicomponent reactions were performed to develop novel α,β-unsaturated carbonyl depsipeptides and peptoids incorporating various chromophores such as cinnamic, coumarin, and quinolines. Thus, through the Passerini and Ugi multicomponent reactions (P-3CR and U-4CR), we obtained thirteen depsipeptides and peptoids in moderate to high yield following the established protocol and fundamentally varying the electron-rich carboxylic acid as reactants. UV/Vis spectroscopy was utilized to study the photophysical properties of the newly synthesized compounds. Differences between the carbonyl-substituted chromophores cause differences in electron delocalization that can be captured in the spectra. The near UV regions of all the compounds exhibited strong absorption bands. Compounds **P2**, **P5**, **U2**, **U5**, and **U7** displayed absorption bands in the range of 250–350 nm, absorbing radiation in this broad region of the electromagnetic spectrum. A photostability study for **U5** showed that its molecular structure does not change after exposure to UV radiation. Fluorescence analysis showed an incipient emission of **U5**, while **U6** showed blue fluorescence under UV radiation. The photophysical properties and electronic structure were also determined by TD-DFT theoretical study.

## 1 Introduction

**FIGURE 1:**
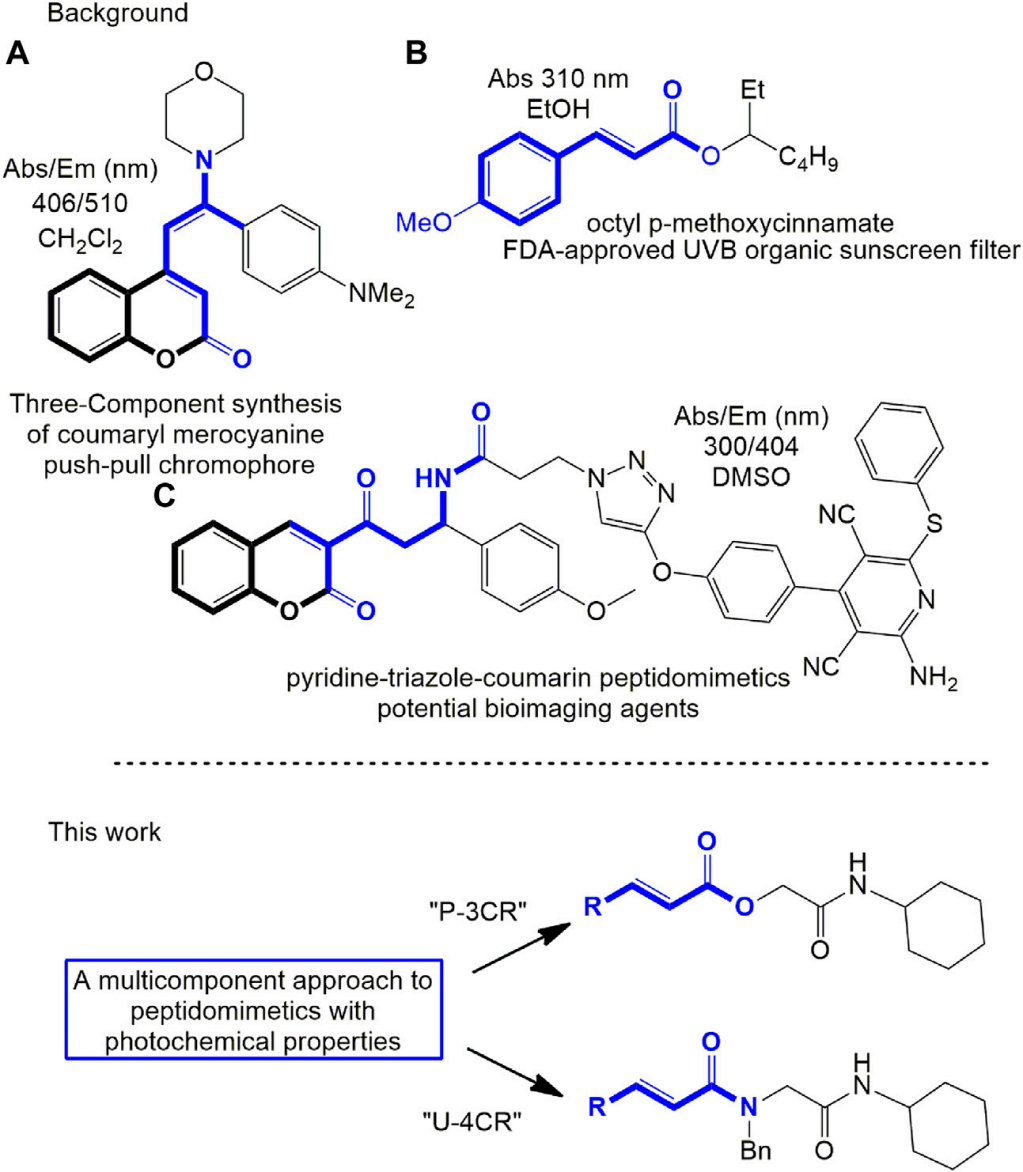
Some reported molecular compounds with their photophysical properties (top) and proposed work (bottom). **(A)** Functional chromophore using coumarin scaffold; **(B)** cinnamate derived as α,β-unsaturated carbonyl compound approved for FDA to UVB protection; **(C)** peptidomimetic that demonstrated potential use as a probe.

Peptidomimetics, a class of compounds that mimic the structure and function of peptides, have garnered significant attention from researchers in recent years owing to their vast array of potential applications. It is possible to find studies of peptidomimetics in various fields of investigation, such as the design of new enzyme inhibitors (Slobbe et al., 2012; Trabocchi and Guarna, 2014), antimicrobials (Váradi et al., 2015; Bicker and Cobb, 2020), antivirals, and antitumor agents (Wahby et al., 2022). Peptidomimetics have also been reported as chromophores (Soumya et al., 2017) (Figure 1C)) and fluorophores (Krishnan et al., 2013) used as biosensors (Peddie and Abell, 2019) and tailor-made probes for different analytes (Burguete et al., 2010). Thus, they have been considered in various lines of study in electronic organic chemistry, biomedical, and pharmaceutical chemistry (Barreto et al., 2014; Ganesh et al., 2017). However, it is worth noting that despite the growing interest in peptidomimetics, there remains a dearth of reported studies on their role in photochemistry research.

Due to their chemical functions and photochemical properties, different peptidomimetics have been studied, including pseudopeptide derivatives of anthracene (Burguete et al., 2010), highly functionalized 3-ethynyl quinoxaline scaffolds (Merkt and Müller, 2018), 3-substituted coumarin carboxamides (Sheikhhosseini et al., 2014), furo[2,3-c]isoquinoline substrates (Moni et al., 2016), triazolyl unnatural amino acids with aromatic chromophores (Bag et al., 2016), bioactive and fluorescent pyridine–triazole–coumarin (Soumya et al., 2017), thiazolino-fused 2-pyridones (Mohan and Bahulayan, 2017), triazole-linked chromenes (Krishnan et al., 2013), and others. Moreover, the interest in obtaining new potential compounds with these photophysical characteristics induces the need to develop efficient and advantageous synthesis methods; hence, the authors fundamentally use consecutive addition and cyclocondensation reactions, click chemistry, multicomponent reactions, or combinations (Moni et al., 2016; Soumya et al., 2017; Papadopoulos et al., 2018) (Figures 1A, C)).

Multicomponent reactions using isocyanides (IMCR) as starting material allow for high purity and structurally complex compounds to be obtained in a single reaction step (Dömling, 2006; Váradi et al., 2015); therefore, they have become essential in developing libraries of peptidomimetics (Farhid et al., 2021). Through the IMCRs developed by Mario Passerini (P-3CR) and Ivar Ugi (U-4CR), it is possible to obtain compounds where a carbonyl group can delocalize electrons and conjugate with different aromatic groups. This conjugation leads to important absorption bands and luminescence applications. These reasons have motivated the photochemical and photophysical study of products by IMCR in the field of applied photochemistry (Rocha et al., 2020).

Within the field of photochemical research, an issue that constantly draws attention is the study of photoprotection (protection from harmful sun rays, UVA, and UVB (280–400 nm)). UV radiation can penetrate deep into the skin and cause DNA damage (Placzek et al., 2007; Lippens et al., 2009; Sklar et al., 2013). To protect against these harmful effects, various classes of photoprotective compounds, commonly known as sunscreens, have been developed (Gilaberte and González, 2010; Gabros et al., 2022). Organic sunscreens are the most common photoprotective compounds. Usually, these compounds have a conjugated electron-withdrawing group (EWG), such as a carbonyl group attached to a π-conjugated group in their structure, which include aminobenzoates, cinnamates (Figure 1B)), salicylates, octocrylene, camphor derivatives, and others (Gabros et al., 2022). Thus, these molecules absorb the UV rays and release them as lower energy rays that are less toxic to the skin.

On the other hand, the luminescent properties of U-4CR adducts have been barely explored, and almost no information can be found about P-3CR derivatives in the last decade. The lack of evidence and few reports on the photochemical properties obtained through theoretical calculations is striking. Likewise, these studies are related to structural and complementary elucidation. Theoretical chemistry calculations can be used to predict the absorbent properties of peptidomimetics, which are related to their stereochemical characteristics.

In this work, we propose the development of new organic α,β-unsaturated carbonyl peptidomimetics using P-3CR and U-4CR (Figure 1). Specifically, we developed novel peptidomimetic molecules with functional chromophores such as cinnamic, coumarin, quinoline, and other derivatives. Using P-3CR and U-4CR, we obtained 13 depsipeptides and peptoids in moderate to high yields, varying the π electron-rich carboxylic acid as a reactant. We also study the photophysical properties of the synthesized compounds. We identified these compounds as photostable, and one showed strong fluorescence characteristics of its chemical functions. We also conducted cell toxicity tests on some compounds, revealing low toxicity levels even at high concentrations. Finally, we utilized time-dependent density functional theory (TD-DFT) theoretical calculations to describe electronic details and assign the absorption characteristics of the synthesized compounds.

## 2 Results and discussion

### 2.1 Synthesis and characterization of depsipeptides and peptoids

**FIGURE 2:**
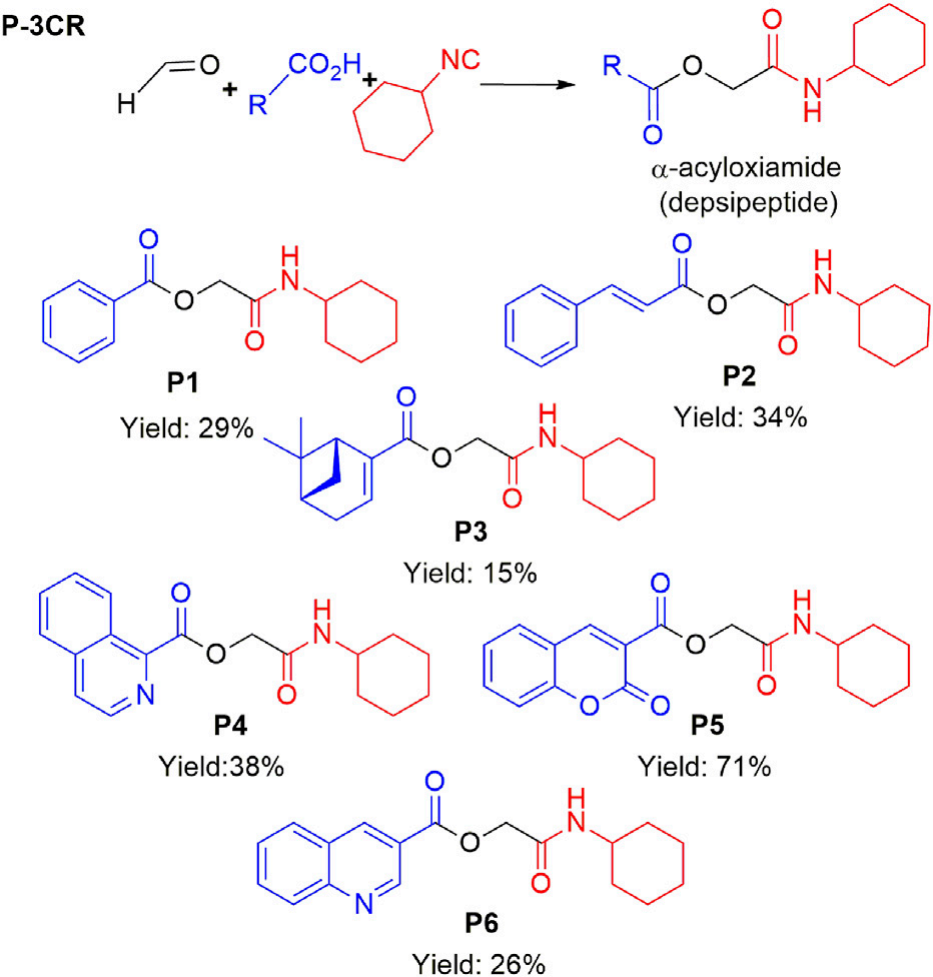
Compounds **P1**–**P6** synthesized by P-3CR.

The structural characteristics of the IMRC adducts, together with the selection of different molecular fragments that enhance their physicochemical properties, have motivated this research. Using P-3CR, six α,β-unsaturated carbonyl depsipeptides were synthesized using *p*-formaldehyde, cyclohexyl isocyanide, and the acids (R-CO_2_H): benzoic acid, cinnamic acid, (1*R*,5*S*)-6,6-dimethylbicyclo[3.1.1]hept-2-ene-2-carboxylic acid, isoquinoline-1-carboxylic acid, coumarin-3-carboxylic acid, and quinoline-3-carboxylic acid (**P1**–**P6,** respectively, in Figure 2) as the starting components, reaching yields between 15% and 71%.

Although the literature suggests that the P-3CR can be conducted in polar solvents like methanol (MeOH) or water (Pirrung and Sarma, 2004), we found that the reaction either did not occur or resulted in meager yields unless dichloromethane (DCM) was used as a solvent. Moreover, using high concentrations of these components was crucial for successful reactions. Despite these optimizations, the yields obtained were modest, with values only slightly higher than 30% for some products.

Other parameters were modified to promote the formation of Passerini products (Reza Kazemizadeh and Ramazani, 2012; Ramozzi and Morokuma, 2015). Higher amounts of acid (considering its effect as a catalyst) did not show the desired effect. A study on the relevance of applying temperature was also conducted. It was tried in the reactions using isoquinoline-1-carboxylic acid and coumarin-3-carboxylic acid, thus allowing obtaining a good yield of **P4.** The change of reagent *p*-formaldehyde for formaldehyde at 37% allowed for obtaining high yields for the **P5** derivative. For this mechanism, the lack of solubility of the components is a problem, while changing solvents to improve the solubility of these components was not an alternative. The P-3CR using derivatives with heterocycles and conjugated rings is a topic that needs further study since little has been reported on these compounds compared to other multicomponent reactions.

The synthesized α,β-unsaturated carbonyl depsipeptides were characterized using the necessary structure elucidation techniques (see Materials and Methods sections and Supplementary Section S2 at Supplementary Material). In NMR, the products showed the signals corresponding to the formation of the new bonds. The chemical shifts of carbon from the ester function and amide carbonyls formed at values between 164 and 167 ppm and the unshielded carbon (approx. 63 ppm). The singlet signal around 4.5–4.7 ppm integrates two protons corresponding to the central methylene groups. On the other hand, FT-IR allows us to demonstrate the formation of these new bonds with the appearance of two bands in the products belonging to the valence vibrations of the carbonyl bonds (ν_C=O_), one from ester and the other from amide with values close to 1,720 and 1,650 cm^−1^, respectively. Mass spectrometry also confirmed the formation of the compounds.

**FIGURE 3:**
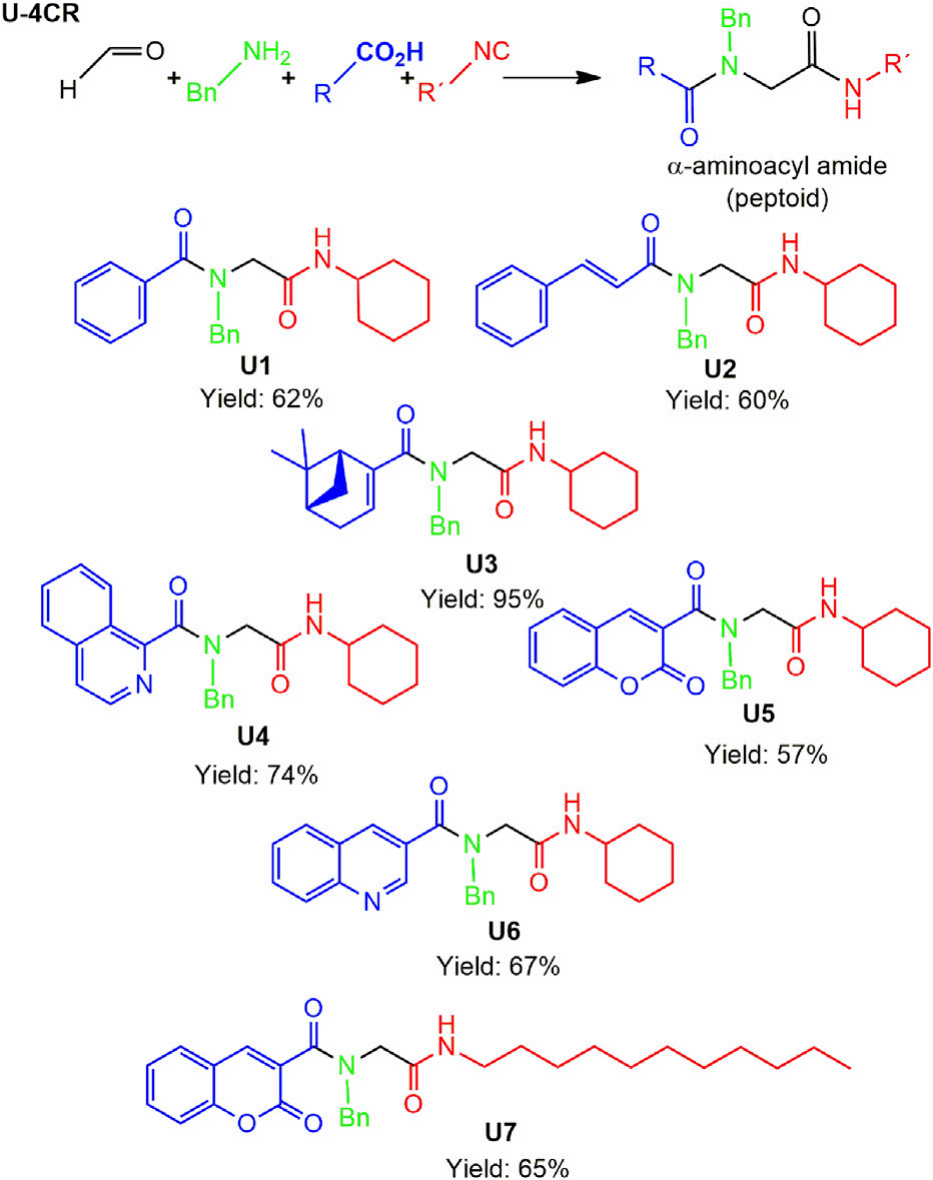
Compounds **U1**–**U7** synthesized by U-4CR.

A total of seven α,β-unsaturated carbonyl peptoids were synthesized through the U-4CR, using *p*-formaldehyde, benzylamine, cyclohexyl isocyanide (R′-NC) for **U1**–**U6**, and dodecyl isocyanide for **U7**, varying the carboxylic acids in order to generate the desired electronic delocalization and maintain other physical properties such as a balance between aromatic and aliphatic functions and not generating stereogenic centers. The selected carboxylic acids (R-CO_2_H) were benzoic acid for **U1** peptoid, cinnamic acid for **U2**, (1*R*,5*S*)-6,6-dimethylbicyclo[3.1.1]hept-2-ene-2-carboxylic acid for **U3**, isoquinoline-1-carboxylic acid for **U4**, coumarin-3-carboxylic acid for **U5**, quinoline-3-carboxylic acid for **U6,** and coumarin-3-carboxylic acids for **U7** (see Figure 3). To carry out the reactions, we utilized protic polar solvents (MeOH), which greatly enhanced the solubility of the reactants (Concepción et al., 2020). Reactions were also carried out at high concentrations (1 mM for components) for 24 h. The yields obtained from pure peptoids using U-4CR were greater than those from P-3CR, with the former achieving yields above 55%. This increase in yield can be attributed to several factors. First, the improved solubility of the starting acids in MeOH may have contributed to the overall efficiency of the reaction. Additionally, the proposed mechanisms suggest that the initial formation of the iminium ion (Rocha et al., 2020), a more reactive intermediate, may have played a role in the enhanced yields. Yields can even be significantly improved using 37% formaldehyde, and a subsequent test using coumarin-3-carboxylic acid generated yields of 90%, demonstrating the effect of the solubility of the reagents in the mechanism.

The different spectroscopic and spectrometric techniques allowed unequivocally assigning the formation of the desired products. The presence of functional groups in the products was verified with FT-IR spectroscopy. The synthesized molecules were confirmed with the bands related to the valence vibrations of the aromatic (ν_C=H_) and aliphatic (ν_C-H_) CH bonds, as well as the ν_C=O_ carbonyl bonds, the two bands of high intensity of about 1,650 cm^-1^ of the amides formed or of one of great intensity that probably belongs to both the carbonyl groups overlapping. NMR signals related to the new bonds were observed. The multiplet signal (due to its chemical environment) of the unshielded cyclohexyl proton was observed with a value close to 3.5 ppm. Due to their different chemical environment and stereoelectronic characteristics, several signals of the methylenes (diastereotopic protons) were in the region between 4 and 4.7 ppm. Chemical displacements at higher chemical shifts of the carbon atoms of the methine group of the cyclohexyl and the methylenes formed between amides were between 45 and 55 ppm for all peptoids.

### 2.2 Spectrophotometric analysis of depsipeptides and peptoids

**FIGURE 4:**
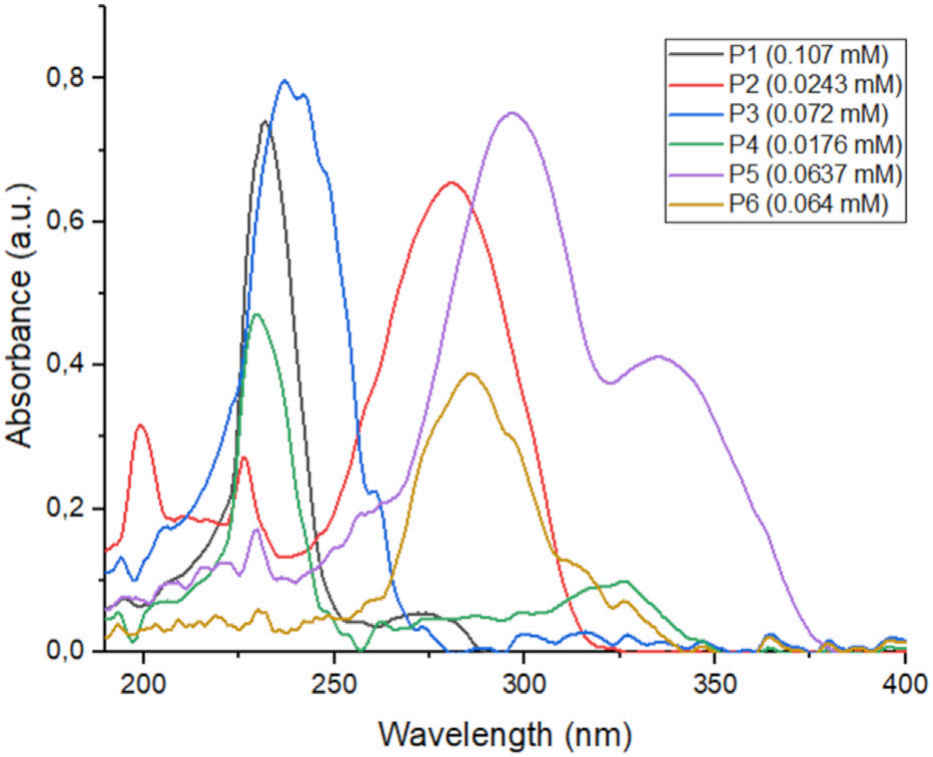
UV spectra of compounds **P1–P6** in DCM.

**FIGURE 5:**
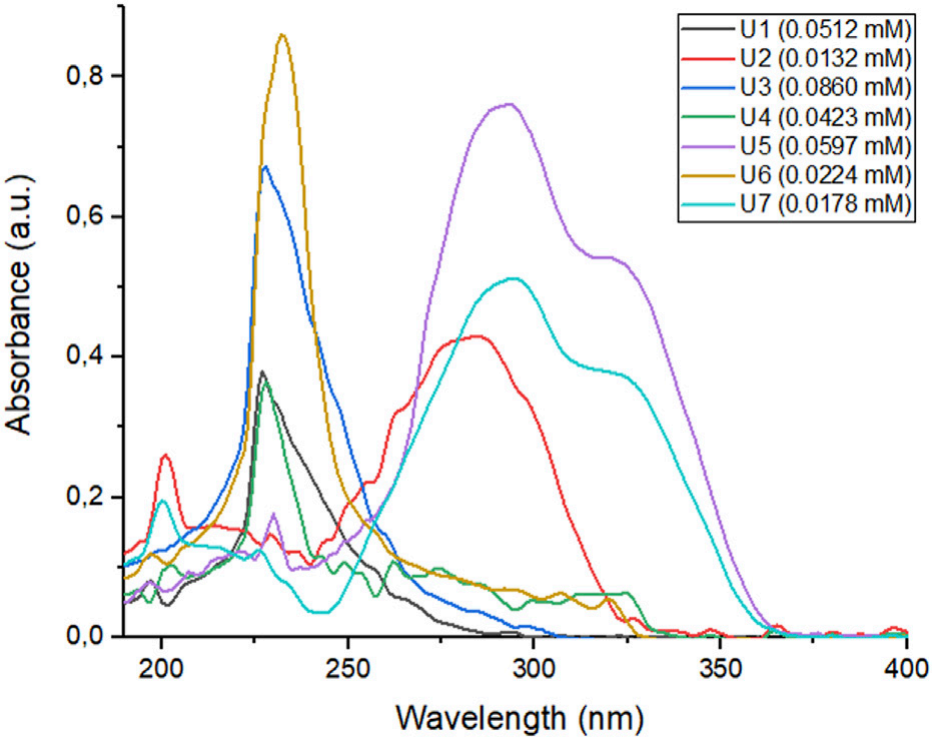
UV spectra of peptoids **U1**–**U7** in DCM.

Utilizing spectrophotometric analysis, we identified the principal absorption bands in the spectra of depsipeptides and peptoids. This analysis allowed us to gain a deeper understanding of the physico-chemical properties of these compounds. The spectrophotometric determination between the wavelengths 190 and 600 nm (UV/vis) demonstrated the strong absorption that all the compounds show in the UV region between 225 and 350 nm (see Figure 4 for depsipeptides and Figure 5 for peptoids). The spectra show that some compounds in less polar solvents retain fine structure in their absorption bands (e.g., spectra in hexane (Hex), see Supplementary Material). Furthermore, we observed that the polarity of the solvent has no significant effect on the maximum wavelength (λ_max_). However, it affects the intensity of the absorption bands, as anticipated. These effects are illustrated in Table 1, where shifts in the λ_max_ and band intensities are reported for different solvents. The present behavior is a negative solvatochromic effect. However, not being a marked effect, it could be assumed that the medium in which the compounds are dispersed or dissolved will not have a significant influence.

**TABLE 1:** Absorption parameters in different solvents of depsipeptides.

Depsipeptide		λ_max_ (nm)	*ξ*_max_ (L mol^−1^ cm^−1^)
P1	Hex	198	15,794
	DCM	232	6,915
	THF	228	13,612
	EtOH	227	17,342
	MeCN	194	41,090
P2	Hex	273	44,615
	DCM	281	26,955
	THF	276	22,251
	EtOH	278	10,344
	MeCN	272	48,390
P3	Hex	194	10,101
	DCM	237	11,083
	THF	231	9,783
	EtOH	233	4,639
	MeCN	192	16,020
P4	Hex	221	36,527
	DCM	229	26,704
	THF	220	57,840
	EtOH	220	57,361
	MeCN	192	47,787
P5	Hex	290	1,948
	DCM	297	5,386
	THF	294	12,500
	EtOH	296	12,772
	MeCN	292	16,373
P6	Hex	-	ND
	DCM	286	6,046
	THF	291	5,169
	EtOH	281	10,113
	MeCN	281	4,652

ND, not determined due to poor solubility of the compound.

It is worth noting the spectra of the depsipeptide **P2** has a wide absorption band at λ_max_ = 280 nm with a high value of molar extinction coefficient (*ξ* = 26,955 L mol^−1^ cm^-1^), a region included in the UV region of UVC (<280 nm) and UVB (280–315 nm) radiation. This absorption profile is probably due to the less energetic electronic transition between the nπ* orbitals. The band loses intensity with increasing polarity of the solvents, while in MeCN, the bands related to the ππ* transitions are more intense and predominant (see Supplementary Material).

**P5** and **P6** possess the most prominent bathochromic effect among the depsipeptides. Both have absorption maxima above 280 nm, so they can absorb in the UVB region, while **P5** can also absorb in the UVA region. Furthermore, the transition bands of the nπ* and ππ* orbitals are of great intensity.

According to our objective, α,β-unsaturated carbonyl depsipeptides showed displacements that would allow us to consider them strong candidates as photoprotectors. Nevertheless, we consider that, through this synthetic method, obtaining other derivatives by moderately modifying their components is possible due to their simplicity and convenience. Recent studies have shown that depsipeptides can be synthesized with maximum wavelengths in the UVB region and important absorption bands in the UVA region. These properties can be achieved by introducing methoxy or amino groups to the aromatic fragment, which causes significant bathochromic shifts (Kuziv et al., 2020) (although it is not our objective to obtain candidates with absorption at long wavelengths, but rather ones that cover the region of the UVB and UVA electromagnetic spectrum, to be considered as potential broad-spectrum sunscreen). However, it is essential to note that these modifications should not result in the emission of light radiation, which could have adverse effects.

Peptoids showed strong absorption bands at longer wavelengths (Table 2); **U2** (λ_max_ = 287 nm) and **U5** (λ_max_ = 294 nm) had a high-intensity band between 250 and 350 nm in DCM (Figure 5). This is a characteristic to consider due to the possibility of generating protection against UV radiation from the UVB and UVA regions.

**TABLE 2:** Absorption parameters in different solvents of peptoids.

Peptoid		λ_max_ (nm)	*ξ*_max_ (L mol^−1^ cm^−1^)
U1	Hex	193	25,047
	DCM	227	7,407
	THF	213	18,175
	EtOH	203	41,171
	MeCN	193	59,115
U2	Hex	281	12,260
	DCM	287	32,500
	THF	212	27,071
	EtOH	282	20,330
	MeCN	277	24,973
U3	Hex	194	24,172
	DCM	228	7,755
	THF	214	12,763
	EtOH	202	20,495
	MeCN	191	57,777
U4	Hex	219	26,778
	DCM	227	8,605
	THF	220	68,160
	EtOH	219	66,790
	MeCN	217	23,505
U5	Hex	191	9,541
	DCM	294	12,747
	THF	294	11,594
	EtOH	288	11,134
	MeCN	286	9,826
U6	Hex	233	2,137
	DCM	233	38,392
	THF	230	90,236
	EtOH	228	34,859
	MeCN	191	84,594
U7	Hex	193	14,792
	DCM	295	28,764
	THF	212	18,227
	EtOH	288	13,425
	MeCN	202	8,173

Peptoid **U7** also presents a large absorption band between 250 and 350 nm, with a maximum wavelength of 291 nm. This compound presents an absorptive behavior similar to the analogous compound **U5**, where the main band is found in this spectrum region. Its intensity decreases as the polarity of the medium increases, where the most intense band moves to the most energetic region due to solvation. Both peptoids have coumarin, a fragment with which bands can be obtained related to the electronic delocalization product of the new amide bond and the electrons of the double bond that is part of the lactone that has this EWG effect (negative mesomeric effect). This effect is more pronounced in less polar solvents such as DCM; in turn, a hypsochromic effect can be seen by increasing the solvents’ polarity (see Supplementary Material).

Avobenzone is a photoprotective UVA, approved by the FDA and generally taken as a comparative standard due to its high absorptive efficacy. This product presents absorption bands with maximums around 360 nm, but when the medium’s polarity increases, its spectrum changes to maximum shorter lengths (Mturi and Martincigh, 2008). **U2**, **U5**, and **U7** peptoids’ absorption spectrum in DCM are comparable to the avobenzone spectrum and its behavior in different solvents.

### 2.3 Emission results

Considering the potential of the synthesized derivatives, we studied their other photochemical properties, also remembering that the organic compounds that could potentially be used as sunscreens should not present other luminescent properties. The UV ideal absorber aims to dissipate the exceeding energy by internal conversion or vibrational relaxation, avoiding the slower radiative mechanism like fluorescence. The emissions of some photoprotectors are related to the induction of melanoma and aging (since some skin components can be photoexcited, for example, collagen) (Losantos et al., 2017).

**FIGURE 6:**
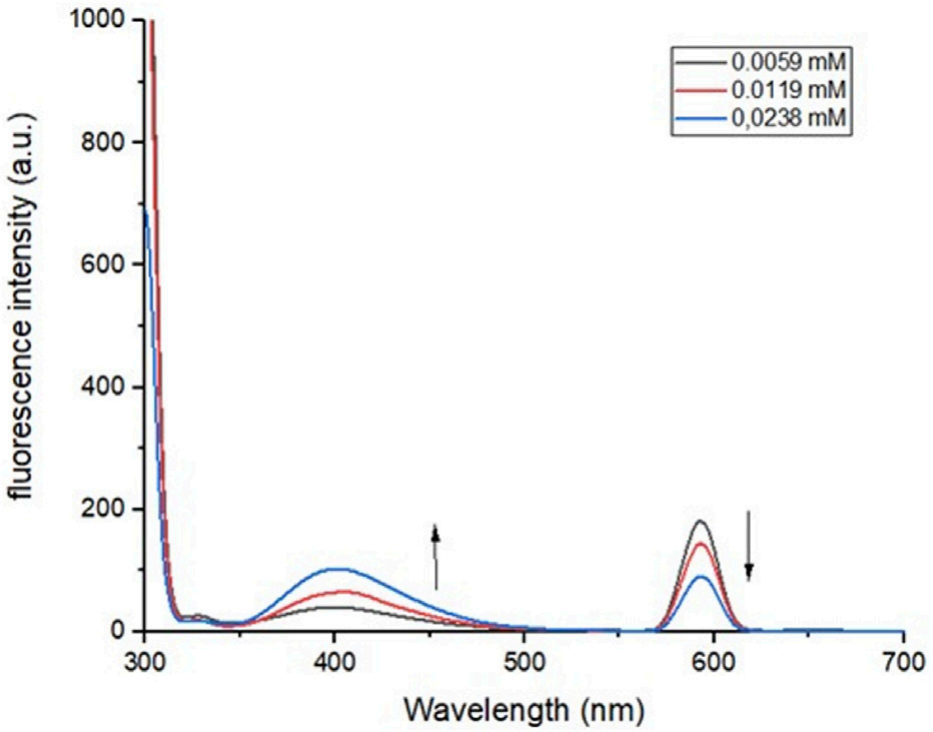
PL spectra of peptoid **U5** at different concentrations in DCM, λ_exc_ = 280 nm.

The solutions of the compounds are all colorless. Our primary goal was that our compounds did not show emission through fluorescence. Only **U6** of the IMCR derivatives synthesized turned out to have fluorescence after excitation by exposure to a UV lamp (Supplementary Figure S3.1 in the Supplementary Material). The fluorescence emission (PL) spectra were also measured for **P2**, **P5**, **U5**, and **U6** with excitation at the absorption maximum wavelength (Table 3; more results in Supplementary Material). They showed emission bands with negligible intensity values, and the fluorescence bands with the highest PL λ_max_ decreased as the concentration increased. The other band had the opposite effect (it became more intense when the concentration increased; Figure 6). Hanson et al. (2015) described similar processes under the concept of aggregation when the concentration increases (without reaching a concentration that causes quenching), which could even lead, according to their reports, to an increase in fluorescence.

**TABLE 3:** Some previous emission results for peptidomimetics (c = 0.01 mM).

Compound	PL λ _max_ (nm)	PL intensity (a.u.)	Stokes shift (nm)
P2	337; 568	27; 82	56; 287
**DCM**			
P5	312; 568	47; 390	86; 164
**DCM**			
U5	404; 593	65; 143	110; 299
**DCM**			

The **U6** peptoid presented a blue emission with a low intensity band with a maximum at 478 nm in DCM and 453 nm in EtOH, which increases slightly with increasing concentration, in the prepared solution samples until the increase in concentration causes a quenching of the fluorescence signal.

The pharmaceutical and cosmetic industry need to develop new products that, in addition to having a role as photoprotectors, can be used to determine the quality parameters for the evaluation as photoprotectors (e.g., *in vitro* UVA protection labeling (UVA-PF) determination). Many of these *in vitro* methods require the development of new protocols that allow estimation of these parameters. Some of these studies are based on fluorescent estimation using different additives to the formulation (Hojerová et al., 2011; Hernández-Rivera et al., 2021).

### 2.4 Photostability study

Since the photostability against UV radiation is one of the parameters measured in a photoprotective candidate (Mturi and Martincigh, 2008), we carried out an *a priori* test of the stability of our compounds exposed to different UV radiations (254 and 365 nm). Following the protocols established for the photostability studies described in the ICH harmonized tripartite guideline (Tsujita-Inoue et al., 2016), at a controlled temperature and using an artificial UV lamp, we exposed the compounds by measuring them at different times. The **U5** solid sample was submitted to this study. After the exposure times, they were dissolved in DCM at a final concentration of approximately 30 μM for reading. This procedure is widely used to monitor the changes that the effect of radiation can produce. Then, their UV spectra were measured, we were unable to observe spectrophotometric differences (UV spectrum and HPLC-DAD chromatograms for **U5** in Supplementary Material), which allows us to suggest that the compounds did not experience structural changes and they remain stable. On the other hand, to verify the effect of radiation, we obtained the chromatograms of the compound **U5** in solution (TFA 0.01%/MeCN) using HPLC-DAD before and after exposure to the UV lamp (1 and 3 h). This sensitive method showed us that the chromatogram signal does not change, indicating the existence of the same compound and not photodegradation derivatives under the test conditions.

### 2.5 Cytotoxicity assay

**FIGURE 7:**
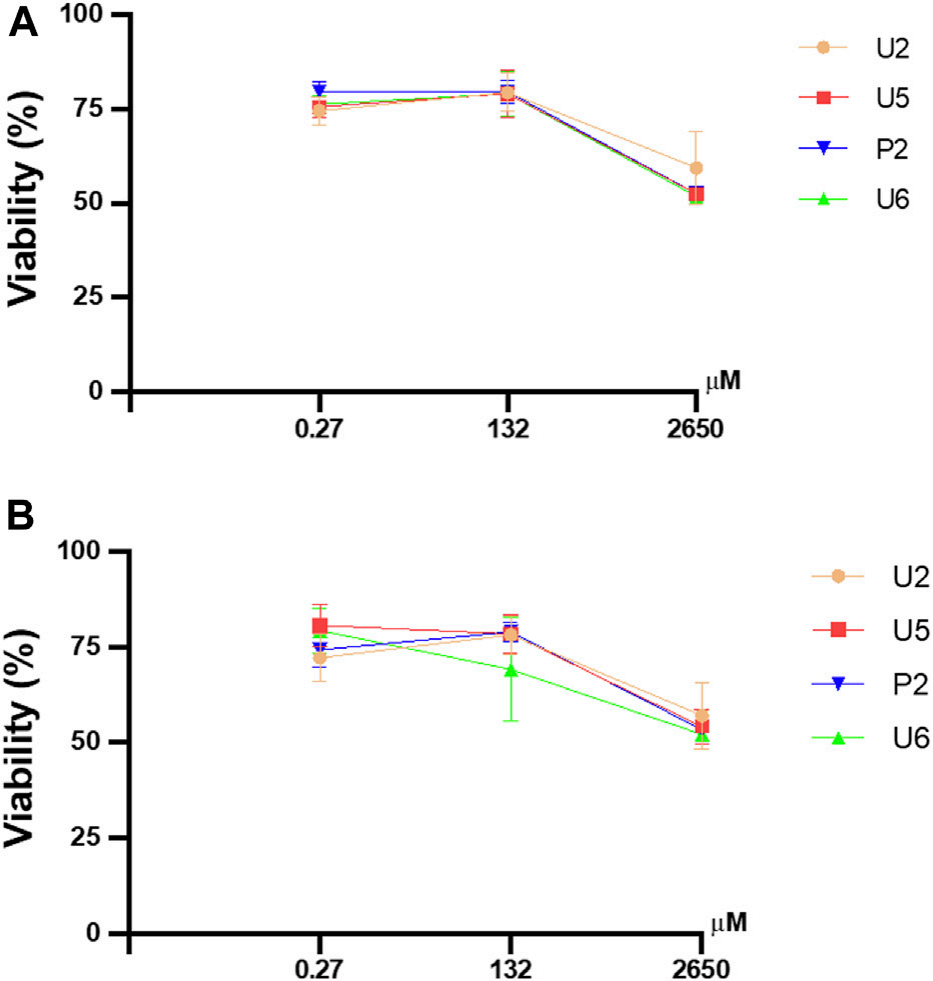
Effect of selected compounds on cell viability using the CCK-8 assay in HEK293 **(A)** and HeLa **(B)** cell lines. Concentrations used were 0.27, 132, and 2,650 µM. All compounds were used for 24 and 48 h. All values are means ± S.D (*n* = 3). SD: standard deviation.

Compounds **U2**, **U5**, **U6,** and **P2** were selected for cytotoxicity analysis based on the photostability results obtained. Figure 7 shows the results of the cytotoxicity assay of the selected compounds expressed as the percentage of cell viability on human cell lines (HEK293 and HeLa). We used concentrations of the compounds that were 100 times higher than those used in the photostability study and tested them under 24- and 48-hour conditions. However, the results indicated no significant differences in cell viability with tested compounds, as the viability remained over 75%. Therefore, these compounds are nontoxic to human HEK293 and HeLa cell lines. Furthermore, all compounds exhibited similar behavior and were highly cyto-compatible over a 24-hour period, which is the average time that a sunscreen stays on the skin.

### 2.6 Computational calculations analysis

For the calculation of vertical excitations, the ground state optimized geometries were subjected to TD-DFT computations for the first 10 states using the CAM-B3LYP/6–311g(d,p) method. The ground state minimum energy geometries of the selected structures (**P2**, **U2**, **P5**, and **U5**, depicted on Figures 2, 3) were optimized using the hybrid functional CAM-B3LYP at the 6–311g(d,p) basis set (Yanai et al., 2004). The polarizable continuum model using the integral equation formalism variant (IEFPCM) (Cancès et al., 1997; Mennucci et al., 1997) was used to optimize the ground and excited state geometries using the different solvents described in the experimental procedure to take into account structural variations due to the solvent effect if they existed. The solvents used were DCM, EtOH, Hex, MeCN, and THF. The vertical excitation energies and oscillator strength for the lowest excited states were calculated using these ground state minimum energy geometries at the TD-DFT/CAM-B3LYP/6–311g(d,p) level of theory (Yanai et al., 2004; Dreuw and Head-Gordon, 2005).

**FIGURE 8:**
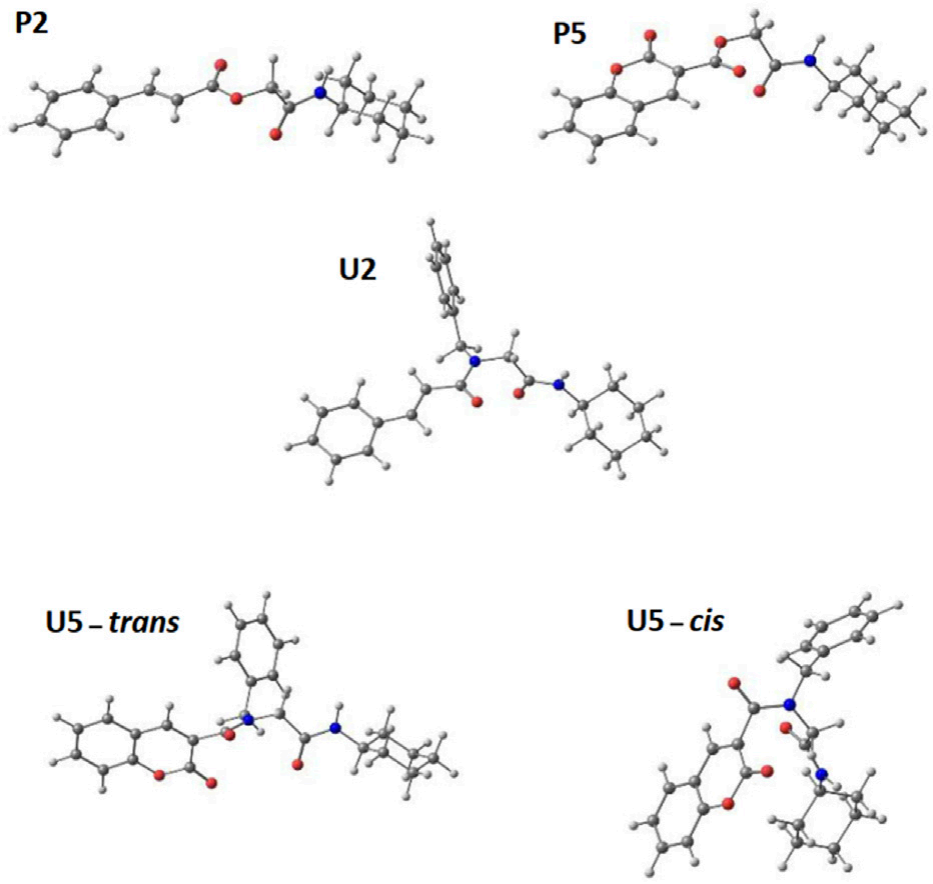
Optimized structures for selected α,β-unsaturated carbonyl peptidomimetics **P2**, **P5**, **U2**, and **U5**.

We explored preferences for conformers of the selected compounds. In **P2** and **P5**, the central C-O-CH_2_-C dihedral angle was rotated, while in **U2** and **U5**, the C-N-CH_2_-C dihedral angle was rotated. In this process, each angle was increased by 10° and then optimized to obtain conformers with lower energy. Figure 8 shows that the most probable rotational isomers were different for **P2** and **P5** when the relative positions of the carbonyl groups were compared. In **P2,** the carbonyl groups were situated *trans* to each other, but **P5** prefers the *gauche* isomer. The preponderance of the *gauche* isomer could contribute to a larger dipole moment for **P5**. The optimized structures of **U2** and **U5** were analyzed according to the *N*-alkylated *cis*–*trans* amide isomerization (Torre et al., 2019). A *trans* amide isomer was the optimized structure for **U2**. On the other hand, optimized structures of *trans* and *cis* isomers were obtained for **U5**.

The most probable transitions of all compounds in different solvents, which account for the highest oscillator strength, are listed in Table 4. The variations in the absorption maxima of the compounds of the solvents used for the calculation do not show any influence of the polarity, nor the change of intensity between the bands that can be observed in the experimental spectra. The transitions are characterized as HOMO–LUMO type transitions (100% HOMO–LUMO contribution for **P2**, **P5,** and **U5,** and more than 90% for **U2**). The oscillator strength values for **P2** and **U2** are around 0.9, and these values for **P5** and **U5** are around 0.4, corresponding to HOMO–LUMO transitions at 275, 276, 307, and 294 nm for **P2**, **U2**, **P5**, and **U5**, respectively. These calculated transitions are in agreement with the experimental UV radiation spectra for these compounds reported in the Supplementary Material (Supplementary Figure S2.8 (**P2**), S2.32 (**U2**), S2.20 (**P5**), and S2.41 (**U5**)).

**TABLE 4:** Observed absorption and computed vertical excitation energies for the most probable transitions of compounds P2, P5, U2, and U5, with largest oscillator strength, in different solvents.

Dye	Solvent	Expt λ_max_ (nm)	TD/CAM-B3LYP/6-311g**			
			Vertical excitation		Oscillator strength (*f*)	H–L Orbital contribution
			nm	eV		
P2	Hex	273	274.5	4.51	0.907	100%
	THF	276	276.2	4.48	0.920	100%
	DCM	281	276.5	4.48	0.923	100%
	EtOH	278	276.0	4.49	0.915	100%
	MeCN	272	275.8	4.49	0.913	100%
P5	Hex	290	307.6	4.03	0.378	100%
	THF	294	307.0	4.03	0.380	100%
	DCM	297	307.1	4.03	0.383	100%
	EtOH	296	306.4	4.04	0.370	100%
	MeCN	292	306.3	4.04	0.367	100%
U2	Hex	281	276.5	4.48	0.847	90.4%
	THF	215	277.0	4.47	0.951	96.8%
	DCM	287	277.2	4.47	0.959	96.8%
	EtOH	282	276.6	4.48	0.958	96.8%
	MeCN	277	276.4	4.48	0.956	96.8%
U5-*trans*	Hex	-	295.4	4.19	0.423	100%
	THF	294	293.9	4.21	0.435	100%
	DCM	294	294.0	4.21	0.438	100%
	EtOH	290	293.1	4.23	0.429	100%
	MeCN	286	292.8	4.23	0.426	100%

For the compounds **P2**, **U2**, **P5**, and **U5,** the state (in each solvent) described in Table 4 is the first and lowest in energy, while those lacking emission may have other states with no oscillator strength lower in energy. This characteristic is important for the application of these chemical compounds as sunscreens since sunscreens are designed to protect the skin from harmful UV radiation, which can cause various health issues such as sunburn, premature aging, and an increased risk of skin cancer. The lack of emission in chemical compounds used as sunscreens ensures that they effectively absorb or scatter UV radiation without introducing any additional harmful effects to the skin. This property allows sunscreens to provide reliable and safe protection against the damaging effects of UV radiation.

**FIGURE 9:**
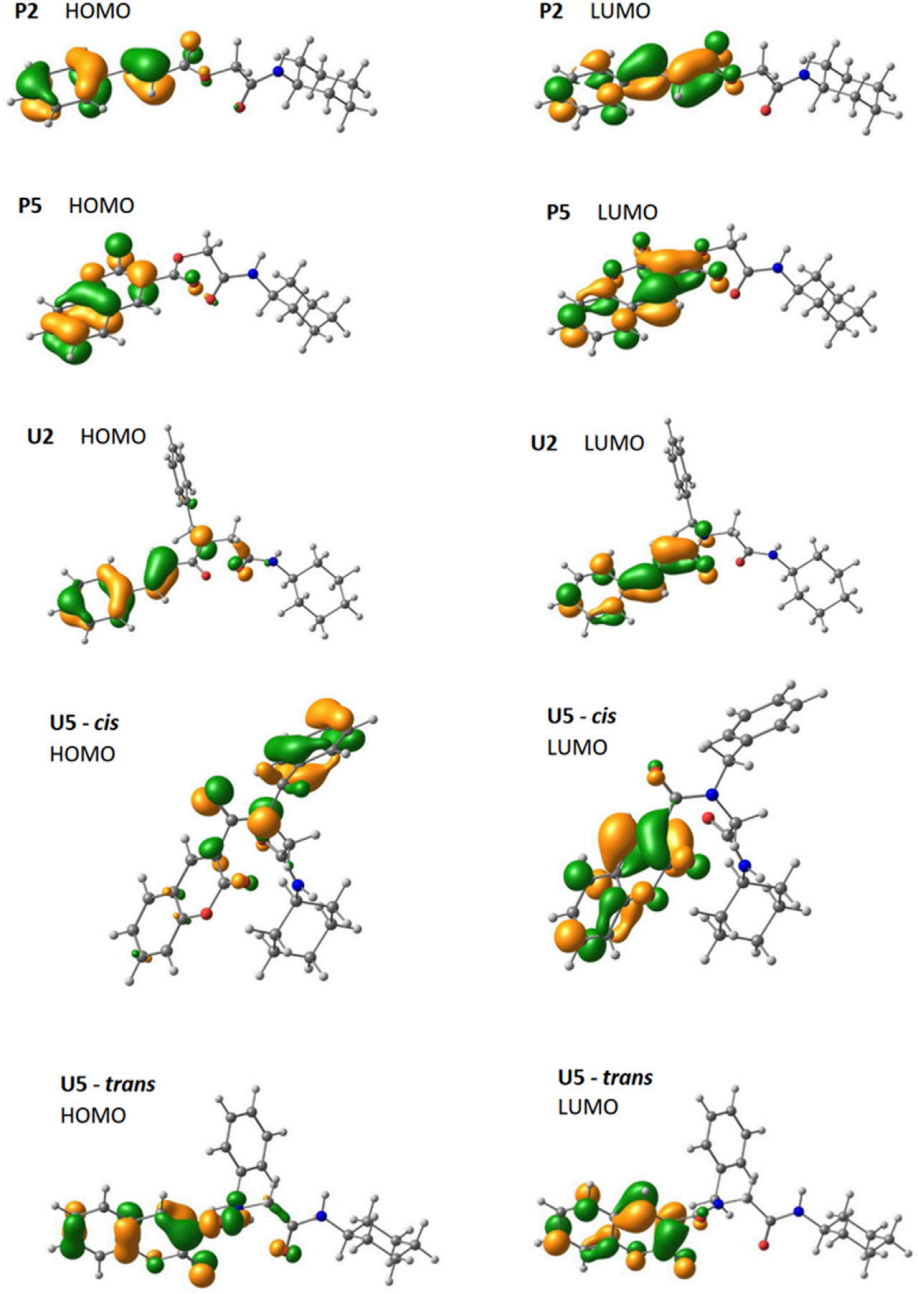
Optimized frontier orbital representations (contour value = 0.05) for **P2**, **P5**, **U2**, and conformers *cis*–*trans* of **U5** (using the CAM-B3LYP/6–311g(d,p) method).

The frontier molecular orbitals are depicted in Figure 9. For the four compounds that presented important transitions in the region of interest, the frontier orbitals always appear to be located in the fragment that comes from the component cinnamic acid (**P2** and **U2**) and coumarin acid (**P5** and **U5**), as expected. However, if we compare the *trans* and *cis* isomers for **U5**, there is a change in the location of the HOMO that appears centered on the benzyl group in **U5-*cis***. That means that there is a change in the charge transfer direction upon the formation of that isomer (and possibly in magnitude).

The HOMO and LUMO orbitals are similar for **P2** and **U2**. In both compounds, the HOMO orbital has three contributions in the cinnamic group. The first two are in the phenyl connecting positions 3, 4, and 5 and positions 2, 1, and 6. The third connects the two carbons of the alkene. The LUMO orbital for both compounds has localized contributions at positions 2, 4, and 6 of phenyl and another contribution connecting carbon 1 of phenyl to the nearest alkene carbon. It also presents another contribution that connects the other carbon of the alkene with the carbonyl carbon, and the last one is located in the oxygen of the carbonyl.

The HOMO and LUMO orbitals for **P5** and **U5** are located on coumarin, except for the HOMO orbital of **U5-*cis***, which is located on benzylamino, as mentioned previously. In **P5** and **U5-*trans***, the HOMO orbital has contributions connecting coumarin carbons (one connects 6 and 7, and the other connects 4a and 8a). It also has localized contributions to oxygen 1 and carbonyl oxygen. A final HOMO contribution on coumarin is located at carbon 3 in **P5** and extends between 3 and 4 in **U5-*trans***. In **P5**, **U5-*cis***, and **U5-*trans***, the LUMO orbital also has contributions connecting coumarin carbons (one connects 2 and 3, another connects 4 and 4a, and the other connects 8 and 8a). It also has localized contributions at carbons 5 and 7, at oxygen 1, and at the carbonyl oxygen.

## 3 Conclusion

In summary, our study has led to the development of novel α,β-unsaturated carbonyl depsipeptides and peptoids, which can be synthesized and isolated in a good yield using one-pot multicomponent reactions (through P-3CR and U-4CR) with functional chromophores. Different groups were included by varying the electron-rich carboxylic acid reactants used in the synthesis. The compounds demonstrated promising photophysical and electronic properties, low toxicity levels with the cellular line used, and potential for further applications in various fields. For example, we remarked that compounds **P2**, **P5**, **U2**, and **U5** can be considered for study as photoprotectors, specifically due to their absorption in the UVB and UVA region and their photostability properties. On the other hand, **U6** turned out to present a light-type emission, a characteristic that allows it to be considered a fluorescent probe.

Theoretical calculations were used to rationalize the electronic structures of the selected compounds and their electronic transitions that explain the observations in the UV spectra. For the calculation of vertical excitations, the optimized ground state geometries of the selected structures **P2**, **P5**, **U2**, and **U5** were subjected to TD-DFT calculations using the CAM-B3LYP method. Different solvents were utilized, and the most probable rotational isomers for the selected compounds were reported. The most probable transitions of these compounds in different solvents, which account for the greater oscillator strength, were determined. The absorption maxima of the compounds showed minimal variation across the solvents, indicating little influence of polarity or changes in intensity between the bands observed in the experimental spectra. The calculated transitions are in agreement with the experimental UV radiation spectra.

## 4 Materials and methods

The materials and reagents were of the highest commercially available grade and used without further purification. The melting points (uncorrected) were determined using a Fisher–Johns melting point apparatus (Bibby Scientific Limited, Staffordshire, UK). ATR-FT-IR spectra were recorded by using UATR two (Beaconsfield, Bucks, UK) in the range of 4,000–400 cm^−1^ with 16 scans. ^1^H-NMR and ^13^C-NMR spectra were recorded at 400 MHz for ^1^H and 100 MHz for ^13^C, respectively, using DMSO or CDCl_3_ as solvents. Chemical shifts (δ) are reported in ppm relative to the residual solvent signals, and coupling constants (*J*) are reported in Hz. High-resolution mass spectra (HRMS) were obtained from Thermo Fisher Scientific Exactive Plus mass spectrometer. The analysis was performed at heater temperature, 50°C; sheath gas flow, 5; sweep gas flow rate, 0; and spray voltage, 3.0 kV in the negative mode. The accurate mass measurements were performed at a resolution of 140,000. Experimental UV spectra were recorded in a Spectroquant® Pharo 300 from 190 nm to 600 nm every 1 nm for each compound in five solvents (Hex, DCM, tetrahydrofuran (THF), ethanol (EtOH), and acetonitrile (MeCN)). These spectra were stored in digital format in a spreadsheet, one for each compound, using OriginPro version 2019b. Using a spreadsheet template, spectra were transformed based on normalized absorbance or based on molar extinction coefficients versus wavelength in nm to allow a better observation of the comparisons made. A fluorescence study was performed using a HITACHI F-2700–2710 fluorimeter. EtOH and DCM were used as solvents, and measurements were taken by exciting the samples at the length of maximum absorption. HPLC-DAD was performed on the YL9100 equipment, manufactured by YL Instrument Co., LTD.

### 4.1 General procedure for the P-3CR

The depsipeptides were prepared according to the literature procedure (Wahby et al., 2022). The *p*-formaldehyde (1.0 mmol, 1.0 equiv.), the respective carboxylic acid (1.0 mmol, 1.0 equiv.), and the cyclohexyl isocyanide (1.0 mmol, 1.0 equiv.) were added to DCM (5 mL) for **P1**–**P6,** and the mixture was stirred at 25°C for 24 h. The crude reaction products were treated first with a saturated citric acid solution, then a saturated sodium bicarbonate solution, and finally with a saturated sodium chloride solution. After that, they were purified by flash column chromatography (Hex/ethyl acetate (EtOAc) 4:1 v/v).

### 4.2 General procedure for the U-4CR

The peptoids were prepared according to the literature procedure (Dömling, 2006; Sharma et al., 2015), with some modifications. A solution of the bencylamine (1.0 mmol, 1.0 equiv.) and *p*-formaldehyde (1.0 mmol, 1.0 equiv.) in MeOH (5 mL) was stirred at 25°C for 1 h. Then, the respective carboxylic acid (1.0 mmol, 1.0 equiv.) and the corresponding isocyanide (1.0 mmol, 1.0 equiv.) were added and left to react for 24 h. The crude products of reactions were treated first with a solution of citric acid, then with a sodium bicarbonate solution, and finally with a saturated sodium chloride solution. After that, they were purified by flash column chromatography (Hex/ethyl acetate (EtOAc) 3:2 v/v).

#### 4.2.1 2-(Cyclohexylamino)-2-oxoethyl benzoate


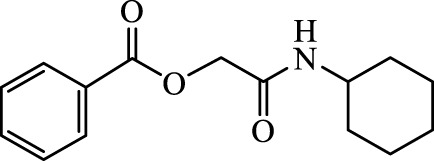


Depsipeptide **P1** (75 mg, 29%) as a white solid. Rf = 0.25 (Hex/EtOAc 3:1 v/v). Mp = 128.6°C–129.7°C. UV (DCM, λ_max_ = 232 nm). IR (KBr) 3,294, 3,070, 2,931, 2,850, 1735, 1,658, 1,554, 1,450, 1,415, 1,288, 1,230, 1,176, 1,130, 702, 570 (cm^−1^). ^1^H NMR (400 MHz, CDCl_3_) *δ* 7.93–7.68 (m, 2H), 7.36 (t, *J* = 7.4 Hz, 1H), 7.23 (t, *J* = 7.7 Hz, 2H), 5.97 (d, *J* = 5.7 Hz, 1H, NH), 4.51 (s, 2H, CH_2_), 3.64–3.45 (m, 1H, CH), 1.75–1.58 (m, 2H), 1.51–1.29 (m, 3H), 1.20–0.86 (m, 5H). ^13^C NMR (100 MHz, CDCl_3_) *δ* 166.49, 165.41, 133.71, 129.69, 128.96, 128.67, 63.35, 48.17, 32.83, 25.35, 24.69. HRMS (ESI–TOF) m/z: 284.1245 [M + Na]^+^; calcd. for C_15_H_19_NO_3_Na, 284.1263.

#### 4.2.2 2-(Cyclohexylamino)-2-oxoethyl cinnamate


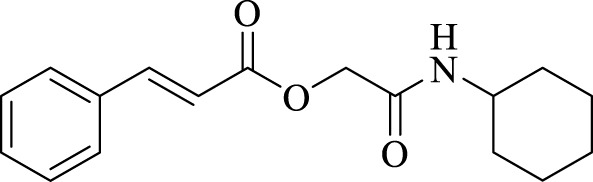


Depsipeptide **P2** (98 mg, 34%) as a white solid. Rf = 0.20 (Hex/EtOAc 3:1 v/v). Mp = 135.7°C–137.6°C. UV (DCM, λ_max_ = 282 nm). IR (KBr) 3,285, 2,927, 2,851, 2,113, 1,670, 1,626, 1,559, 1,418, 1,310, 1,281, 1,219, 1,169, 975, 910, 765, 698 (cm^−1^). ^1^H NMR (400 MHz, CDCl_3_) δ 7.80 (d, *J* = 16.0 Hz, 1H), 7.61–7.55 (m, 2H), 7.46–7.41 (m, 3H), 6.53 (d, *J* = 16.0 Hz, 1H), 6.03 (d, *J* = 7.2 Hz, 1H, NH), 4.70 (s, 2H, CH_2_), 3.87 (ttd, *J*_*1*_ = 12.0, *J*_*2*_ = 8.2, *J*_*3*_ = 3.9 Hz, 1H, CH), 2.01–1.92 (m, 2H), 1.80–1.69 (m, 2H), 1.69–1.60 (m, 1H), 1.48–1.33 (m, 2H), 1.29–1.13 (m, 3H). ^13^C NMR (100 MHz, CDCl_3_) δ 166.12, 165.47, 146.70, 133.94, 130.85, 129.02, 128.29, 116.47, 63.14, 48.01, 33.03, 25.45, 24.81. HRMS (ESI–TOF) *m/z*: 310.1420 [M + Na]^+^; calcd. for C_17_H_21_NO_3_Na, 310.1419.

#### 4.2.3 2-(Cyclohexylamino)-2-oxoethyl (1*R*,5*S*)-6,6-dimethylbicyclo[3.1.1]hept-2-ene-2-carboxylate


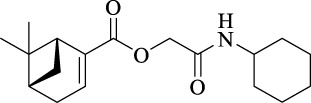


Depsipeptide **P3** (47 mg, 15%) as white solid. Rf = 0.40 (Hex/EtOAc 3:1 v/v). Mp = 85.9°C–88.3°C. UV (DCM, λ_max_ = 236 nm). IR (KBr) 3,256, 3,085, 2,928, 2,863, 1,716, 1,654, 1,237, 1,448, 1,103, 953, 888, 749(cm^−1^). ^1^H NMR (400 MHz, CDCl_3_) δ 6.90–6.87 (m, 1H), 5.93 (d, *J* = 6.8 Hz, 1H, NH), 4.57 (q, *J* = 15.4 Hz, 2H), 3.86–3.76 (m, 1H), 2.78 (td, *J* = 5.7, 1.3 Hz, 1H), 2.55–2.37 (m, 2H), 2.16 (dd, *J*_*1*_ = 12.7, *J*_*2*_ = 1.0 Hz, 1H), 1.94–1.81 (m, 2H), 1.71–1.56 (m, 2H), 1.41–1.10 (m, 11H), 0.78 (s, 3H). ^13^C NMR (100 MHz, CDCl_3_) δ 166.44, 164.46, 139.38, 138.17, 62.93, 47.72, 41.35, 40.18, 37.70, 32.90, 32.34, 31.24, 25.81, 25.43, 24.60, 20.96. HRMS (ESI–TOF) *m/z*: 328.1889 [M + Na]^+^; calcd. for C_18_H_27_NO_3_Na, 328.1889.

#### 4.2.4 2-(Cyclohexylamino)-2-oxoethyl isoquinoline-1-carboxylate


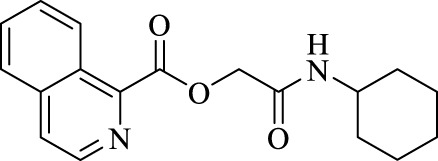


Depsipeptide **P4** (118 mg, 38%) as white solid. Rf = 0.30 (Hex/EtOAc 3:1 v/v). Mp = 126.5°C–128.8°C. UV (DCM, λ_max_ = 230 nm). IR ((KBr) 3,287, 3,050, 3,000, 2,928, 2,881, 2,099, 1726, 1,651, 1,544, 1,438, 1,243, 1,138, 979, 836, 794 (cm^−1^). ^1^H NMR (400 MHz, CDCl_3_) δ 8.76 (d, *J* = 8.5 Hz, 1H), 8.63 (d, *J* = 5.5 Hz, 1H), 7.92 (dd, *J*_*1*_ = 11.6, *J*_*2*_ = 6.8 Hz, 2H), 7.76 (dtd, *J*_*1*_ = 16.6, *J*_*2*_ = 7.0, *J*_*3*_ = 1.1 Hz, 2H), 4.98 (s, 2H, CH_2_), 3.97–3.84 (m, 1H, CH), 2.03–1.86 (m, 2H), 1.78–1.57 (m, 3H), 1.46–1.10 (m, 6H). ^13^C NMR (100 MHz, CDCl_3_) δ 166.26, 164.40, 147.71, 141.43, 136.92, 130.97, 129.21, 127.19, 126.87, 126.01, 124.84, 63.48, 47.97, 32.82, 25.52, 24.59. HRMS (ESI–TOF) *m/z*: 335.1371 [M + Na]^+^; calcd. for C_18_H_20_N_2_O_3_Na, 335.1372.

#### 4.2.5 2-(Cyclohexylamino)-2-oxoethyl 2-oxo-2*H*-chromene-3-carboxylate


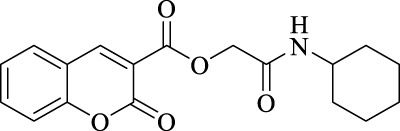


Depsipeptide **P5** (234 mg, 71%) as white solid. Rf = 0.44 (Hex/EtOAc 1:1 v/v). Mp = 158.1°C–159.0°C. UV (DCM, λ_max_ = 296 nm). IR ((KBr) 3,286, 2,927, 2,854, 1,762, 1708, 1,662, 1,616, 1,562, 1,450, 1,415, 1,365, 1,265, 1,207, 1,134, 1,045, 975, 921, 887, 798, 759 (cm^−1^). ^1^H NMR (400 MHz, DMSO) *δ* 8.86 (s, 1H), 7.97 (d, *J* = 7.7 Hz, 1H), 7.78 (t, *J* = 7.8 Hz, 1H), 7.45 (dd, *J* = 16.3, 8.1 Hz, 2H), 4.68 (s, 2H, CH_2_), 3.70–3.48 (m, 1H, CH), 1.83–1.62 (m, 4H), 1.55 (d, *J* = 12.6 Hz, 1H), 1.35–1.08 (m, 5H).^13^C NMR (100 MHz, DMSO) *δ* 165.65, 162.43, 156.79, 155.10, 150.15, 135.32, 130.94, 125.47, 118.27, 117.48, 116.71, 63.51, 47.91, 32.75, 25.62, 24.86. HRMS (ESI–TOF) *m/z*: 330.1317 [M + H]^+^; calcd. for C_18_H_20_NO_5_, 330.1341.

#### 4.2.6 2-(Cyclohexylamino)-2-oxoethyl quinoline-3-carboxylate


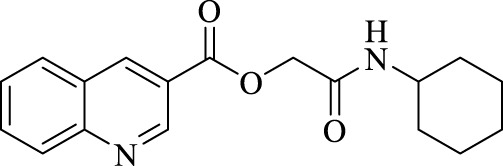


Depsipeptide **P6** (80 mg, 26%) as white solid. Rf = 0.16 (Hex/EtOAc 1:1 v/v). Mp = 201.8°C–204.7°C. UV (DCM, λ_max_ = 282 nm). IR ((KBr) 3,375, 3,089, 2,931, 2,854, 1,728, 1,654, 1,620, 1,600, 1,562, 1,496, 1,427, 1,377, 1,284, 1,269, 1,222, 1,199, 1,114, 972, 887, 786, 767, 744 (cm^−1^). ^1^H NMR (400 MHz, DMSO) *δ* 9.37 (d, *J* = 1.9 Hz, 1H), 9.07 (d, *J* = 1.5 Hz, 1H), 8.25 (d, *J* = 8.1 Hz, 1H), 8.14 (d, *J* = 8.5 Hz, 1H), 7.95 (t, *J* = 7.6 Hz, 1H), 7.75 (t, *J* = 7.5 Hz, 1H), 4.80 (s, 2H, CH_2_), 3.65–3.57 (m, 1H, CH), 1.82–1.64 (m, 4H), 1.56 (d, *J* = 12.7 Hz, 1H), 1.39–1.03 (m, 6H). ^13^C NMR (100 MHz, DMSO) *δ* 165.70, 164.86, 149.95, 149.72, 139.36, 132.84, 130.16, 129.30, 128.19, 126.93, 122.86, 63.66, 48.07, 32.77, 25.63, 25.01. HRMS (ESI–TOF) *m/z*: 313.1542 [M + H]^+^; calcd. for C_18_H_21_N_2_O_3_, 313.1552.

#### 4.2.7 *N*-benzyl-*N*-(2-(cyclohexylamino)-2-oxoethyl)benzamide


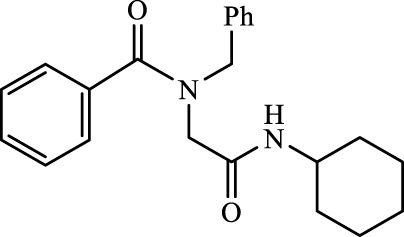


Peptoid **U1** (219.7 mg, 62%) as a white solid. Rf = 0.48 (Hex/EtOAc 1:1 v/v). Mp = 122.0°C–124.6°C. UV (DCM, λ_max_ = 227 nm). IR ((KBr) 3,277, 3,055, 2,826, 2,851, 1,631, 1,549, 1,446, 1,246, 1,149, 1,091, 993, 941, 698 (cm^−1^). ^1^H NMR (400 MHz, CDCl_3_) δ 7.29 (s, 2H), 7.27–7.07 (m, 7H), 7.01 (s, 1H), 6.18 (s, 1H), 4.47 (s, 2H), 3.86 (s, 2H), 3.59 (s, 1H), 1.69 (s, 2H), 1.52 (d, *J* = 11.1 Hz, 2H), 1.42 (d, *J* = 9.5 Hz, 1H), 1.19 (d, *J* = 10.7 Hz, 2H), 1.10–0.80 (m, 3H). ^13^C NMR (100 MHz, CDCl_3_) δ 172.88, 167.63, 135.94, 135.24, 130.13, 128.92, 128.63, 127.88, 127.16, 126.80, 54.05, 49.46, 48.16, 32.90, 25.48, 24.69. HRMS (ESI–TOF) *m/z*: 373.1895 [M + Na]^+^; calcd. for C_22_H_26_N_2_O_2_Na, 373.1892.

#### 4.2.8 *N*-benzyl-*N*-(2-(cyclohexylamino)-2-oxoethyl)cinnamamide


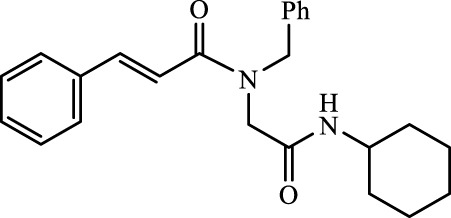


Peptoid **U2** (226 mg, 60%) as a white solid. Rf = 0.23 (Hex/EtOAc 2:1 v/v). Mp = 136.6°C–137.9°C. UV (DCM, λ_max_ = 287 nm). IR (KBr) 3,446, 3,270, 3,077, 3,028, 2,930, 2,852, 1,646, 1,600, 1,554, 1,496, 1,444, 1,209, 930, 765, 700 (cm^−1^). ^1^H NMR (400 MHz, CDCl_3_)) δ 7.61 (dd, *J*_*1*_ = 15.3, *J*_*2*_ = 5.3 Hz, 1H), 7.32–7.23 (m, 2H), 7.13 (dd, *J*_*1*_ = 16.7, *J*_*2*_ = 5.9 Hz, 7H), 7.03 (d, *J* = 7.3 Hz, 1H), 6.67 (d, *J* = 15.4 Hz, 1H), 6.47 (d, *J* = 15.3 Hz, 1H), 4.56 (s, 2H), 3.85 (s, 2H), 3.58–3.38 (m, 1H), 1.71–1.36 (ms, 5H), 1.14–0.64 (ms, 5H). ^13^C NMR (100 MHz, CDCl_3_) δ 168.07, 167.88, 144.51, 136.08, 134.82, 130.17, 130.06, 129.16, 129.05, 128.86, 128.77, 128.16, 128.08, 128.00, 126.71, 116.40, 52.50, 52.22, 51.48, 48.15, 32.87, 32.63, 25.52, 25.29, 24.69. HRMS (ESI–TOF) *m/z*: 377.2223 [M + H]^+^; calcd. for C_24_H_29_N_2_O_2_, 377.2229.

#### 4.2.9 (1*R*,5*S*)-*N*-benzyl-*N*-(2-(cyclohexylamino)-2-oxoethyl)-6,6-dimethylbicyclo[3.1.1]hept-2-ene-2-carboxamide


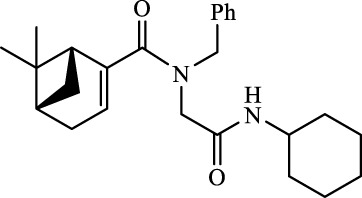


Peptoid **U3** (374.9 mg, 95%) as a white solid. Rf = 0.35 (Hex/EtOAc 3:1 v/v). Mp = 65.2°C–66.9°C. UV (DCM, λ_max_ = 227 nm). The spectroscopic data are in agreement with the published data (Concepción et al., 2020).

#### 4.2.10 *N*-benzyl-*N*-(2-(cyclohexylamino)-2-oxoethyl)isoquinoline-1-carboxamide


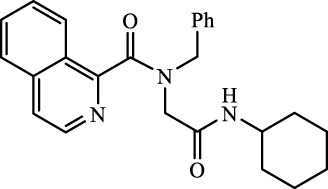


Peptoid **U4** (295.6 mg, 74%) as a white solid. Rf = 0.30 (Hex/EtOAc 1:1 v/v). Mp = 89.1°C–90.2°C. UV (DCM, λ_max_ = 233 nm). IR (KBr) 3,267, 3,057, 2,928, 2,853, 1,650, 1,559, 1,451, 1,300, 1,241, 1,183, 1,081, 978, 827, 745, 698 (cm^*−*1^). ^1^H NMR (400 MHz, CDCl_3_) δ 8.55 (d, *J* = 5.7 Hz, 1H), 8.42 (d, *J* = 5.7 Hz, 1H), 8.20 (dd, *J*_*1*_ = 16.7, *J*_*2*_ = 8.3 Hz, 2H), 7.88 (t, *J* = 9.0 Hz, 1H), 7.78–7.61 (m, 4H), 7.53 (d, *J* = 7.2 Hz, 2H), 7.47–7.22 (m, 6H), 4.89 (s, 2H), 4.43 (s, 1H), 4.16 (s, 1H), 3.91–3.82 (m, 1H), 3.75 (td, *J*_*1*_ = 14.2, *J*_*2*_ = 7.2 Hz, 1H), 3.67 (s, 2H), 2.02–1.56 (m, 8H), 1.56–1.17 (m, 7H). ^13^C NMR (100 MHz, CDCl_3_) δ 169.50, 168.24, 167.24, 167.14, 154.69, 154.03, 141.73, 140.21, 136.85, 136.44, 136.14, 135.42, 131.30, 130.92, 129.03, 128.87, 128.70, 128.67, 128.42, 128.29, 128.05, 127.93, 127.11, 126.98, 126.26, 125.98, 122.57, 121.74, 53.62, 52.71, 49.03, 48.38, 48.32, 48.10, 32.96, 32.80, 25.60, 25.54, 24.71, 24.62. HRMS (ESI–TOF) *m/z*: 424.2008 [M + Na]^+^; calcd. for C_25_H_27_N_3_O_2_Na, 424.2001.

#### 4.2.11 *N*-benzyl-*N*-(2-(cyclohexylamino)-2-oxoethyl)-2-oxo-2*H*-chromene-3-carboxamide


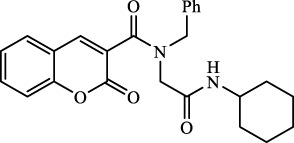


Peptoid **U5** (238.3 mg, 57%) as a white solid. Rf = 0.28 (Hex/EtOAc 1:1 v/v). Mp = 166.1°C–168.0°C. UV (DCM, λ_max_ = 295 nm). IR ((KBr) 3,427, 3,300, 3,065, 2,931, 2,850, 1,721, 1,660, 1,634, 1,606, 1,539, 1,452, 1,237, 975, 760, 692 (cm^−1^). ^1^H NMR (400 MHz, CDCl_3_) δ 7.90 (s, 1H), 7.58–7.47 (m, 2H), 7.39–7.19 (m, 6H), 7.06 (d, *J* = 6.8 Hz, 1H), 4.69 (s, 1H), 4.43 (s, 2H), 3.77–3.57 (m, 2H), 1.95–0.97 (m, 10H). ^13^C NMR (100 MHz, CDCl_3_) δ 166.15, 165.67, 159.58, 153.89, 143.33, 135.09, 133.42, 129.09, 128.85, 128.72, 128.38, 127.22, 125.42, 124.59, 118.19, 117.00, 53.45, 48.63, 48.22, 32.89, 25.46, 25.00. HRMS (ESI–TOF) m/z: 441.1795 [M + Na]^+^; calcd. for C_24_H_28_N_2_O_2_Na: 441.1790.

#### 4.2.12 *N*-benzyl-*N*-(2-(cyclohexylamino)-2-oxoethyl)quinoline-3-carboxamide


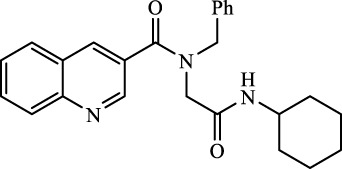


Peptoid **U6** (260.6 mg, 65%) as a white solid. Rf = 0.20 (Hex/EtOAc 1:1 v/v). Mp = 149.7°C–152.0°C. UV (DCM, λ_max_ = 233 nm). IR ((KBr) 3,441, 3,306, 3,062, 2,931, 2,850, 1,657, 1,647, 1,546, 1,432, 1,227 (cm^−1^). ^1^H NMR (400 MHz, CDCl_3_) δ 8.89 (s, 1H), 8.17 (s, 1H), 7.97 (d, *J* = 8.3 Hz, 1H), 7.67–7.62 (m, 2H), 7.45 (t, *J* = 7.4 Hz, 1H), 7.23–7.19 (m, 4H), 7.07 (s, 1H), 6.11 (s, 1H), 4.61 (s, 2H), 3.98 (s, 2H), 3.67 (s, 1H), 1.82–1.77 (m, 2H), 1.55–1.47 (m, 3H), 1.22–1.04 (m, 2H). ^13^C NMR (100 MHz, CDCl_3_) δ 170.44, 167.09, 148.42, 148.07, 135.57, 134.96, 134.8, 130.75, 129.41, 129.06, 128.37, 128.08, 127.56, 126.97, 126.77, 54.26, 49.42, 48.38, 32.95, 25.43, 24.73. HRMS (ESI–TOF) *m/z*: 401.2109 [M]^+^; calcd. for C_25_H_27_N_3_O_2_, 401.2103.

#### 4.2.13 *N*-benzyl-*N*-(2-(dodecylamino)-2-oxoethyl)-2-oxo-2*H*-chromene-3-carboxamide


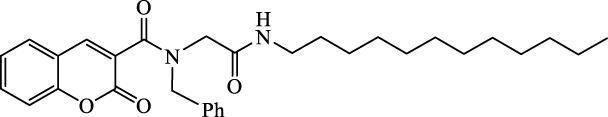


Peptoid **U7** (260.6 mg, 65%) as a white solid. Rf = 0.38 (Hex/EtOAc 1:1 v/v). Mp = 119.8°C–122.3°C. UV (DCM, λ_max_ = 297 nm). IR (KBr) 3,280, 3,080, 2,917, 2,849, 1736, 1,637, 1,545, 1,434, 1,233, 1,139, 964, 760, 710 (cm^−1^). ^1^H NMR (400 MHz, CDCl_3_) δ 7.99 (s, 1H), 7.71–7.61 (m, 1H), 7.57 (dd, *J*_*1*_ = 7.8, *J*_*2*_ = 1.3 Hz, 1H), 7.48–7.25 (m, 7H), 7.15 (d, *J* = 6.6 Hz, 2H), 4.53 (s, 2H), 3.34 (dd, *J*_*1*_ = 13.0, *J*_*2*_ = 7.0 Hz, 2H), 1.69–1.57 (m, 2H), 1.42–1.17 (m, 19H), 0.94–0.84 (m, 3H). ^13^C NMR (100 MHz, CDCl_3_) δ 167.13, 165.72, 159.93, 153.90, 143.29, 135.10, 133.41, 129.10, 128.84, 128.71, 128.40, 127.20, 125.41, 124.59, 118.19, 117.02, 53.50, 48.14, 39.80, 31.92, 29.66, 29.62, 29.58, 29.36, 29.26, 29.20, 26.92, 22.69, 14.12. HRMS (ESI–TOF) *m/z*: 527.2930 [M + Na]^+^; calcd. for C_31_H_40_N_2_O_4_Na: 527.2886.

### 4.3 Theoretical calculations

All the computational calculations were performed with the Gaussian 09 package (Frisch et al., 2009). The DFT method was used for the ground state optimization, while for the excited state optimization, TD-DFT was employed. The hybrid functional CAM-B3LYP (Coulomb-attenuating method–Becke3–Lee–Yang–Parr hybrid functional) and the 6–311g(d,p) basis set were used for all atoms (Yanai et al., 2004). The polarizable continuum model (PCM) was used to optimize the ground and excited state geometries (Cancès et al., 1997; Mennucci et al., 1997). The excitation energies, oscillator strengths, and orbital contributions for the lowest 10 singlet–singlet transitions at the optimized geometry in the ground state were obtained by TD-DFT calculations using the same basis set as for the geometry minimization. The lowest singlet excited state (S1) was relaxed using TD-DFT to get the optimized excited state geometry. Emissions were obtained by calculating vertical excitations of the excited state geometry at the ground state. A non-equilibrium state of solvation was assumed (Cammi et al., 2005). The solvents used were THF, DCM, EtOH, MeCN, and Hex, which were consistent with experimental data.

### 4.4 Cytotoxicity assay

The human cell lines (HEK293 and HeLa) used in the cell viability assays were purchased from American Type Culture Collection (Manassas, VA, USA). Both cell lines were cultured in Dulbecco’s modified Eagle medium supplemented with 10% fetal bovine serum, 100 units/mL penicillin, and 100 mg/mL streptomycin. They were kept at 37°C in a humidified atmosphere containing 5% CO_2_. For 6-, 12-, 24-, and 48-hour conditions, a 100 μL aliquot of adherent cells was used to seed 96-well cell culture plates at 20,000 cells/well and allowed to adhere for 16 h prior to the addition of the compounds. While for 72- hour condition, a 100 μL aliquot of adherent cells was used to seed 96-well cell culture plates at 15,000 cells/well and allowed to adhere for 16 h prior to the addition of the compounds.

Cell viability assay was performed in HEK293 and HeLa cells using the (2-(2-methoxy-4-nitrophenyl)-3-(4-nitrophenyl)-5-(2,4-disulfophenyl)-2H-tetrazolium, monosodium salt) (CCK-8) method (Sigma-Aldrich, Merck KGaA, Darmstadt, Germany). The compounds were assayed under increasing concentrations of 0.27, 132, and 2,650 µM. The cell viability was detected by the absorbance measure at 450 nm in Synergy H1 microplate reader (BioTek Instruments, USA). All experiments were conducted in triplicate.

## Data Availability

The datasets presented in this study can be found in the online repository PubChem. The accession numbers can be found in the following list: P1 (SID: 483124285, CID: 2517305), P2 (SID: 483124286, CID: 4855926), P3 (SID: 483124287, CID: 168433793), P4 (SID: 483124288, CID: 71823459), P5 (SID: 483124289, CID: 4376332), P6 (SID: 483124290, CID: 168433794), U1 (SID: 483124291, CID: 71663623), U2 (SID: 483124292, CID: 168433795), U3 (SID: 483124293, CID: 168433796), U4 (SID: 483124294, CID: 168433797), U5 (SID: 483124295, CID: 168433798), U6 (SID: 483124296, CID: 168433799), U7 (SID: 483124297, CID: 168433800).
