# Supplementary material for: Multicomponent synthesis and photophysical study of novel α,β-unsaturated carbonyl depsipeptides and peptoids
Source: Front Chem. 2023 Aug 17;11:1245941. doi: 10.3389/fchem.2023.1245941 (PMC10471130; doi:10.3389/fchem.2023.1245941)
Supplement: Supplementary file 1 [file DataSheet1.docx]

**Supplementary material**

**Multicomponent Synthesis and photophysical study of novel α,β-unsaturated carbonyl peptidomimetics**

Ricelia González^1^, Juliana Murillo-López^2^, Walter Rabanal-León^2^, Luis Prent-Peñaloza^3^, Odette Concepción^2^, Pedro Olivares^4^, Yorley Duarte^4^, Alexander F. de la Torre^2^, Margarita Gutiérrez^5*^, Julio Caballero^6*^

^1^ Doctorado en Ciencias Mención I + D de Productos Bioactivos, Instituto de Química de Recursos Naturales, Laboratorio de Síntesis Orgánica, Universidad de Talca, Talca, Chile

^2^ Departamento de Química Orgánica. Facultad de Ciencias Químicas, Universidad de Concepción, Concepción, Chile

^3^ Departamento de Ciencias Químicas, Facultad de Ciencias Exactas, Universidad Andrés Bello, Viña del Mar, Chile

^4^ Center for Bioinformatics and Integrative Biology, Facultad de Ciencias de la Vida, Universidad Andrés Bello, Santiago 8370035, Chile

^5^ Laboratorio de Síntesis Orgánica, Instituto de Química de Recursos Naturales, Universidad de Talca, Talca, Chile ([mgutierrez@utalca.cl](mailto:mgutierrez@utalca.cl))

^6^ Departamento de Bioinformática, Facultad de Ingeniería, Centro de Bioinformática, Simulación y Modelado (CBSM), Universidad de Talca, Talca, Chile ([jcaballero@utalca.cl](mailto:jcaballero@utalca.cl))

**Index**

**Section S1** UV results: Concentrations and dilutions performed for UV spectra measurements. Emission test, results

**Section S2** Figures and spectra: 400 MHz ^1^H NMR and 100 MHz ^13^C NMR spectra; HRMS spectra; FT-IR spectra; UV spectra; PL spectra.

**Section S3** Fluorescence and Photostability test (Spectrophotometric and HPLC results).

**S1.** UV results.

**Table S1.1.** Concentrations and dilutions performed for UV spectra measurements for P-3CR products in DCM.

| Compound | mass (mg) | M (g/mol) | mmols | Stock Conc. (mM) | Dilution Factor | UV Conc. (mM) | λ _max_  (nm) | *ξ* _max_ (L/mol·cm) |
| --- | --- | --- | --- | --- | --- | --- | --- | --- |
| P1 | 2.8 | 261.32 | 4.59E-03 | 1.071 | 10 | 0.1071 | 232 | 6915 |
| P2 | 0.7 | 287.35 | 2.43E-03 | 0.243 | 10 | 0.0243 | 281 | 26955 |
| P3 | 2.2 | 305.41 | 7.20E-03 | 0.720 | 10 | 0.0720 | 237 | 11083 |
| P4 | 2.2 | 312.36 | 7.04E-03 | 0.704 | 40 | 0.0176 | 229 | 26704 |
| P5 | 2.1 | 329.35 | 6.37E-03 | 0.637 | 10 | 0.0637 | 296 | 11789 |
| P6 | 2.0 | 312.36 | 6.40E-03 | 0.640 | 10 | 0.0640 | 286 | 6046 |

**Table S1.2.** Concentrations and dilutions performed for UV spectra measurements for P-3CR products in EtOH.

| Compound | mass (mg) | | M (g/mol) | | mmols | | Stock Conc. (mM) | Dilution Factor | UV Conc. (mM) | λ _max_  (nm) | *ξ* _max_ (L/mol·cm) |
| --- | --- | --- | --- | --- | --- | --- | --- | --- | --- | --- | --- |
| P1 | | 1.2 | | 261.32 | | 4.59E-03 | 0.459 | 10 | 0.0459 | 227 | 17342 |
| P2 | 1.5 | | 287.35 | | 5.22E-03 | | 0.522 | 10 | 0.0522 | 278 | 10344 |
| P3 | 1.7 | | 305.41 | | 5.56E-03 | | 0.556 | 10 | 0.0556 | 233 | 4639 |
| P4 | 0.9 | | 312.36 | | 2.88E-03 | | 0.288 | 40 | 0.0072 | 220 | 57361 |
| P5 | 1.0 | | 329.35 | | 3.03E-03 | | 0.303 | 10 | 0.0303 | 296 | 12772 |
| P6 | | 2.2 | | 312.36 | | 7.04E-03 | 0.704 | 20 | 0.0352 | 281 | 10113 |

**Table S1.3.** Concentrations and dilutions performed for UV spectra measurements for P-3CR products in Hex.

| Compound | mass (mg) | | M (g/mol) | | mmols | | Stock Conc. (mM) | | Dilution Factor | | UV Conc. (mM) | | λ _max_  (nm) | | *ξ* _max_ (L/mol·cm) | | |
| --- | --- | --- | --- | --- | --- | --- | --- | --- | --- | --- | --- | --- | --- | --- | --- | --- | --- |
| P1 | | 2.8 | | 261.32 | | 4.59E-03 | | 1.071 | | 20 | | 0.0535 | | 198 | | 15794 |  |
| P2 | 1.2 | | 287.35 | | 4.17E-03 | | 0.417 | | 40 | | 0.0104 | | 273 | | 44615 | | |
| P3 | 2.4 | | 305.41 | | 7.85E-03 | | 0.785 | | 10 | | 0.0785 | | 194 | | 10101 | | |
| P4 | 0.9 | | 312.36 | | 2.88E-03 | | 0.288 | | 40 | | 0.0072 | | 221 | | 36527 | | |
| P5 | 2.3 | | 329.35 | | 6.98E-03 | | 0.698 | | 5 | | 0.1396 | | 290 | | 1948 | | |
| P6 | | - | |  | |  | |  | |  | |  | |  | |  | |

**Table S1.4.** Concentrations and dilutions performed for UV spectra measurements for P-3CR products in THF.

| Compound | mass (mg) | M (g/mol) | mmols | Stock Conc. (mM) | Dilution Factor | | UV Conc. (mM) | λ _max_  (nm) | | *ξ* _max_ (L/mol·cm) | |
| --- | --- | --- | --- | --- | --- | --- | --- | --- | --- | --- | --- |
| P1 | 2.0 | 261.32 | 7.65E-03 | 0.765 | | 20 | 0.0382 | | 228 | 13612 |  |
| P2 | 1.1 | 287.35 | 3.82E-03 | 0.382 | 20 | | 0.0191 | 276 | | 22251 | |
| P3 | 2.4 | 305.41 | 7.85E-03 | 0.785 | 10 | | 0.0785 | 231 | | 9783 | |
| P4 | 1.1 | 312.36 | 3.52E-03 | 0.352 | 40 | | 0.0088 | 220 | | 57840 | |
| P5 | 1.4 | 329.35 | 4.25E-03 | 0.425 | 20 | | 0.0212 | 236 | | 12500 | |
| P6 | 3.5 | 312.36 | 1.12 E-02 | 1.120 | 10 | | 0.1120 | 281 | | 5169 | |

**Table S1.5.** Concentrations and dilutions performed for UV spectra measurements for P-3CR products in MeCN.

| Compound | mass (mg) | M (g/mol) | mmols | Stock Conc. (mM) | Dilution Factor | UV Conc. (mM) | | λ _max_  (nm) | ξ _max_ (L/mol·cm) |
| --- | --- | --- | --- | --- | --- | --- | --- | --- | --- |
| P1 | 2.3 | 261.32 | 8.80E-03 | 0.880 | 40 | 0.0220 | 194 | | 41090 |
| P2 | 1.0 | 287.35 | 3.48E-03 | 0.348 | 20 | 0.0174 | | 272 | 48390 |
| P3 | 1.8 | 305.41 | 5.89E-03 | 0.589 | 20 | 0.0294 | | 192 | 16020 |
| P4 | 1.3 | 312.36 | 4.16E-03 | 0.416 | 40 | 0.0104 | | 221 | 24615 |
| P5 | 1.5 | 329.35 | 4.55E-03 | 0.455 | 10 | 0.0455 | | 292 | 16373 |
| P6 | 2.7 | 312.36 | 8.64E-03 | 0.864 | 10 | 0.0864 | | 281 | 4652 |

**Table S1.6.** Concentrations and dilutions performed for UV spectra measurements for U-4CR products in DCM.

| Compound | mass (mg) | M (g/mol) | mmols | Stock Conc. (mM) | Dilution Factor | UV Conc. (mM) | λ _max_  (nm) | *ξ* _max_ (L/mol·cm) |
| --- | --- | --- | --- | --- | --- | --- | --- | --- |
| U1 | 0.9 | 350.46 | 2.56E-03 | 0.256 | 5 | 0.0512 | 227 | 7407 |
| U2 | 1.0 | 376.50 | 2.65E-03 | 0.265 | 20 | 0.0132 | 287 | 32500 |
| U3 | 1.7 | 394.55 | 4.30E-03 | 0.430 | 5 | 0.0860 | 228 | 7755 |
| U4 | 2.4 | 401.51 | 2.24E-03 | 0.224 | 10 | 0.0224 | 233 | 38392 |
| U5 | 2.5 | 418.49 | 5.97E-03 | 0.597 | 10 | 0.0597 | 293 | 12747 |
| U6 | 1.7 | 401.51 | 4.23E-03 | 0.423 | 10 | 0.0423 | 227 | 17635 |
| U7 | 0.9 | 504.67 | 1.78E-03 | 0.178 | 10 | 0.0178 | 295 | 28764 |

**Table S1.7.** Concentrations and dilutions performed for UV spectra measurements for U-4CR products in EtOH.

| Compound | mass (mg) | M (g/mol) | mmols | Stock Conc. (mM) | Dilution Factor | UV Conc. (mM) | λ _max_  (nm) | *ξ* _max_ (L/mol·cm) |
| --- | --- | --- | --- | --- | --- | --- | --- | --- |
| U1 | 0.9 | 350.46 | 2.56E-03 | 0.256 | 20 | 0.0128 | 203 | 41171 |
| U2 | 1.6 | 376.50 | 4.24E-03 | 0.424 | 20 | 0.0212 | 204 | 23018 |
| U3 | 1.6 | 394.55 | 4.05E-03 | 0.405 | 20 | 0.0202 | 202 | 20099 |
| U4 | 1.0 | 401.51 | 2.49E-03 | 0.249 | 10 | 0.0249 | 228 | 34859 |
| U5 | 2.4 | 418.49 | 5.73E-03 | 0.573 | 10 | 0.0573 | 290 | 11134 |
| U6 | 1.3 | 401.51 | 3.23E-03 | 0.323 | 40 | 0.0080 | 219 | 66790 |
| U7 | 1.1 | 504.67 | 2.17E-03 | 0.217 | 20 | 0.0108 | 203 | 46574 |

**Table S1.8.** Concentrations and dilutions performed for UV spectra measurements for U-4CR products in Hex.

| Compound | mass (mg) | M (g/mol) | mmols | Stock Conc. (mM) | Dilution Factor | UV Conc. (mM) | λ _max_  (nm) | *ξ* _max_ (L/mol·cm) |
| --- | --- | --- | --- | --- | --- | --- | --- | --- |
| U1 | 0.8 | 350.46 | 2.28E-03 | 0.228 | 10.00 | 0.0228 | 193 | 25047 |
| U2 | 1.3 | 376.50 | 3.45E-03 | 0.345 | 10.00 | 0.0345 | 193 | 20550 |
| U3 | 1.1 | 394.55 | 2.78E-03 | 0.278 | 10.00 | 0.0278 | 194 | 24172 |
| U4 | 2.5 | 401.51 | 6.22E-03 | 0.622 | 5.00 | 0.1244 | 233 | 2137 |
| U5 | 1.1 | 418.49 | 2.62E-03 | 0.262 | 5.00 | 0.0524 | 191 | 9541 |
| U6 | 1.2 | 401.51 | 2.98E-03 | 0.298 | 20.00 | 0.0149 | 219 | 26778 |
| U7 | 1.1 | 504.67 | 2.17E-03 | 0.217 | 10.00 | 0.0217 | 193 | 14792 |

**Table S1.9.** Concentrations and dilutions performed for UV spectra measurements for U-4CR products in THF.

| Compound | mass (mg) | M (g/mol) | mmols | Stock Conc. (mM) | Dilution Factor | UV Conc. (mM) | λ _max_  (nm) | *ξ* _max_ (L/mol·cm) |
| --- | --- | --- | --- | --- | --- | --- | --- | --- |
| U1 | 1.0 | 350.46 | 2.85E-03 | 0.285 | 10 | 0.0285 | 213 | 18175 |
| U2 | 0.9 | 376.50 | 2.39E-03 | 0.239 | 10 | 0.0239 | 212 | 33723 |
| U3 | 1.5 | 394.55 | 3.80E-03 | 0.380 | 10 | 0.0380 | 214 | 19131 |
| U5 | 2.6 | 418.49 | 6.21E-03 | 0.621 | 10 | 0.0621 | 294 | 11594 |
| U5 | 2.6 | 418.49 | 6.21E-03 | 0.621 | 10 | 0.0621 | 294 | 11594 |
| U6 | 1.4 | 401.51 | 3.48E-03 | 0.348 | 40 | 0.0087 | 220 | 68160 |
| U7 | 1.2 | 504.67 | 2.37E-03 | 0.237 | 10 | 0.0237 | 212 | 33248 |

**Table S1.10.** Concentrations and dilutions performed for UV spectra measurements for U-4CR products in MeCN.

| Compound | mass (mg) | M (g/mol) | mmols | Stock Conc. (mM) | Dilution Factor | UV Conc. (mM) | λ _max_  (nm) | *ξ* _max_ (L/mol·cm) |
| --- | --- | --- | --- | --- | --- | --- | --- | --- |
| U1 | 1.3 | 350.46 | 3.70E-03 | 0.370 | 40 | 0.0092 | 193 | 59115 |
| U2 | 1.4 | 376.50 | 3.71E-03 | 0.371 | 20 | 0.0185 | 191 | 50432 |
| U3 | 2.0 | 394.55 | 5.06E-03 | 0.506 | 40 | 0.0126 | 193 | 31825 |
| U4 | 1.5 | 401.51 | 3.73E-03 | 0.373 | 40 | 0.0093 | 191 | 84594 |
| U5 | 2.9 | 418.49 | 6.92E-03 | 0.692 | 10 | 0.0692 | 286 | 9826 |
| U6 | 1.4 | 401.51 | 3.48E-03 | 0.348 | 20 | 0.0174 | 218 | 45459 |
| U7 | 0.7 | 504.67 | 1.38E-03 | 0.138 | 20 | 0.0069 | 202 | 81449 |

**Table S1.11:** Emission study**.**

| **Compound** | **PL Conc. (mM)** | **PL λ _max_ (nm)** | **fluorescence intensity (a.u.)** | **Stokes shift (nm)** | ***Φ*** |
| --- | --- | --- | --- | --- | --- |
| P2  (DCM) | 0.0104 | 317; 339; 568 | 29; 26; 154 | 39; 61; 290 | - |
|  | 0.0191 | 317; 337; 568 | 26; 27; 82 | 39; 59; 290 | - |
|  | 0.0243 | 337; 570 | 30; 13 | 59; 292 | - |
| P2  (EtOH) | 0.0104 | 314; 570 | 69; 59 | 36; 292 | - |
|  | 0.0191 | 314; 570 | 69; 59 | 36; 292 | - |
|  | 0.0243 | 314, 569 | 47; 22 | 36; 291 | - |
| P5  (DCM) | 0.0075 | 312; 568 | 53; 404 | 16; 272 | - |
|  | 0.0151 | 312; 568 | 47; 390 | 16; 272 | - |
|  | 0.0303 | 312; 568 | 47; 389 | 16; 272 | - |
| P5  (EtOH) | 0.0075 | 311; 570 | 126; 231 | 15; 274 | - |
|  | 0.0151 | 311; 567 | 122; 167 | 15; 271 | - |
|  | 0.0303 | 311; 567 | 126; 167 | 15; 271 | - |
| U5  (DCM) | 0.0059 | 400; 593 | 39; 180 | 107; 300 | - |
|  | 0.0119 | 404; 593 | 65; 143 | 111; 300 | - |
|  | 0.0238 | 400; 593 | 102; 90 | 107; 300 | - |
| U6  (DCM) | 0.0009 | 478 | 208 | 245 | - |
|  | 0.0019 | 478 | 133 | 245 | - |
|  | 0.0038 | 482 | 22 | 249 | - |
| U6  (EtOH) | 0.0007 | 476 | 129 | 247 | 0,005 |
|  | 0.0014 | 478 | 57 | 249 | - |
|  | 0.0028 | 480 | 19 | 251 | - |

the indirect determination of quantum yield (Φ) was performed using fluorescein in EtOH as reference, at the λ _exc_ of the sample (220 nm). To know *Φ* under these conditions for fluorescein, in turn, it was determined knowing quantum yield fluorescein in EtOH at 490 nm (*Φ= 0,91*). (*10.1562/0031-8655(2002)0750327FQYATR2.0.CO2).*

**S2.** Spectra.

***** methanol added to favor the dissolution of the compound

**Figure S2.1.** (Top) 400 MHz ^1^H NMR and (bottom) 100 MHz ^13^C NMR spectra in CDCl_3_ of **P1**.


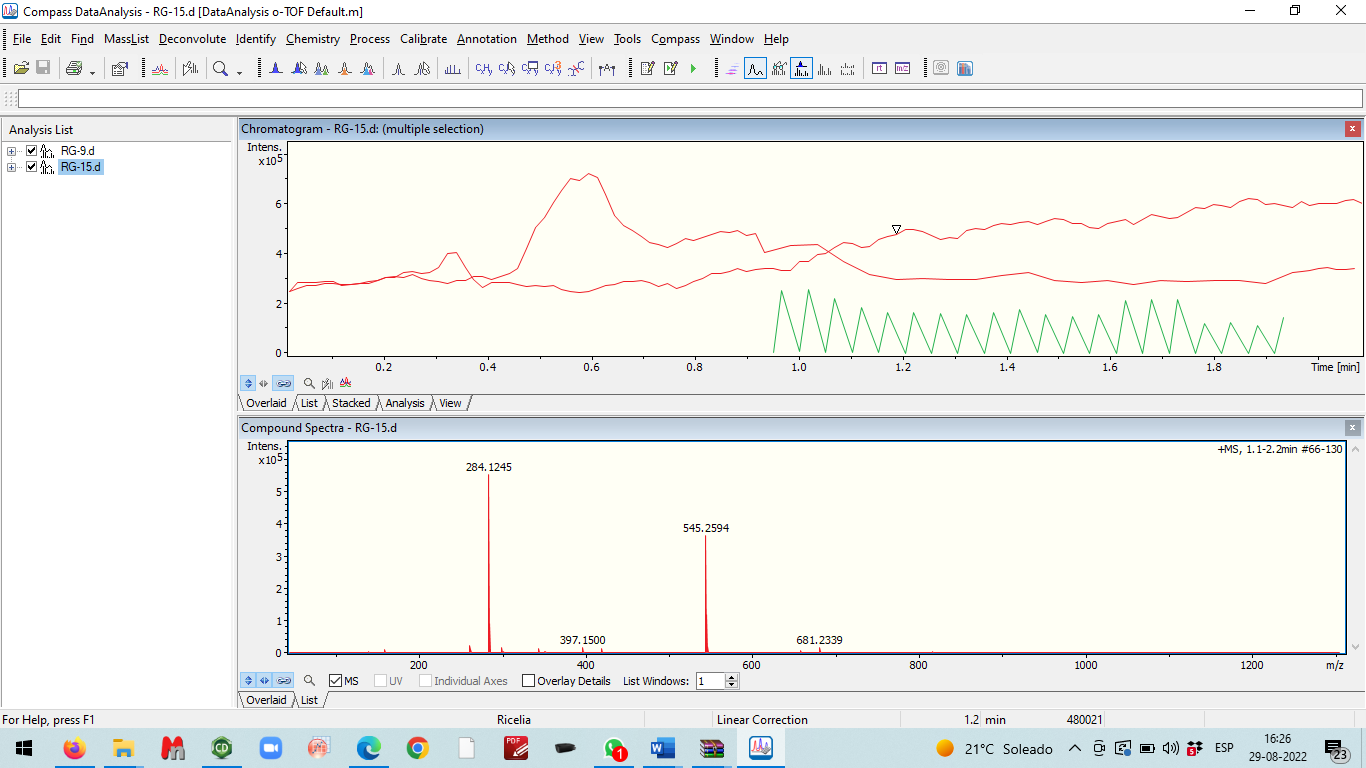


**Figure S2.2.** HRMS spectra of **P1**.


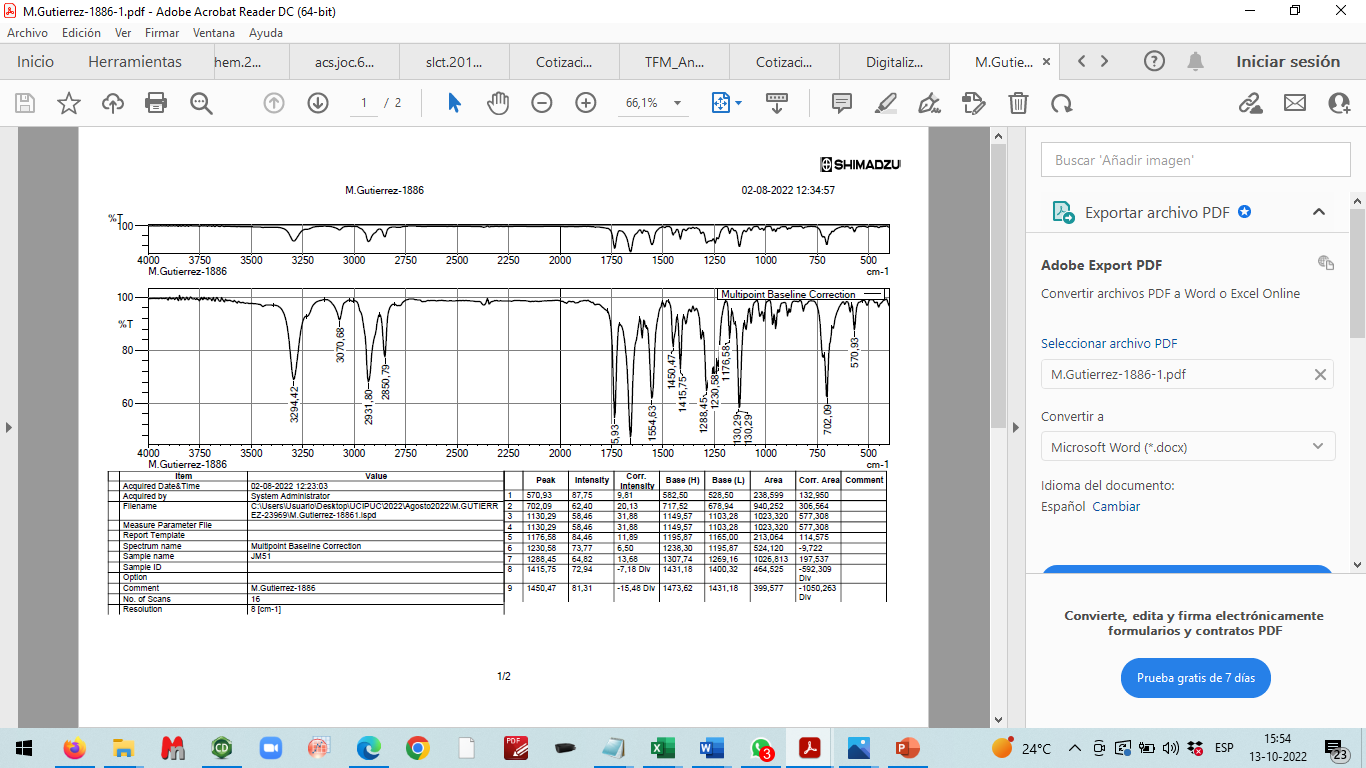


**Figure S2.3.**  FT-IR spectra of **P1**.


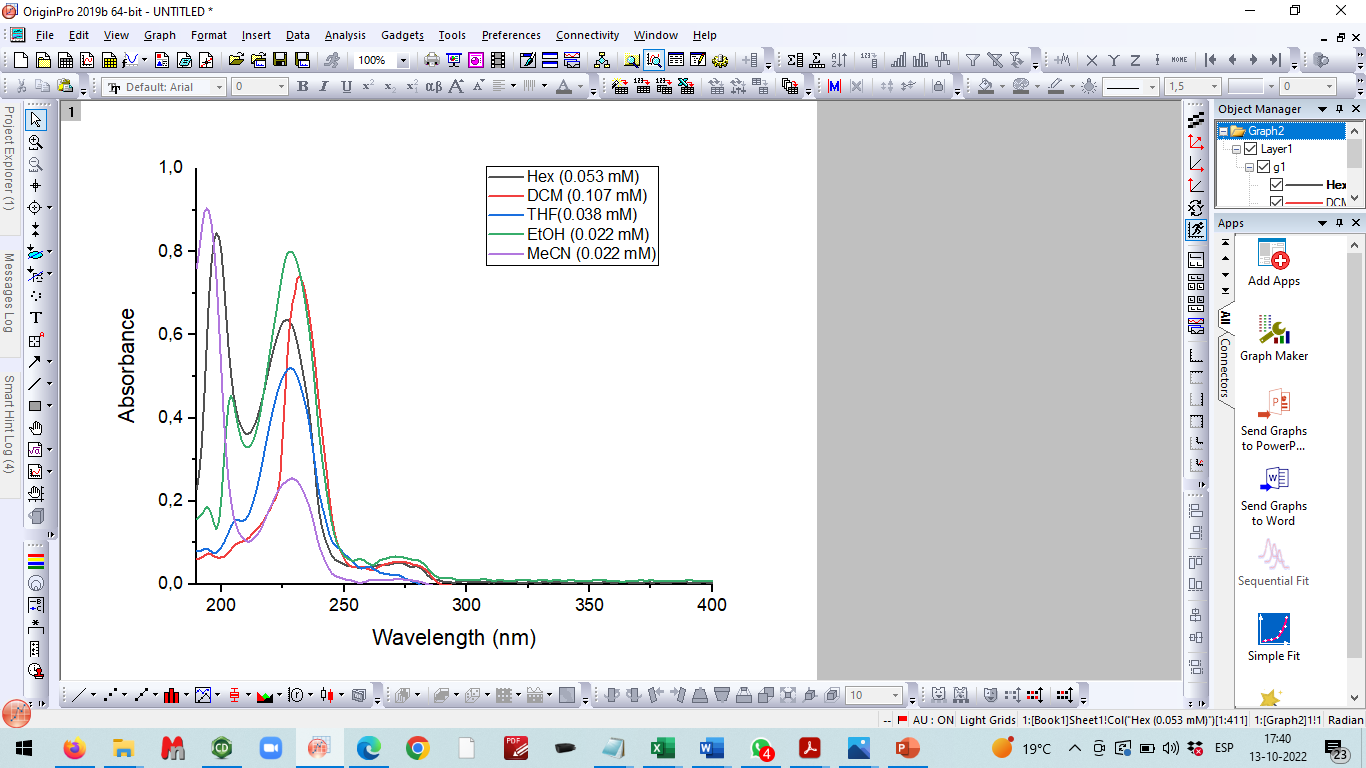


**Figure S2.4.**  UV spectra and solvents comparison of **P1**.

**
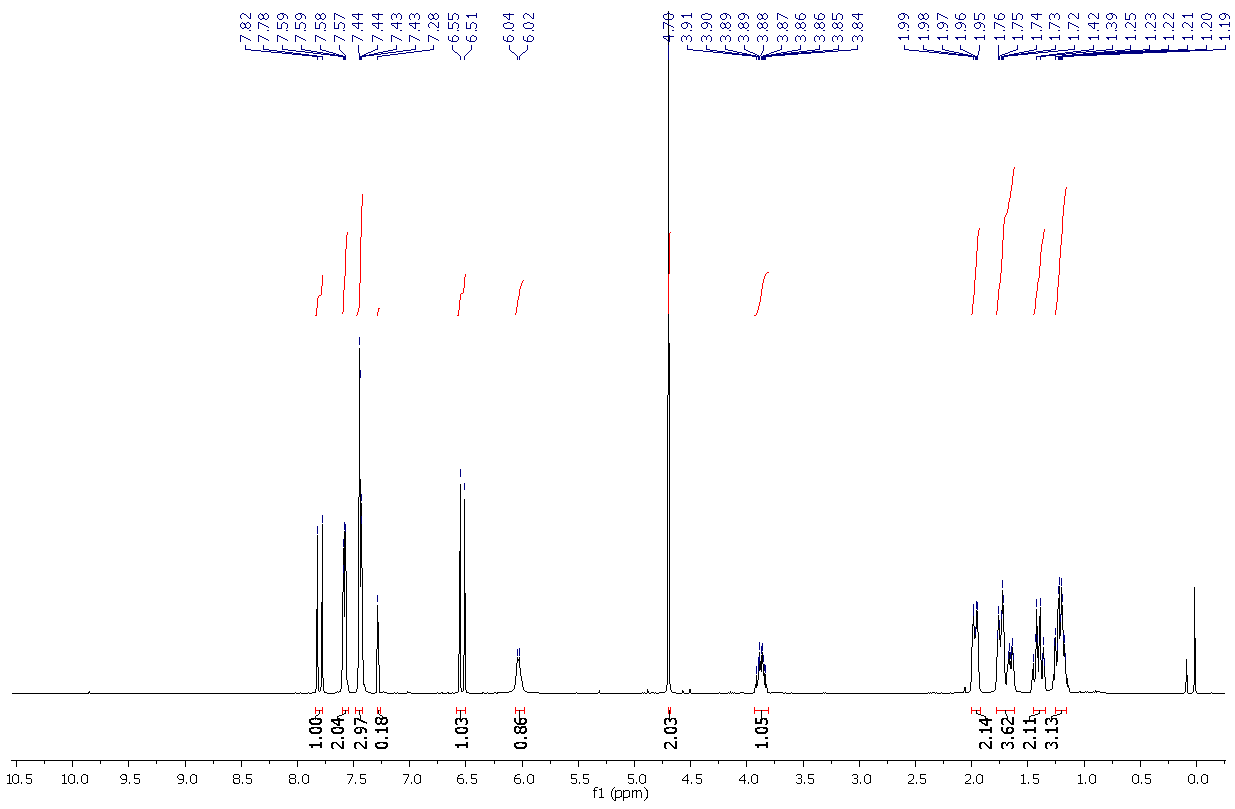
**

**
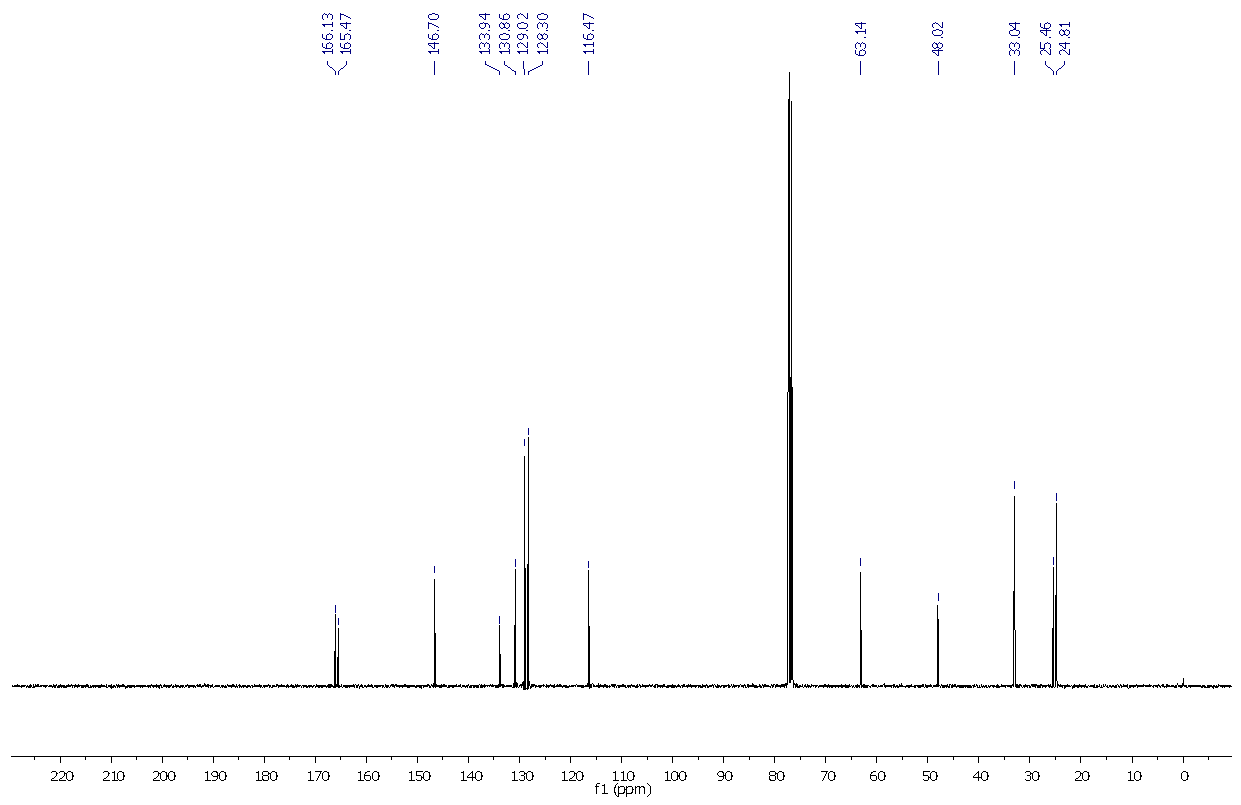
**

**Figure S2.5.** (Top) 400 MHz ^1^H NMR and (bottom) 100 MHz ^13^C NMR spectra in CDCl_3_ of **P2**.


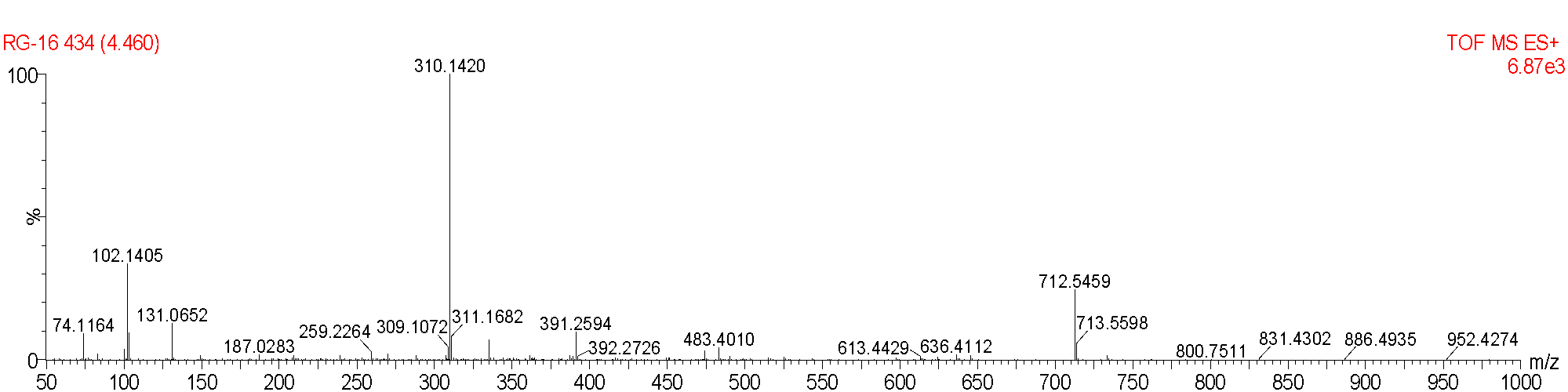


**Figure S2.6.** HRMS spectra of **P2**.


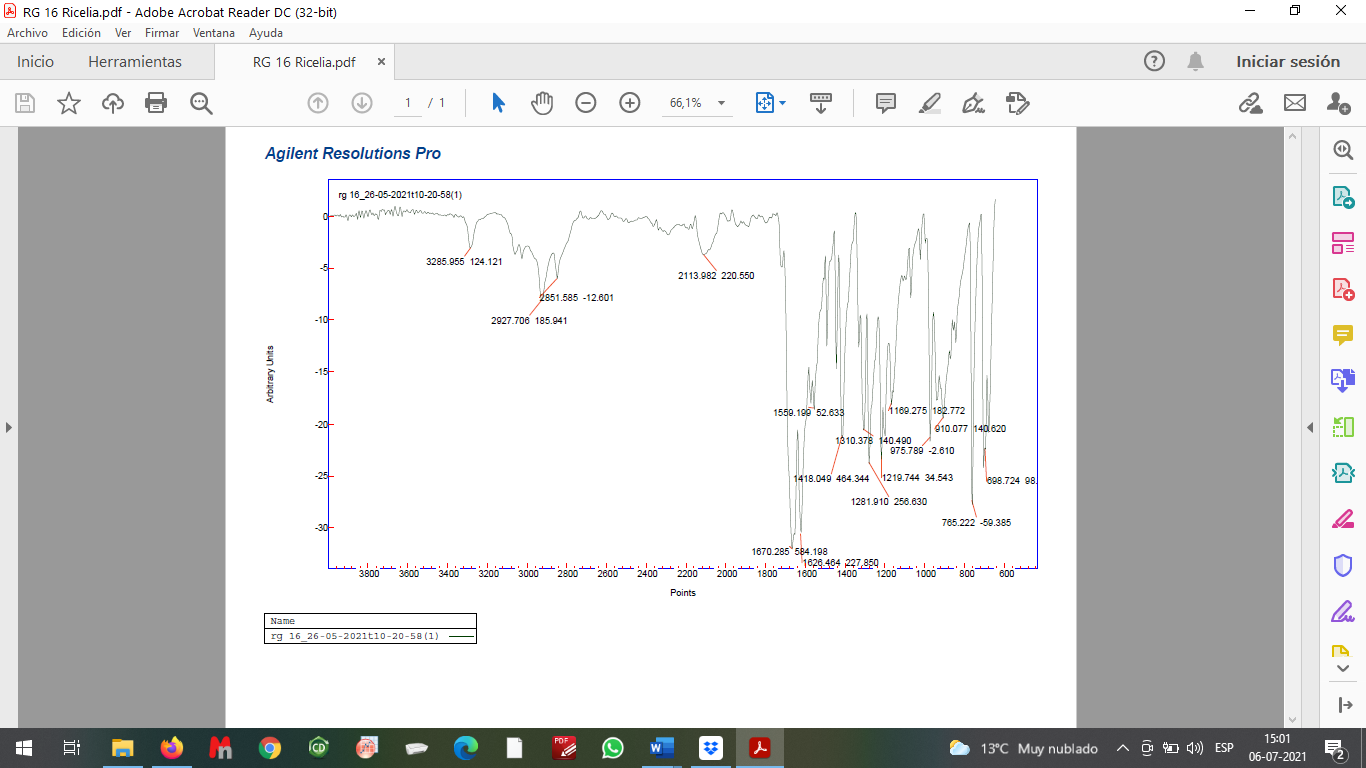


**Figure S2.7.** FT-IR spectra of **P2**.


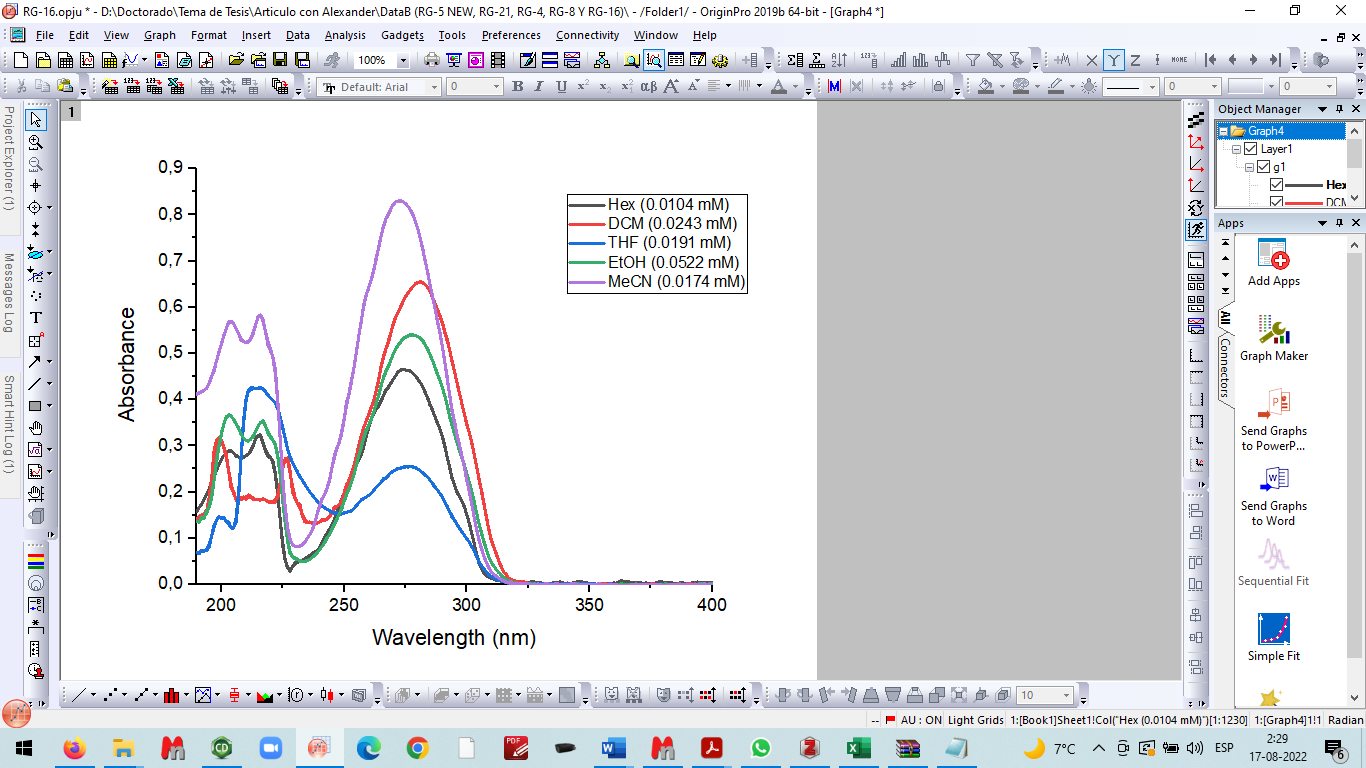


**Figure S2.8.** UV spectra and solvents comparison of **P2**.

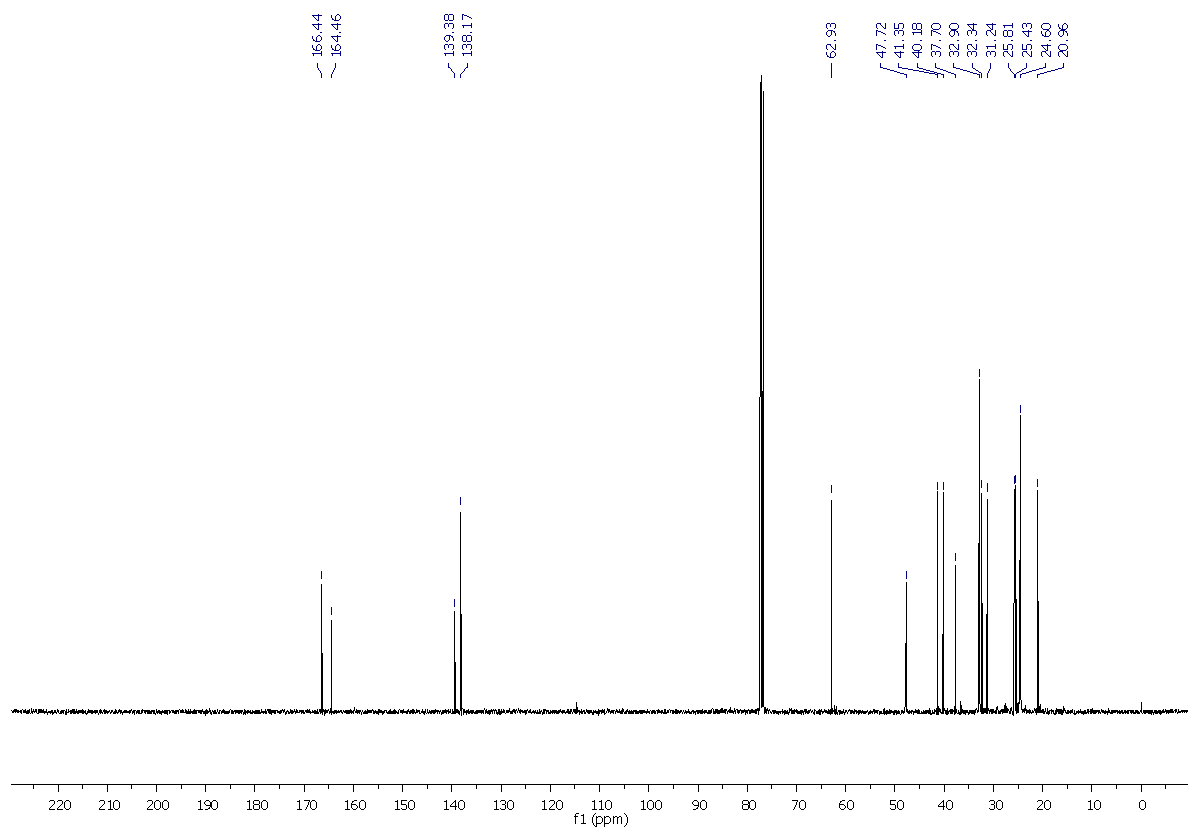


**Figure S2.9.** (Top) 400 MHz ^1^H NMR and (bottom) 100 MHz ^13^C NMR spectra in CDCl_3_ of **P3**.


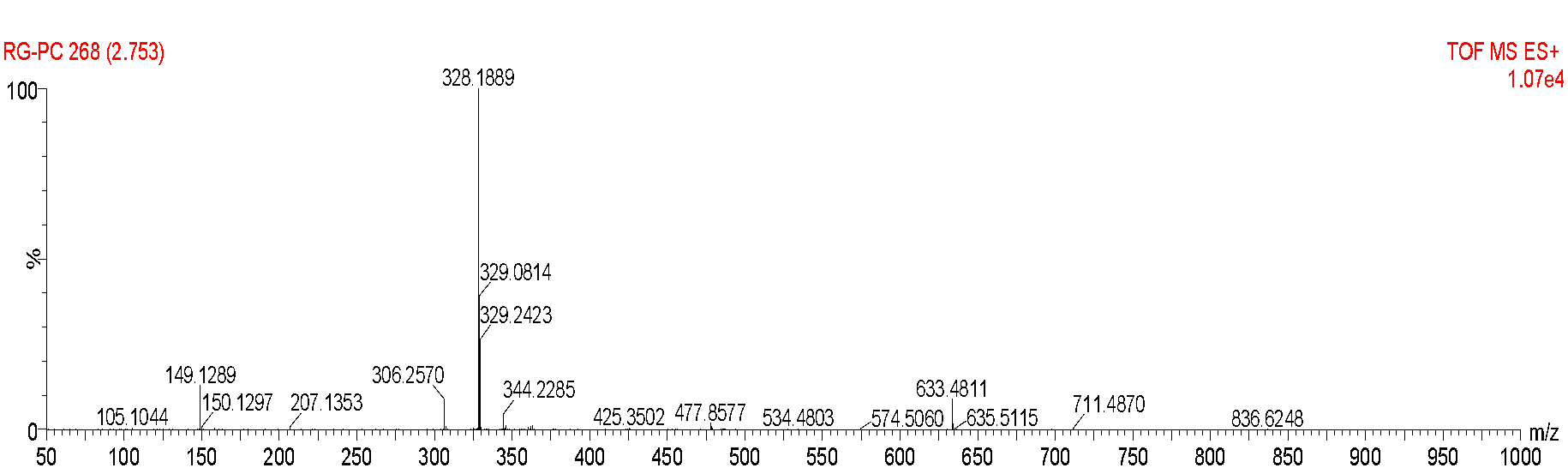


**Figure S2.10.** HRMS spectra of **P3**.


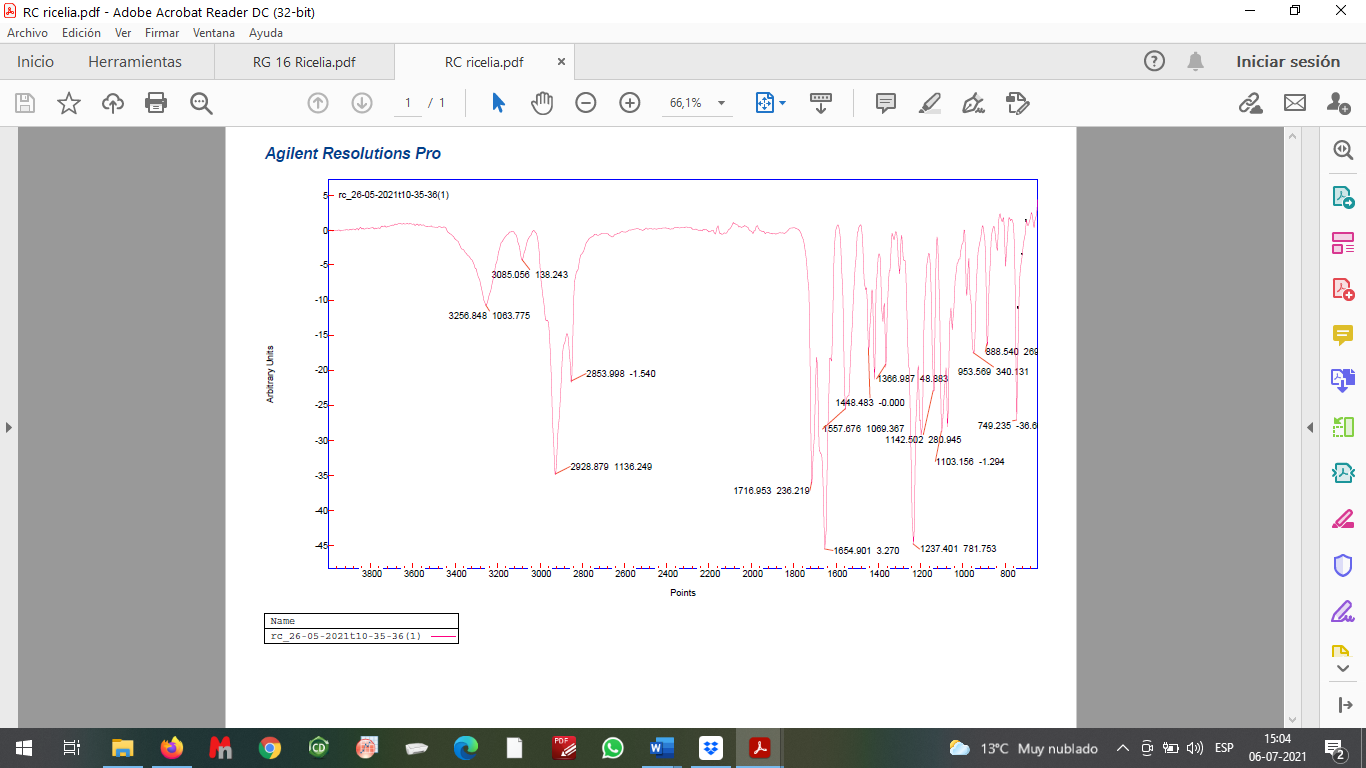


**Figure S2.11.** FT-IR spectra of **P3**.


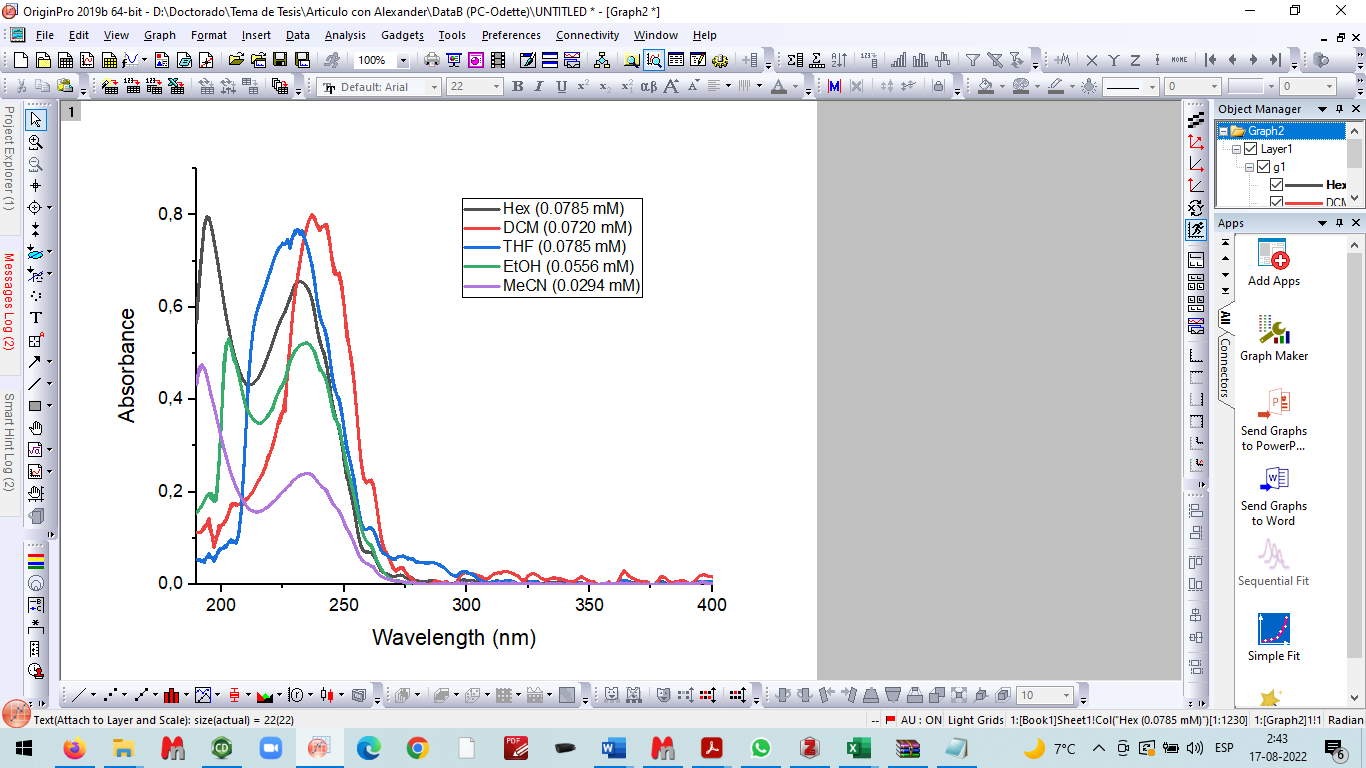


**Figure S2.12.** UV spectra and solvents comparison of **P3**.

**
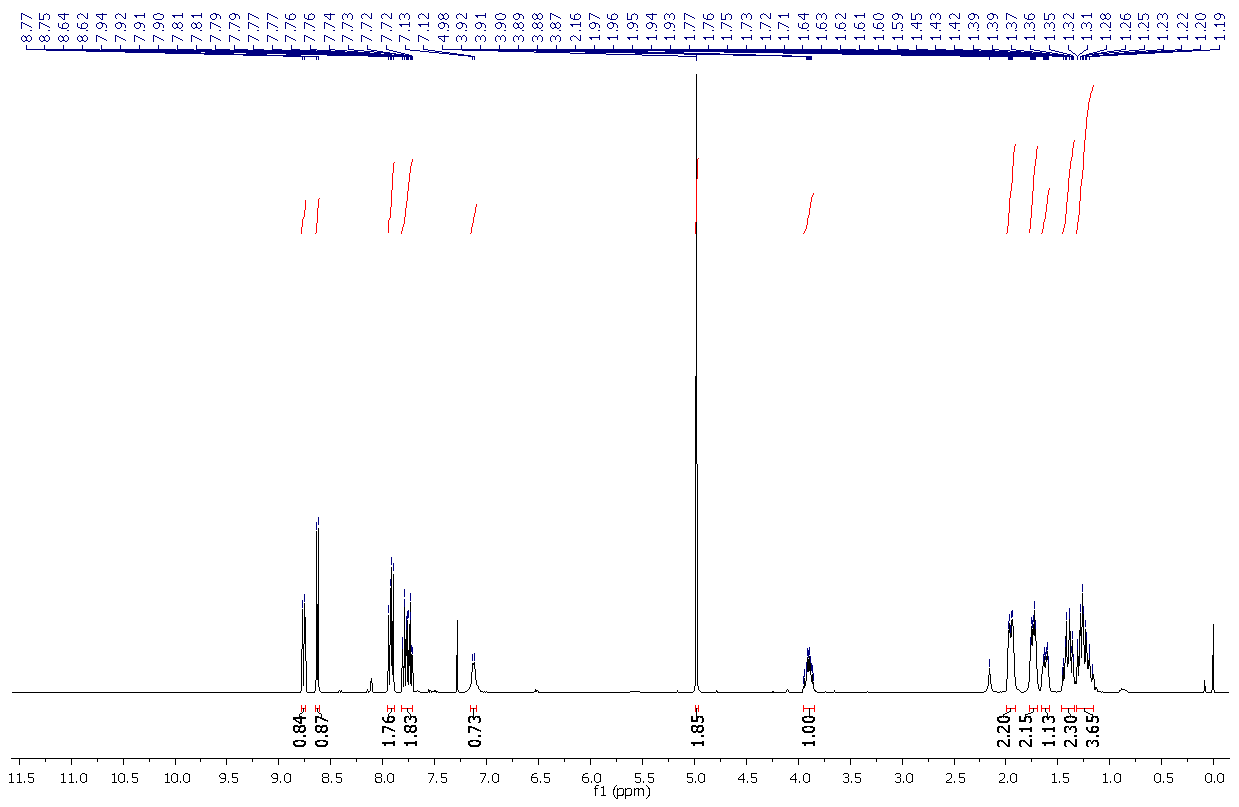
**

**
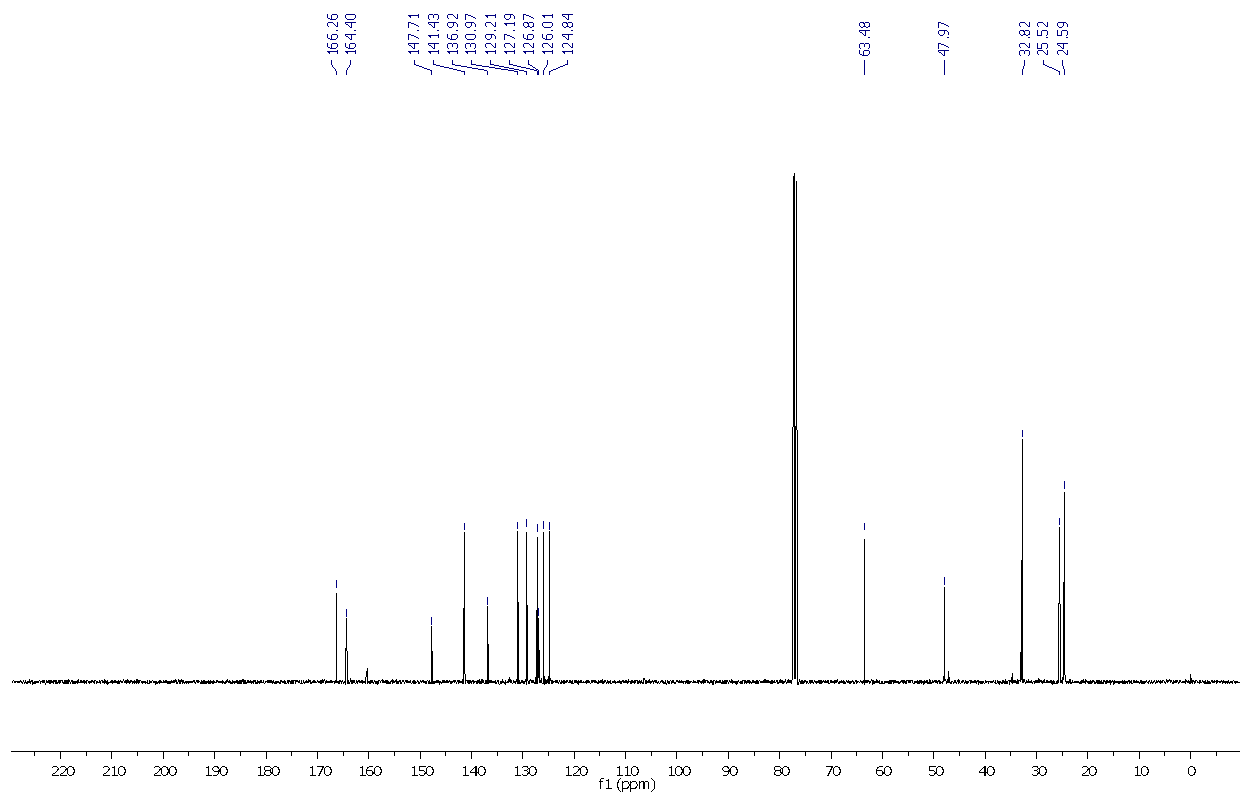
**

**Figure S2.13.** (Top) 400 MHz ^1^H NMR and (bottom) 100 MHz ^13^C NMR spectra in CDCl_3_ of **P4**.


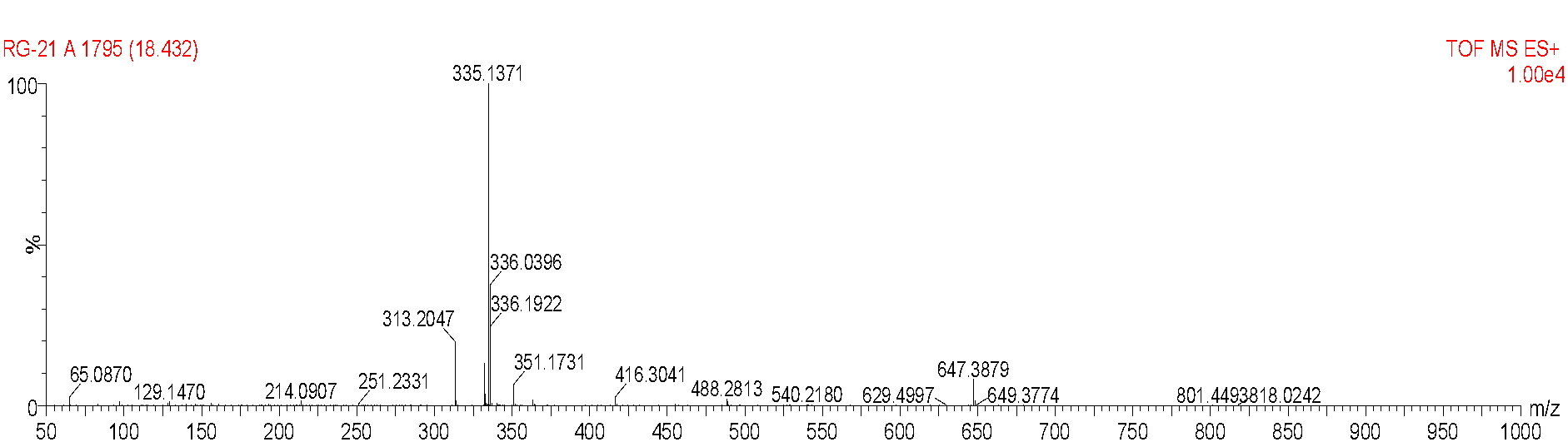


**Figure S2.14.** HRMS spectra of **P4**.


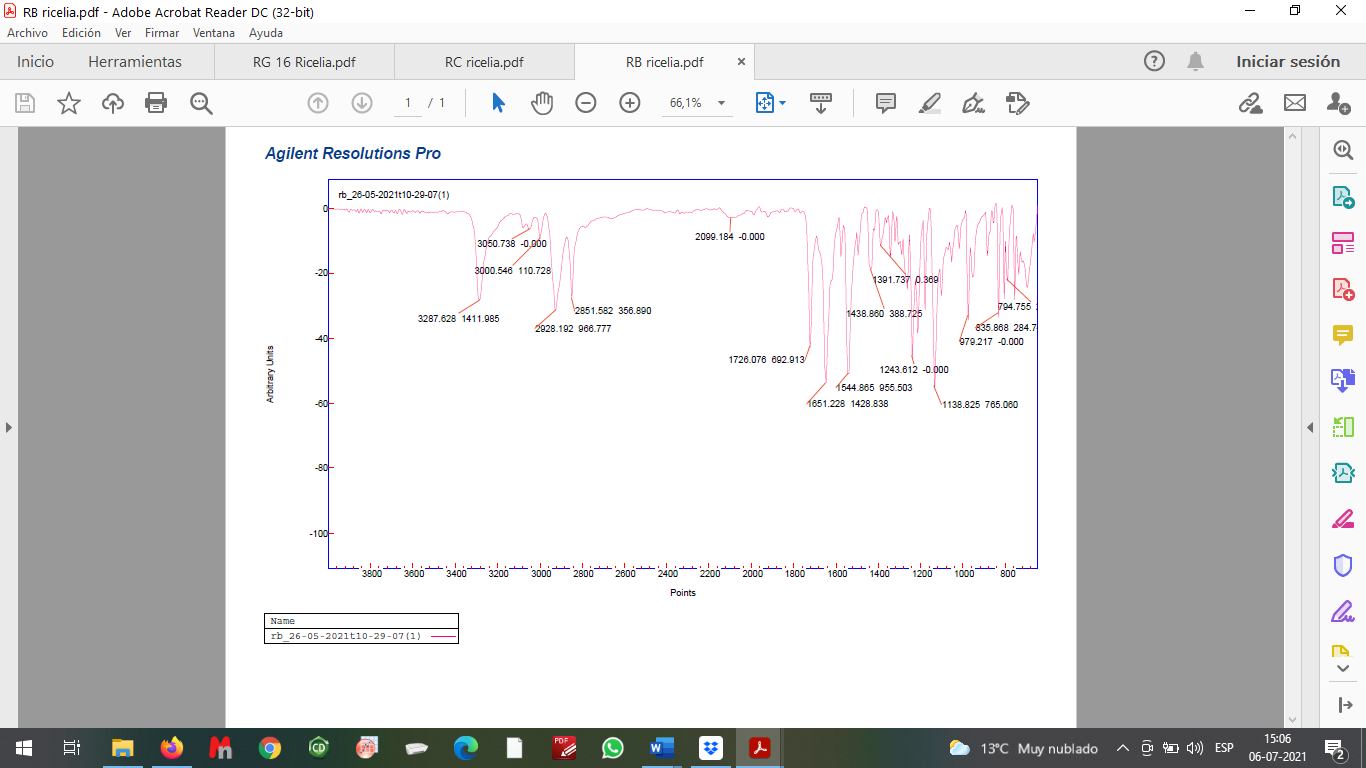


**Figure S2.15.** FT-IR spectra of **P4**.


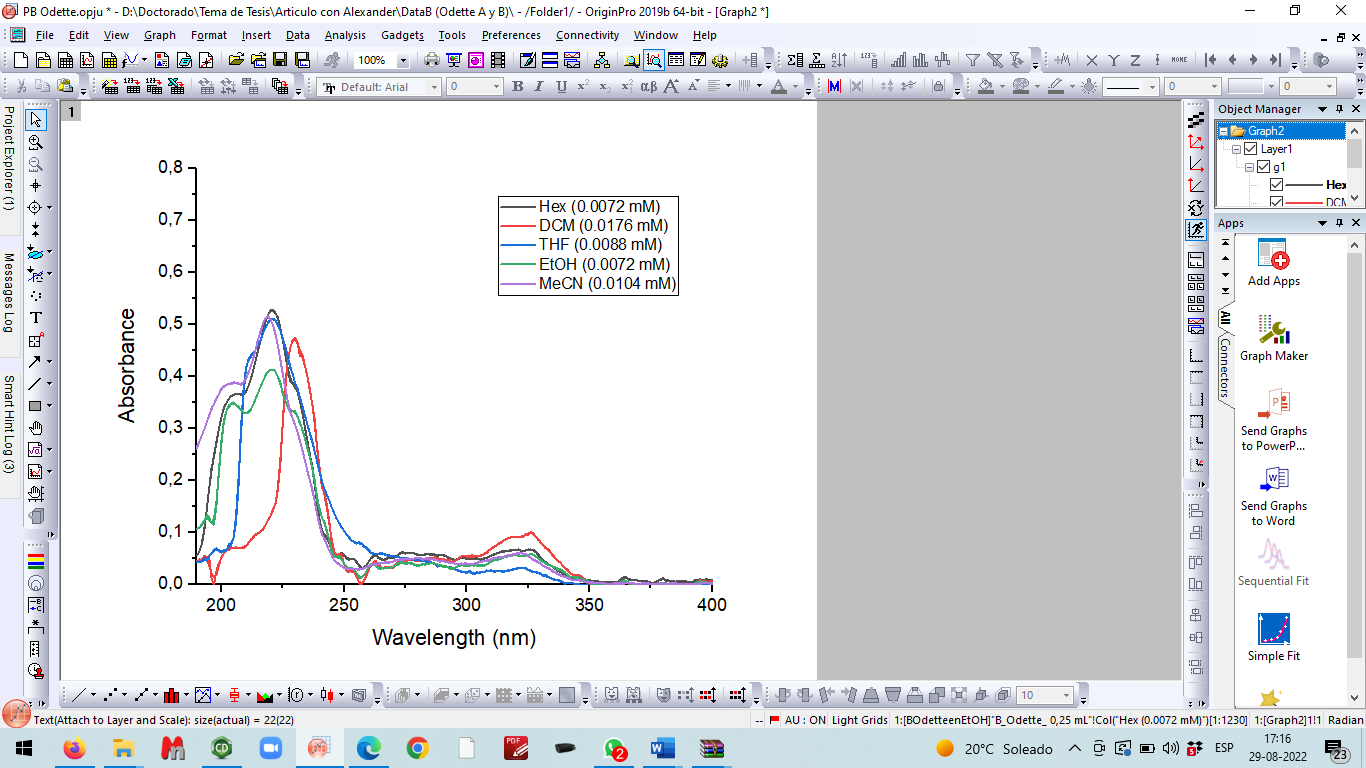


**Figure S2.16.** UV spectra and solvents comparison of **P4**.

*residual signs of the solvent used for the analysis

**Figure S2.17.** (Top) 400 MHz ^1^H NMR and (bottom) 100 MHz ^13^C NMR spectra in CDCl_3_ of **P5**.


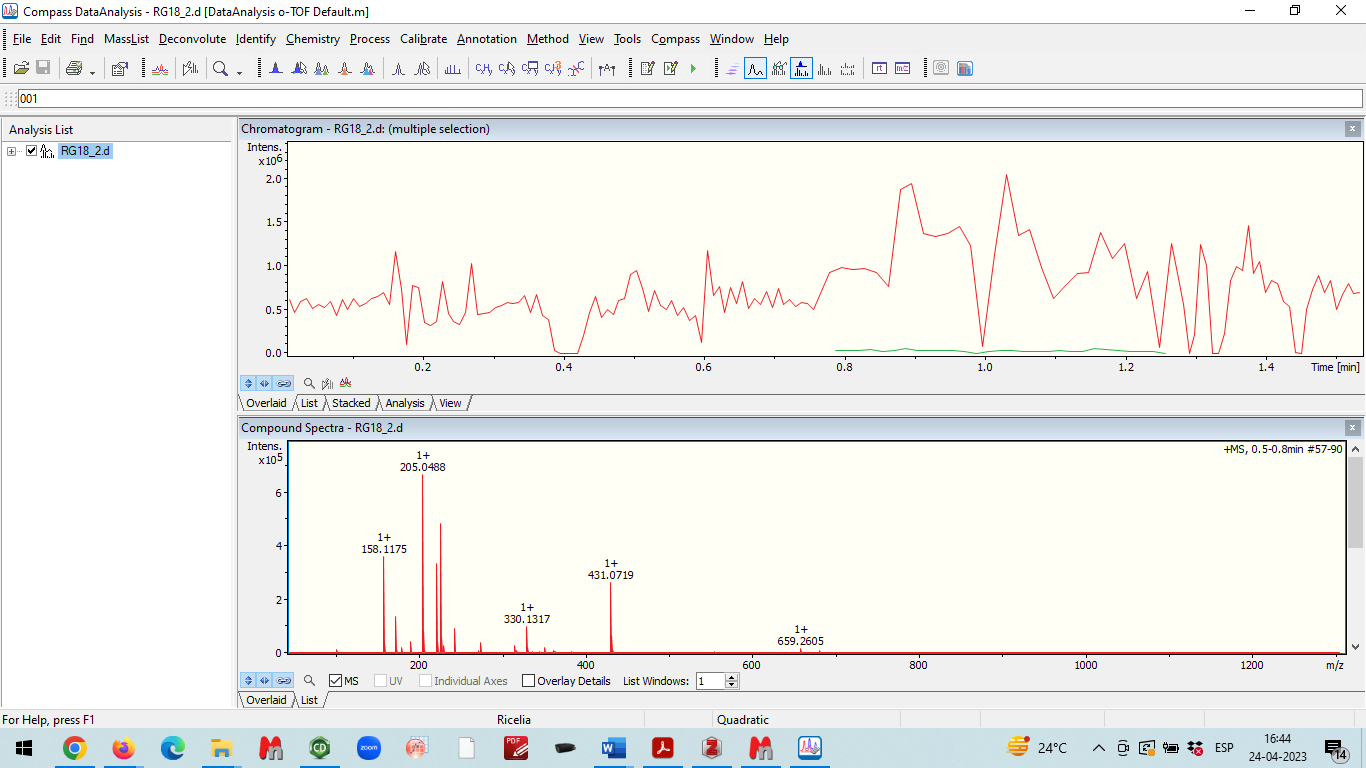


**Figure S2.18.** HRMS spectra of **P5**.


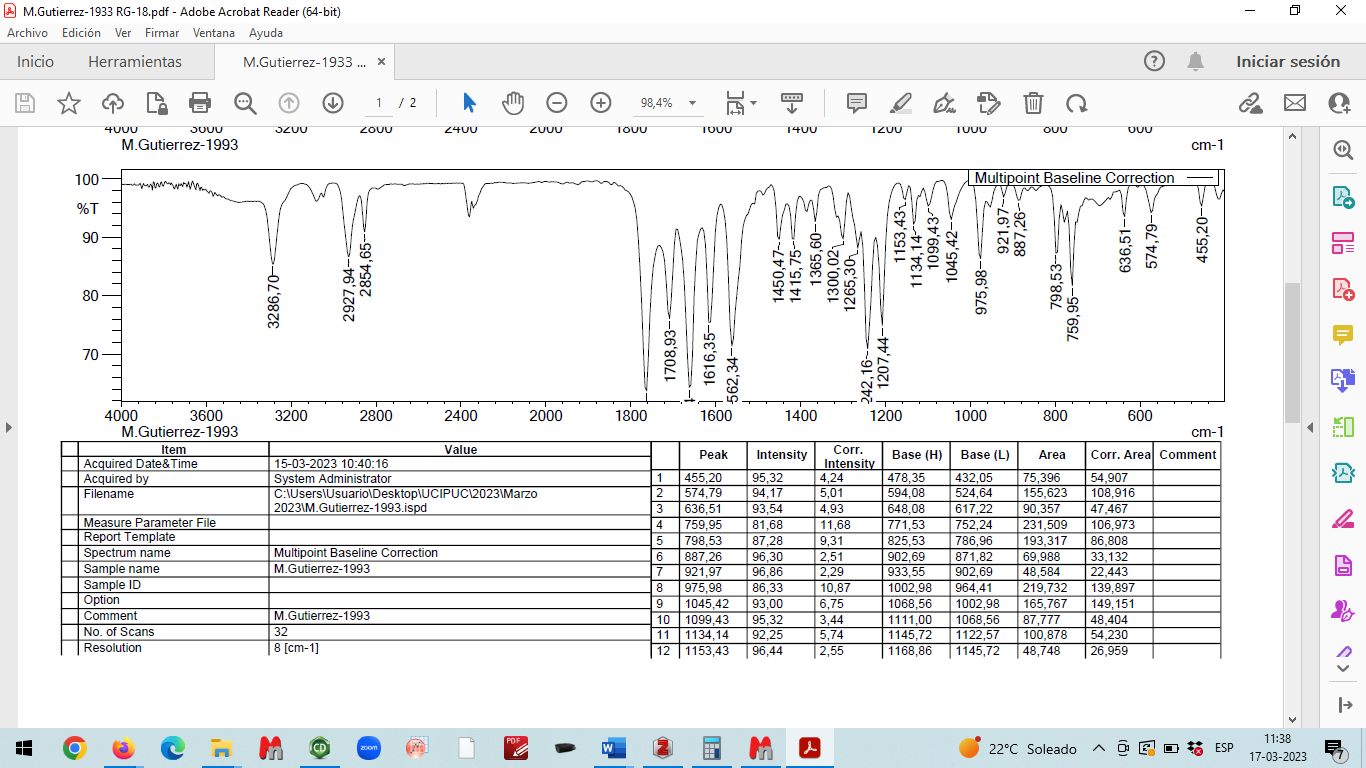


**Figure S2.19.** FT-IR spectra of **P5**.


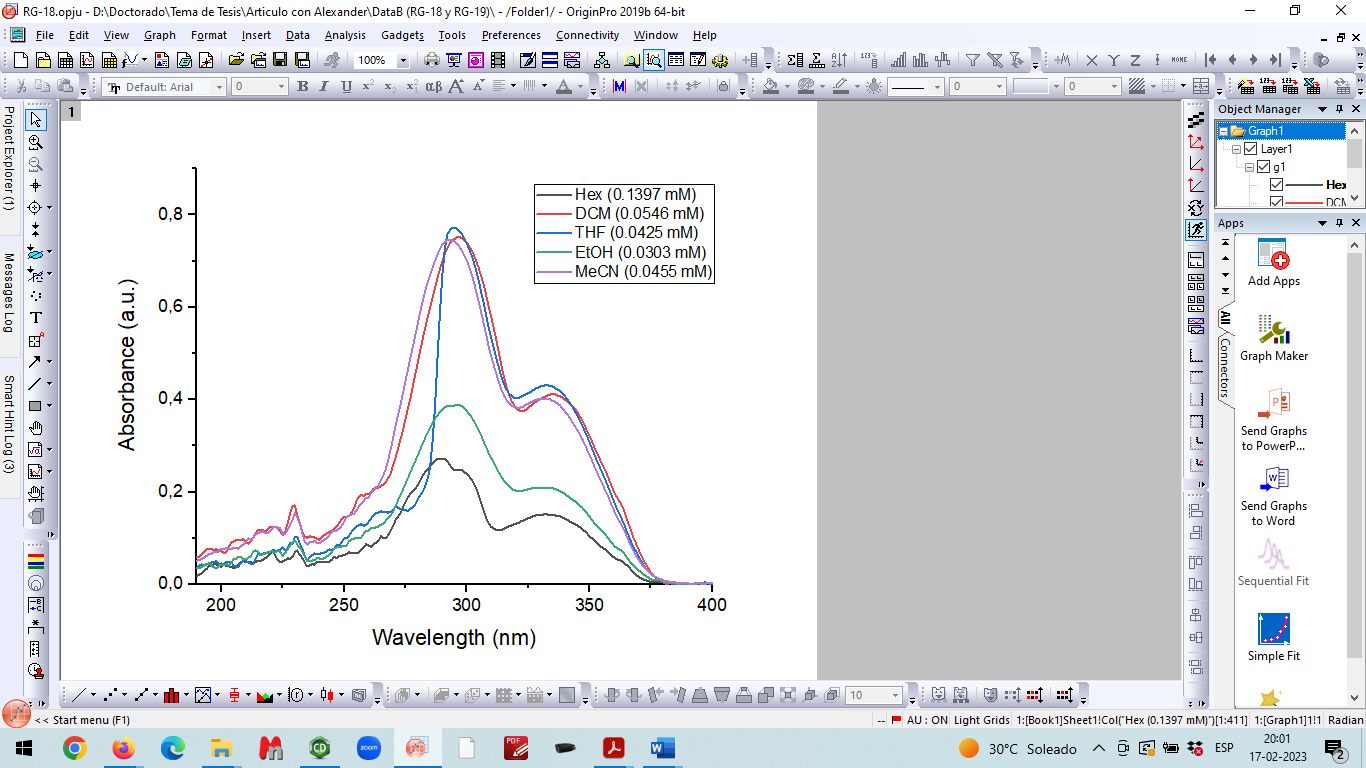


**Figure S2.20.** UV spectra and solvents comparison of **P5**.

*residual signs of the solvent used for the analysis

**Figure S2.21.** (Top) 400 MHz ^1^H NMR and (bottom) 100 MHz ^13^C NMR spectra in CDCl_3_ of **P6**.


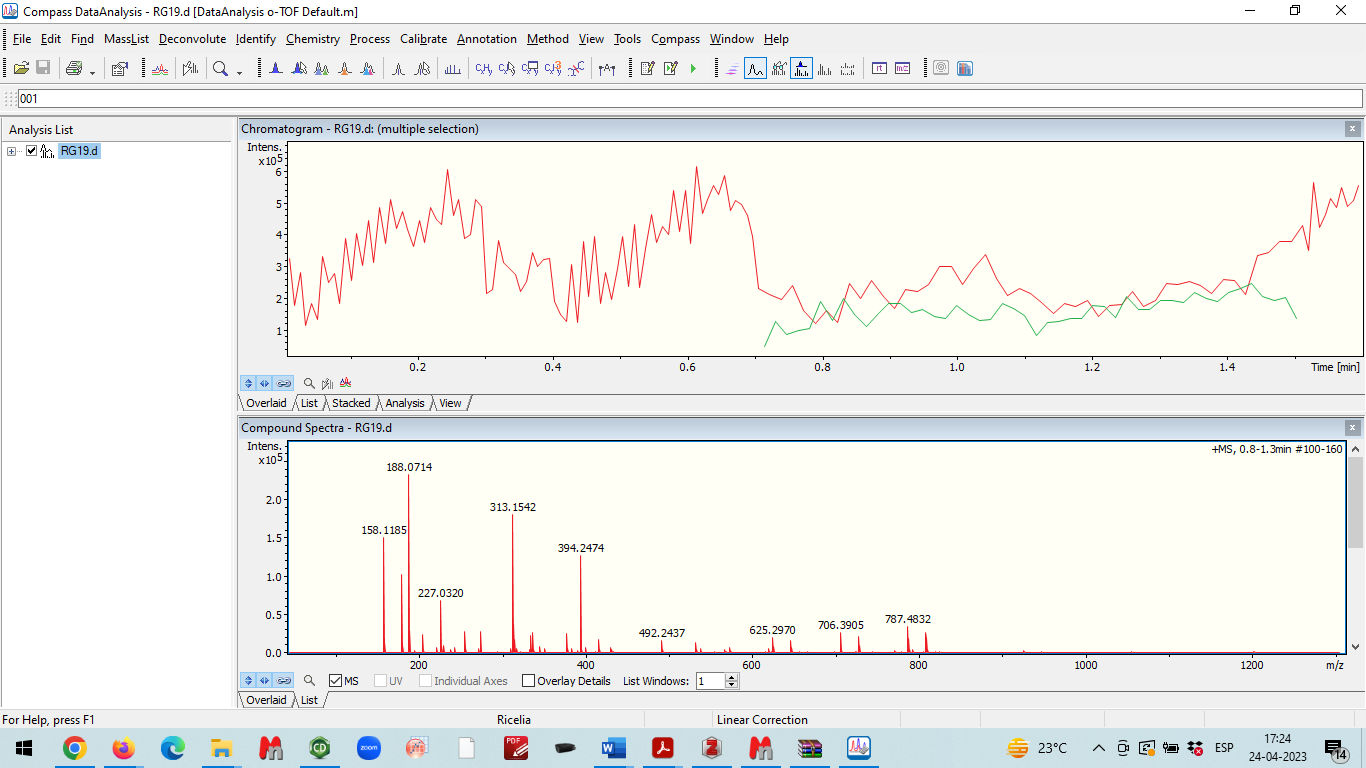


**Figure S2.22.** HRMS spectra of **P6**.


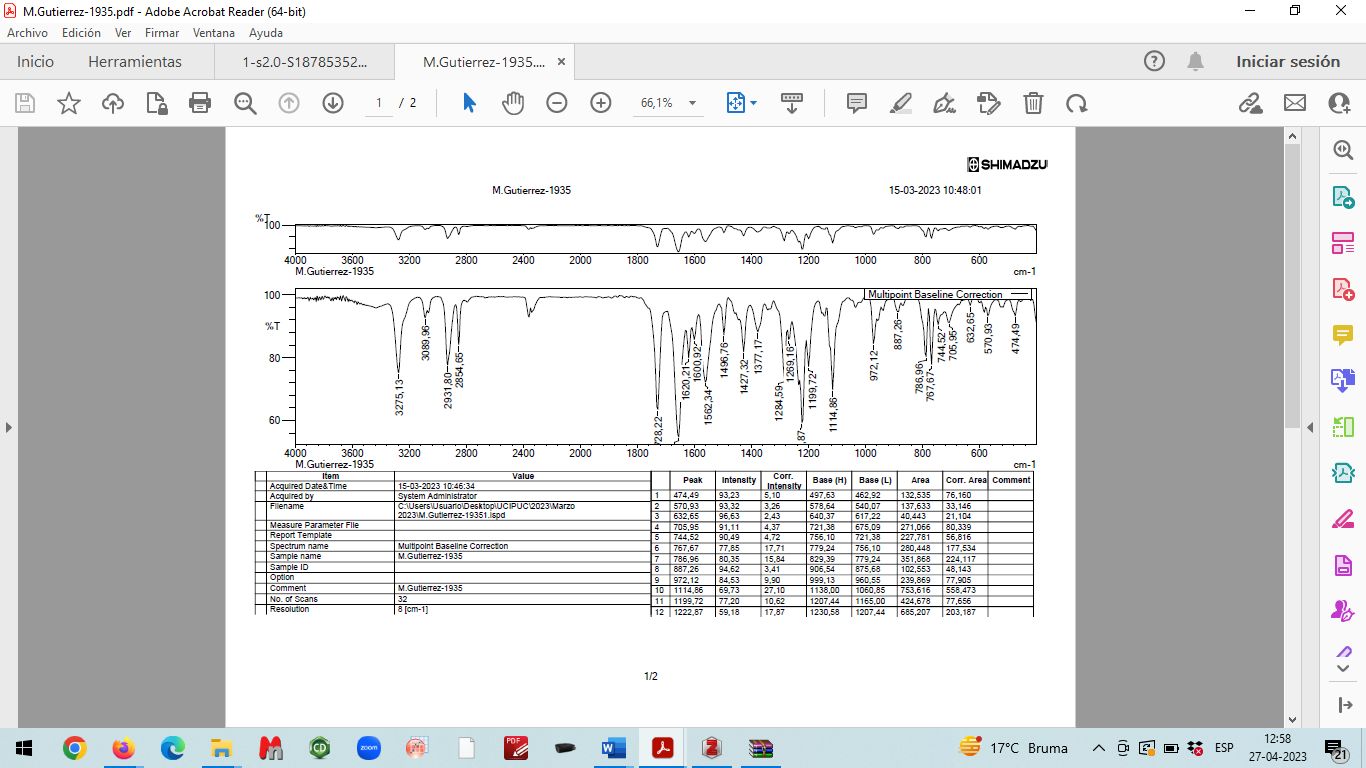


**Figure S2.23.** FT-IR spectra of **P6**.


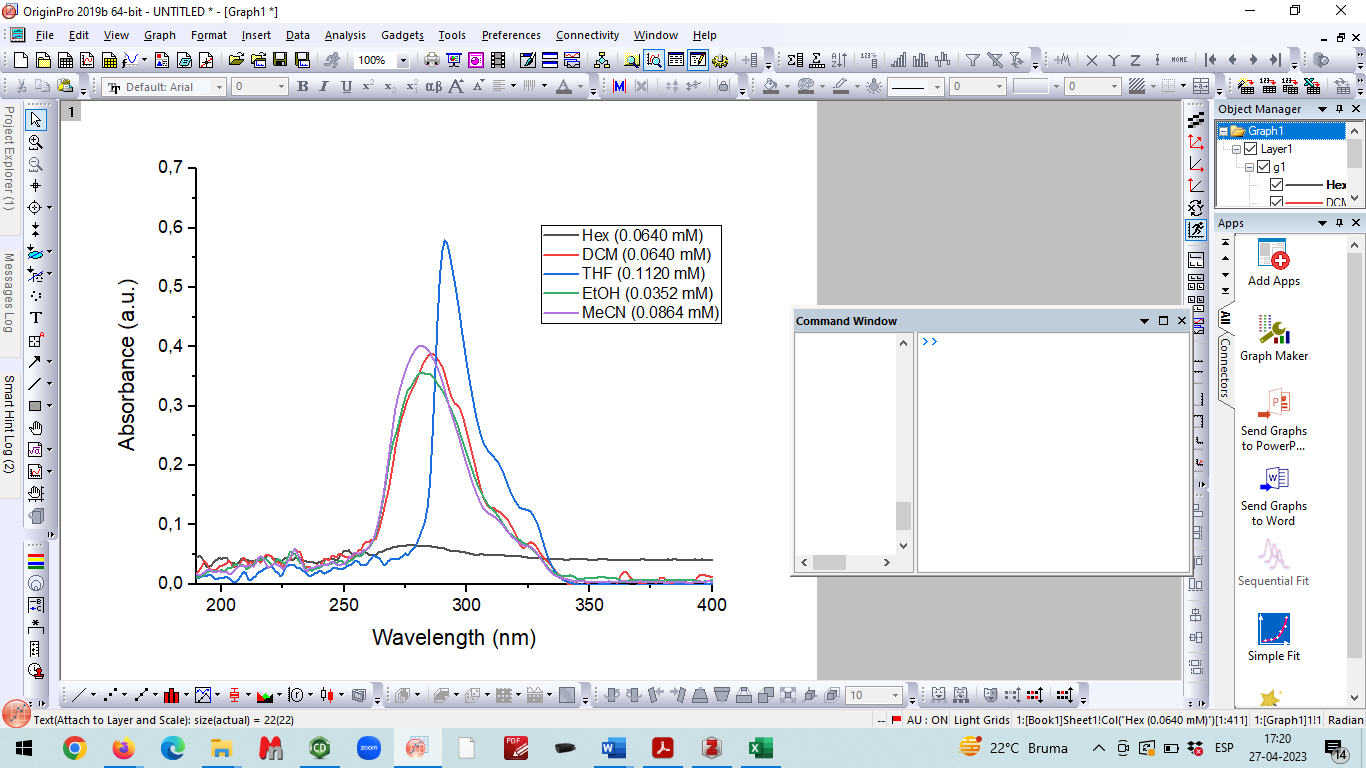


**Figure S2.24.** UV spectra and solvents comparison of **P6**.


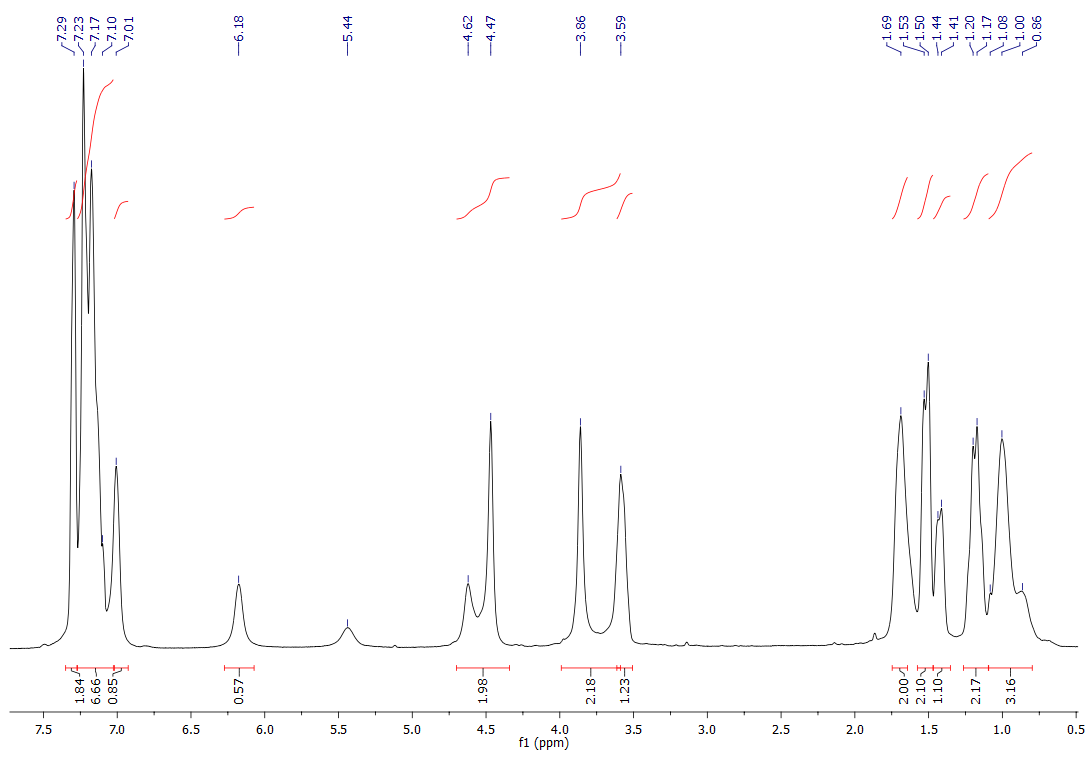


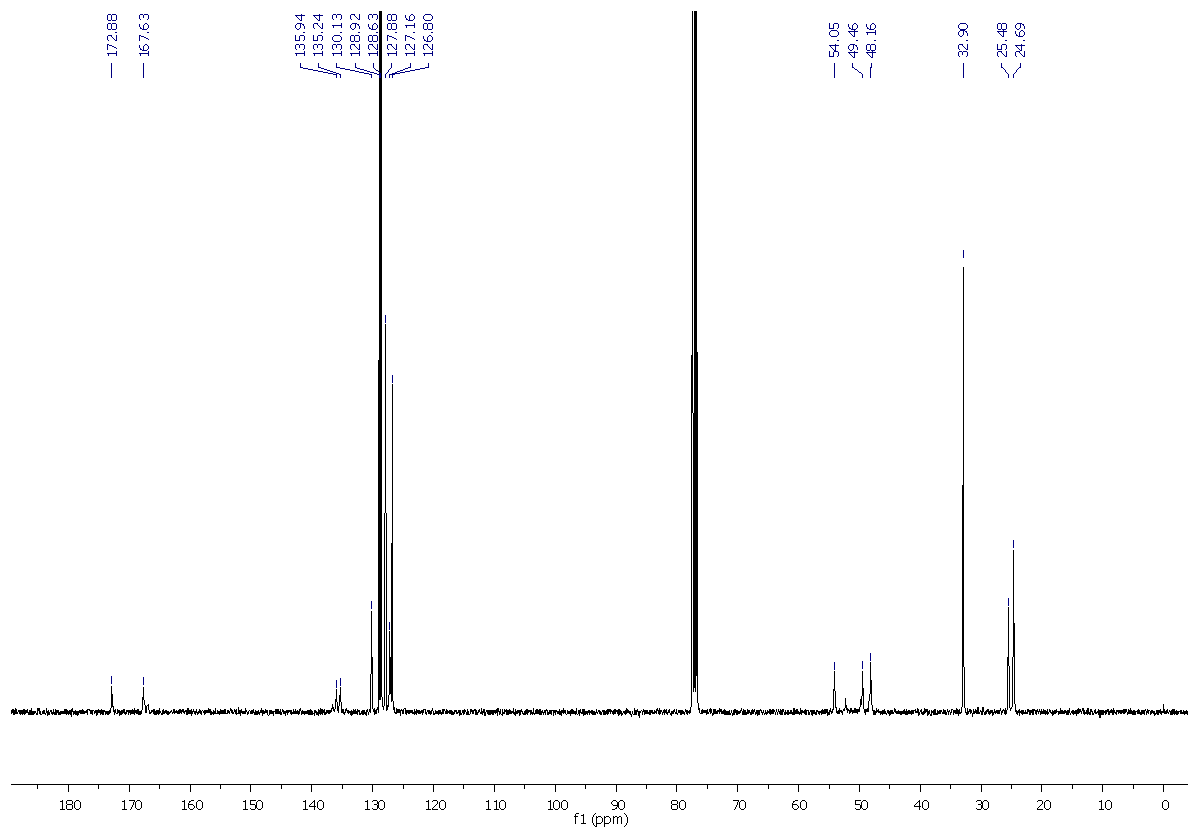


**Figure S2.25.** (Top) 400 MHz ^1^H NMR and (bottom) 100 MHz ^13^C NMR spectra in CDCl_3_ of **U1**.


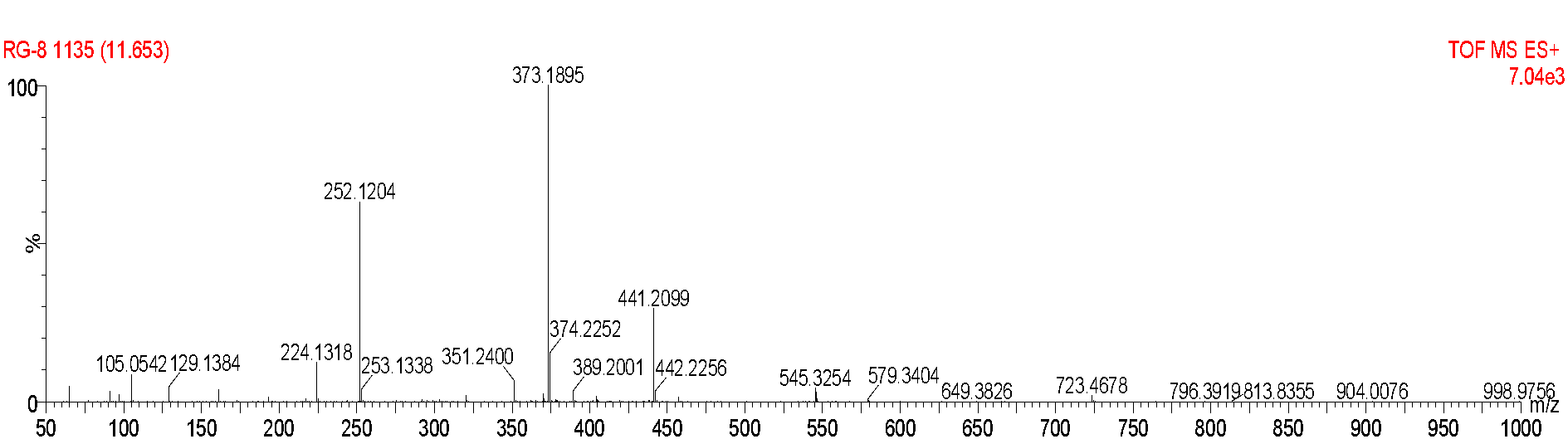


**Figure S2.26.** HRMS spectra of **U1**.


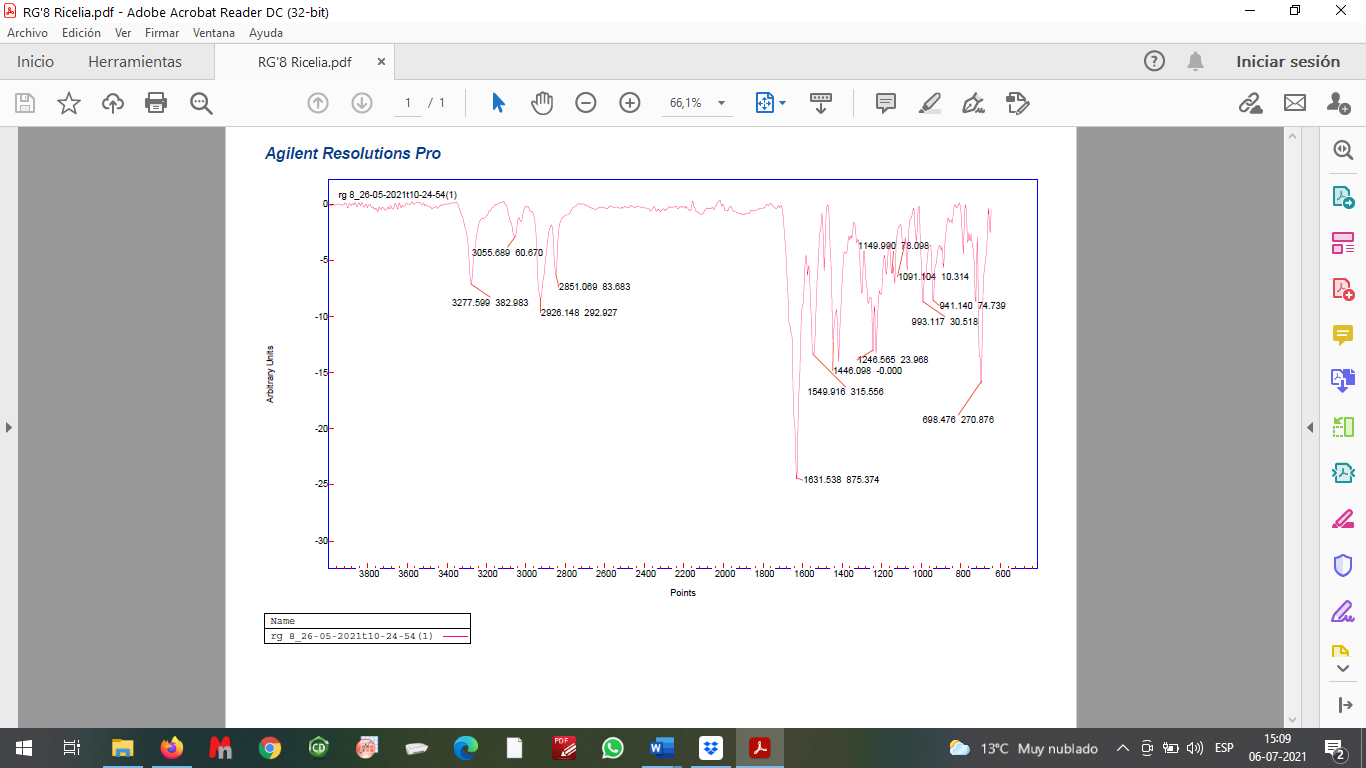


**Figure S2.27.** FT-IR spectra of **U1**.


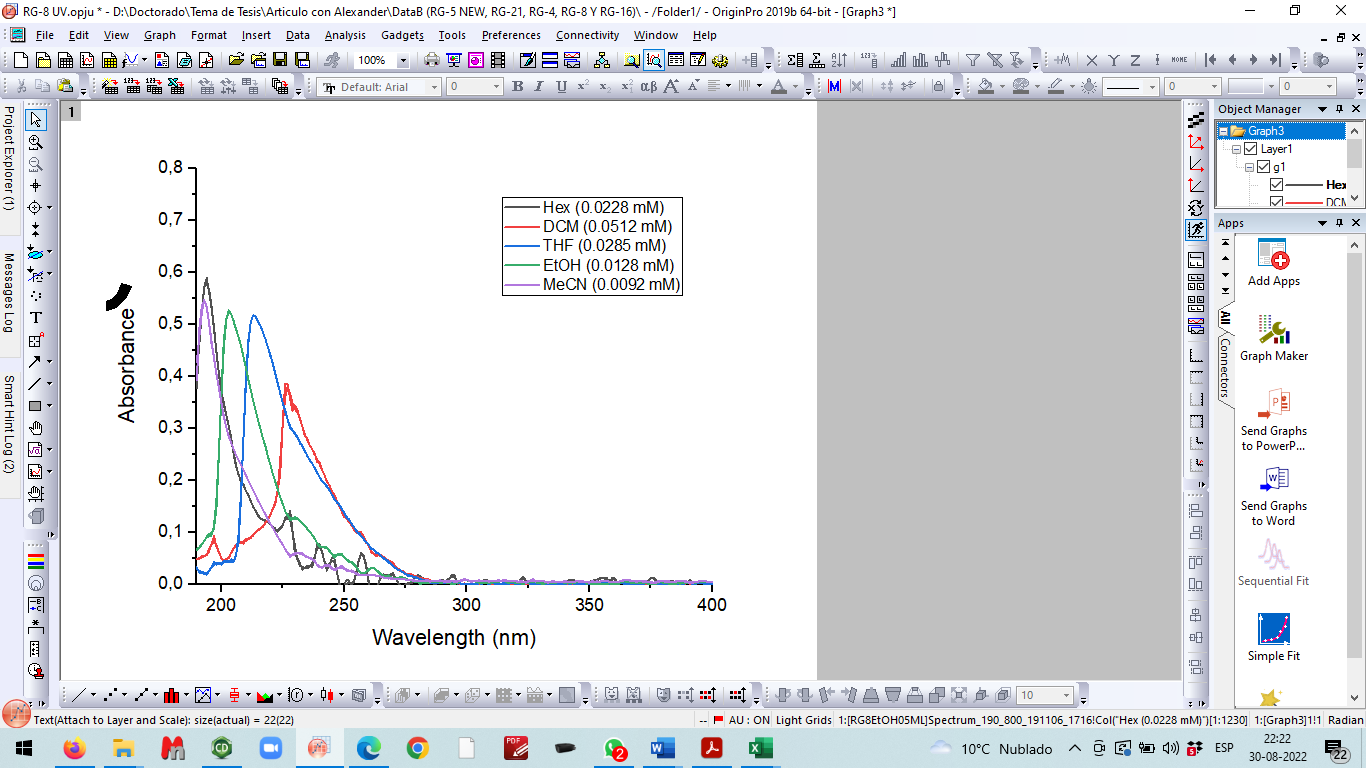


**Figure S2.28.** UV spectra and solvents comparison of **U1**.

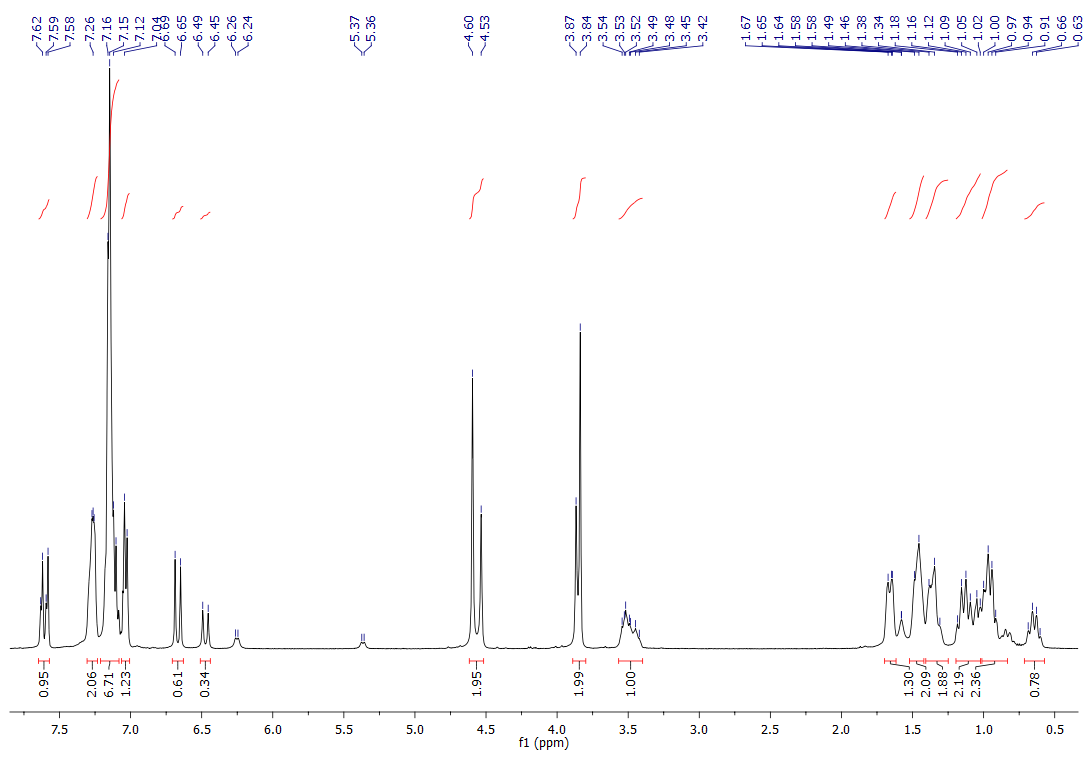


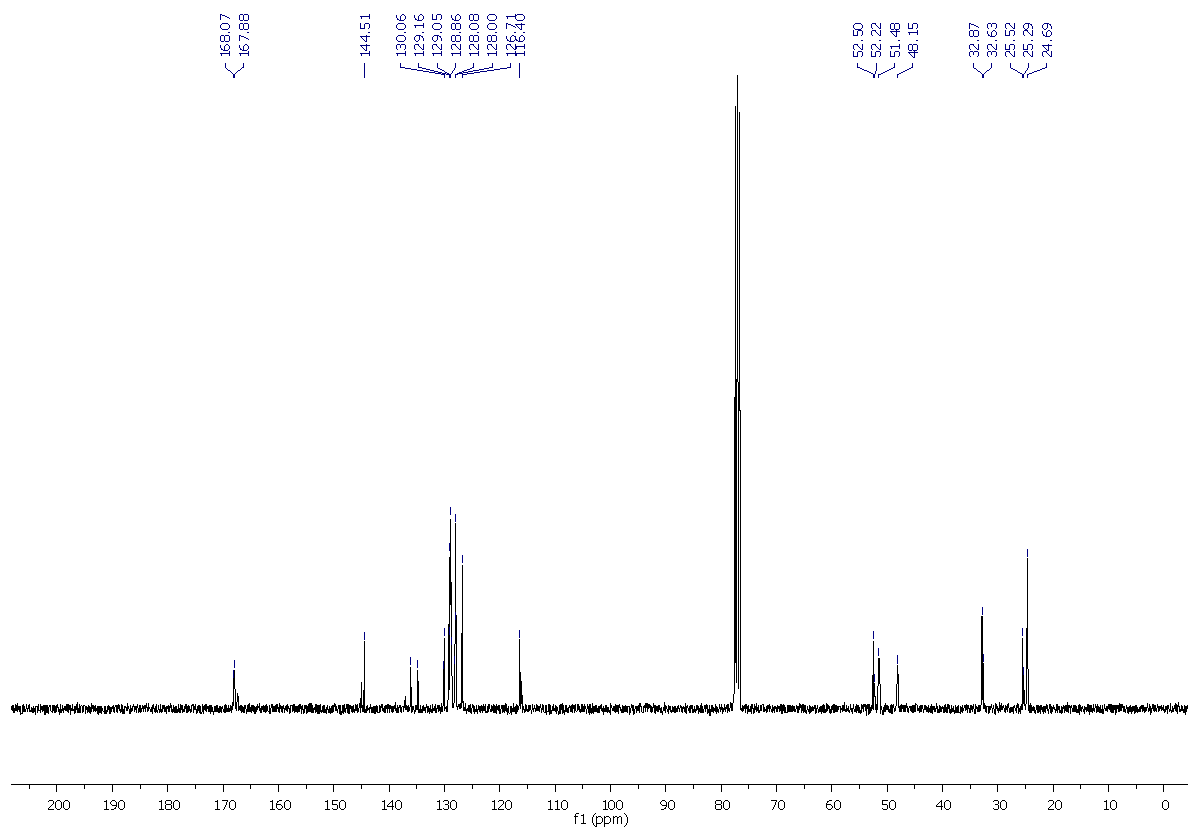


**Figure S2.29.** (Top) 400 MHz ^1^H NMR and (bottom) 100 MHz ^13^C NMR spectra in CDCl_3_ of **U2**.

**
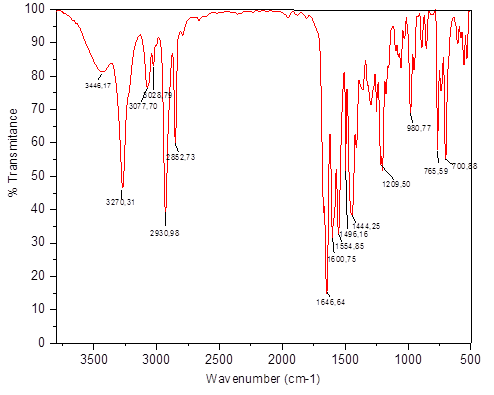
**

**Figure S2.30.** FT-IR spectra of **U2**.


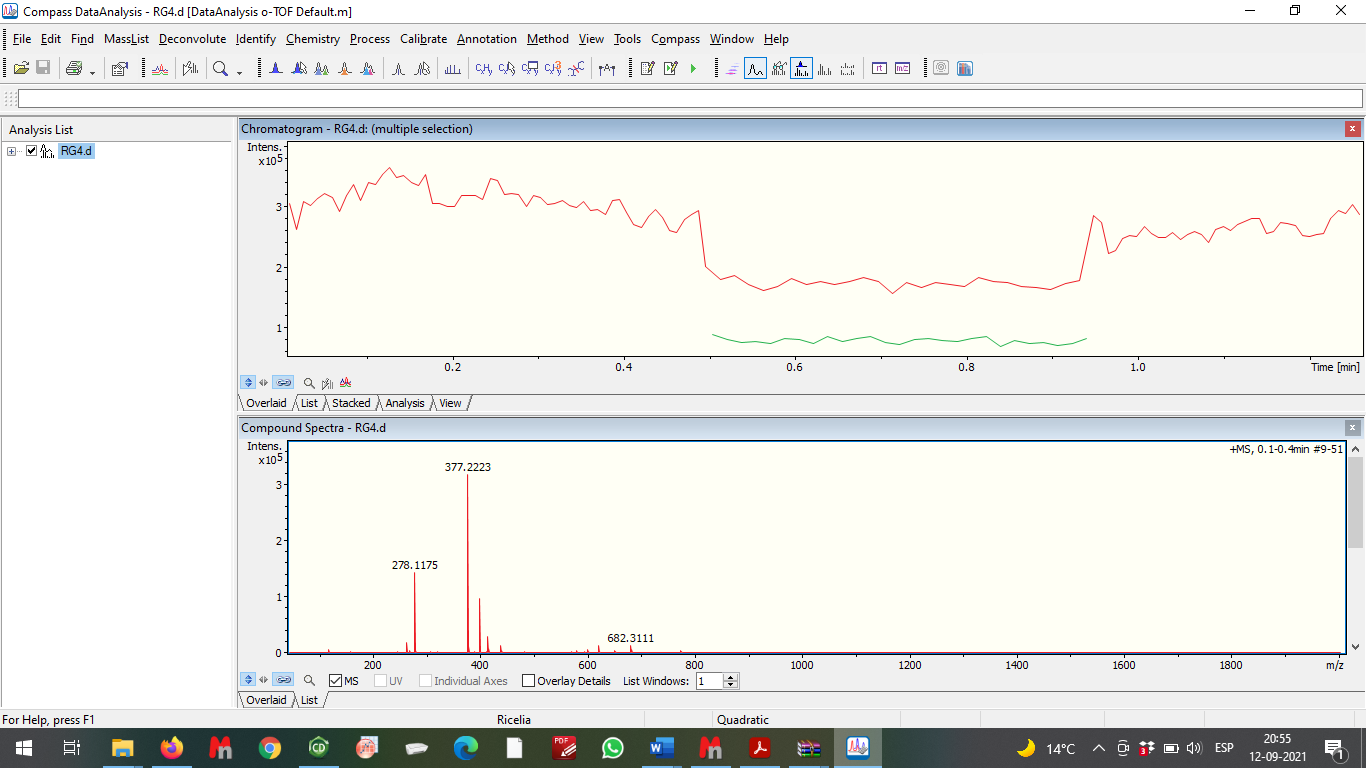


**Figure S2.31.** HRMS spectra of **U2**.


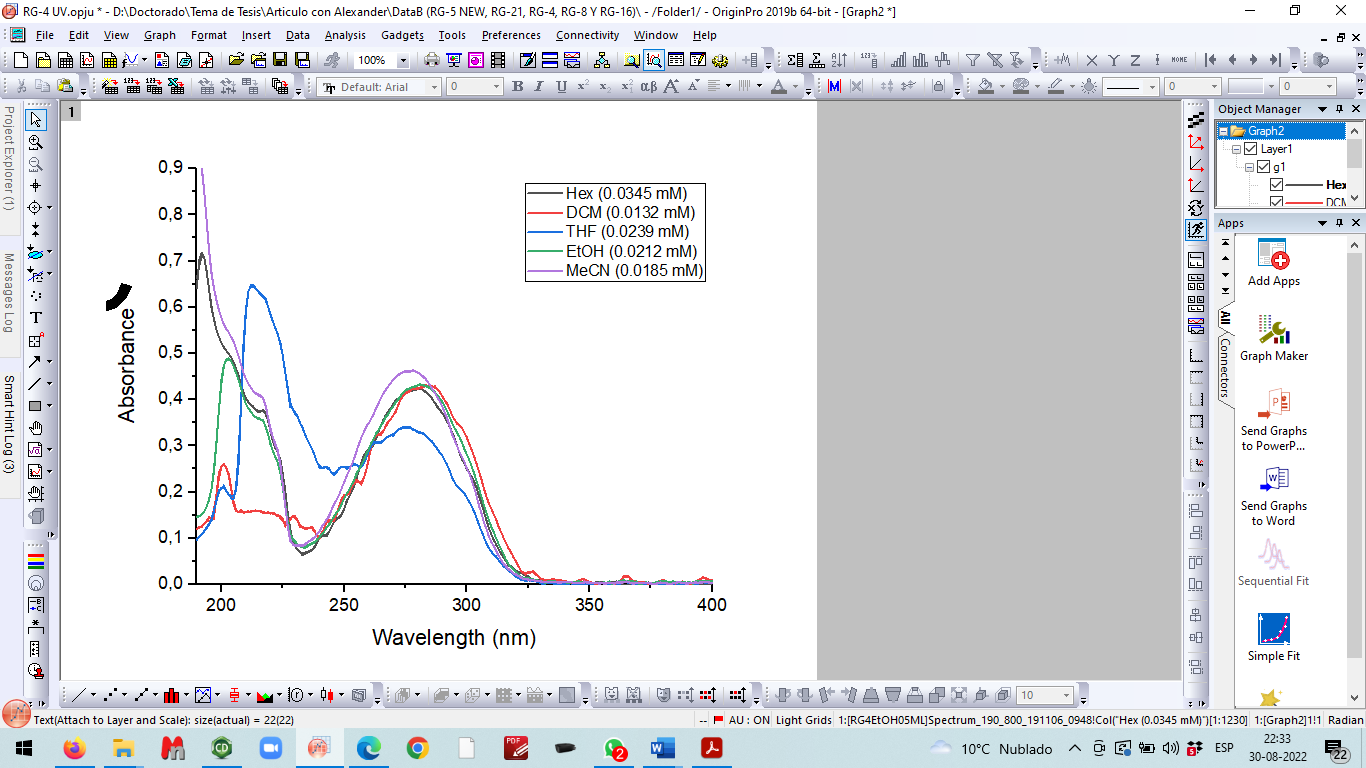


**Figure S2.32.** UV spectra and solvents comparison of **U2**.

**U3** spectroscopic data available at <https://doi.org/10.3390/molecules25081911>


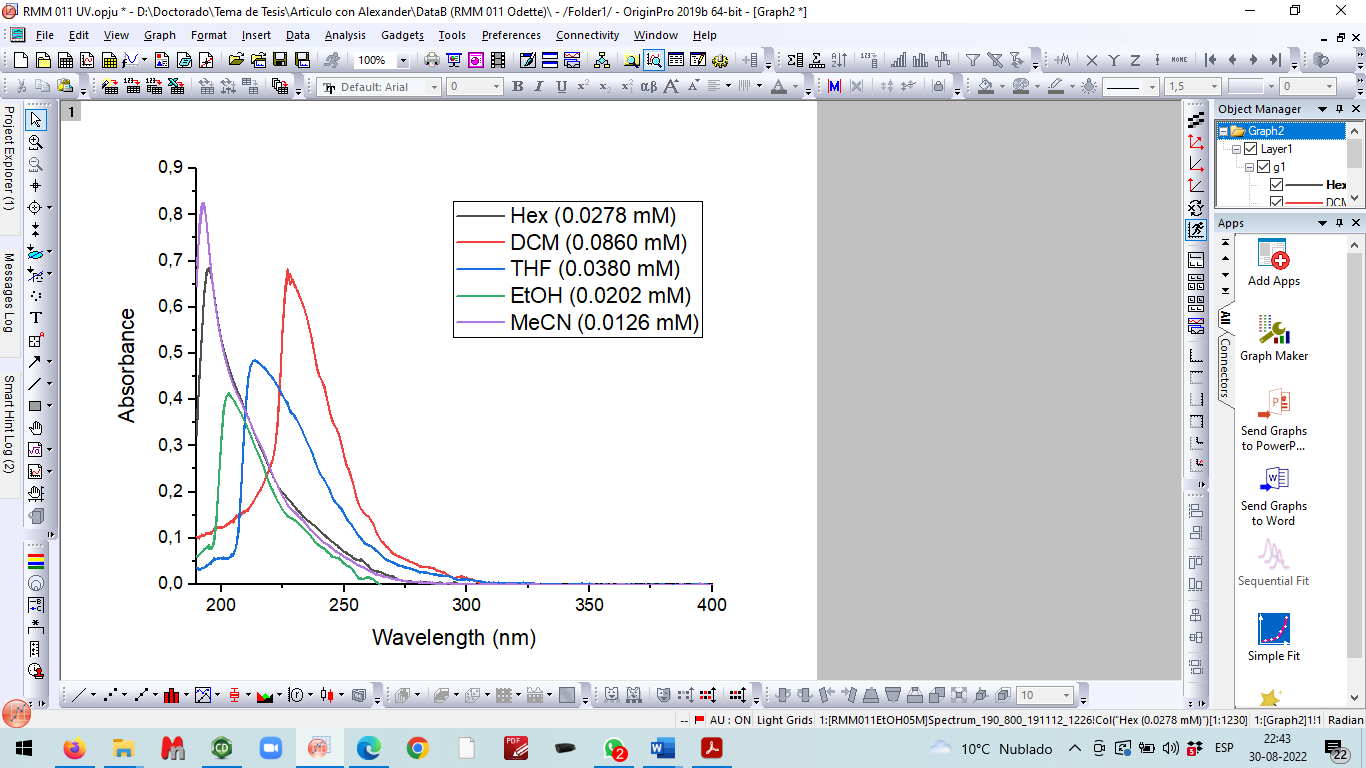


**Figure S2.33.** UV spectra and solvents comparison of **U3**.


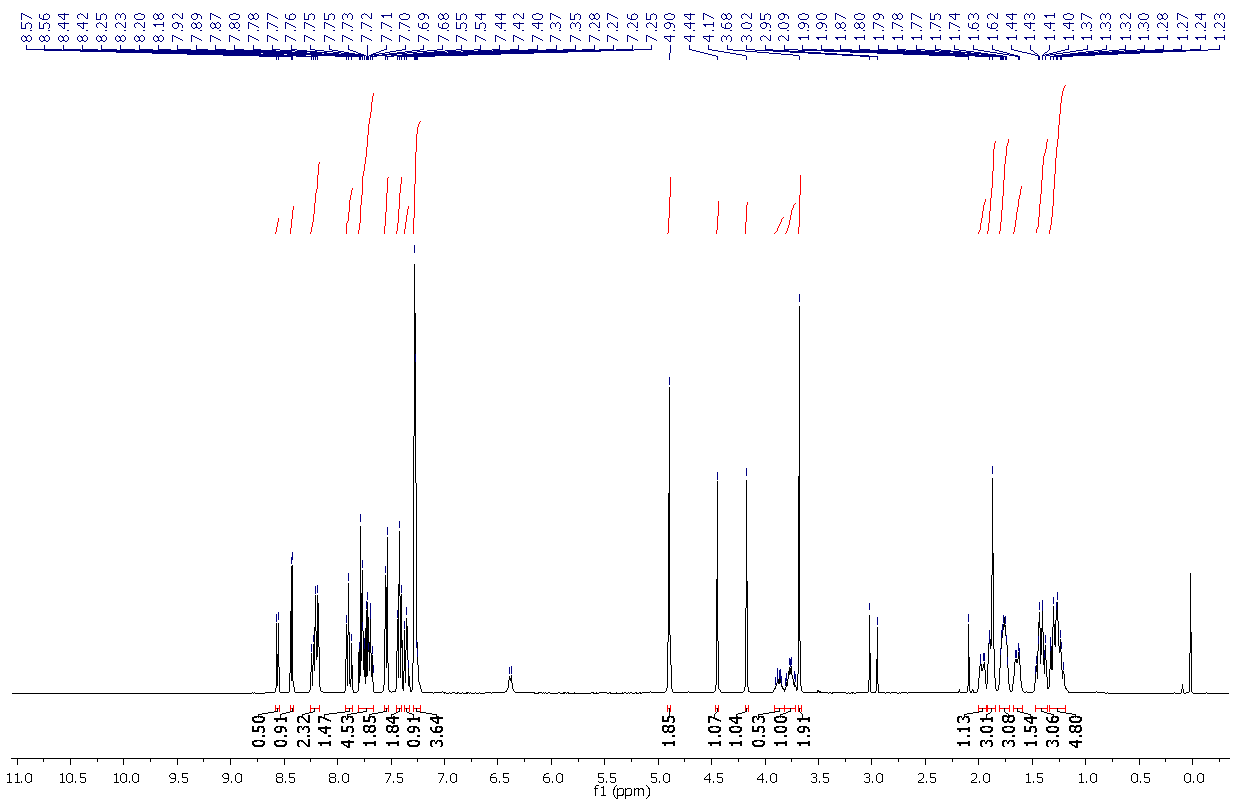


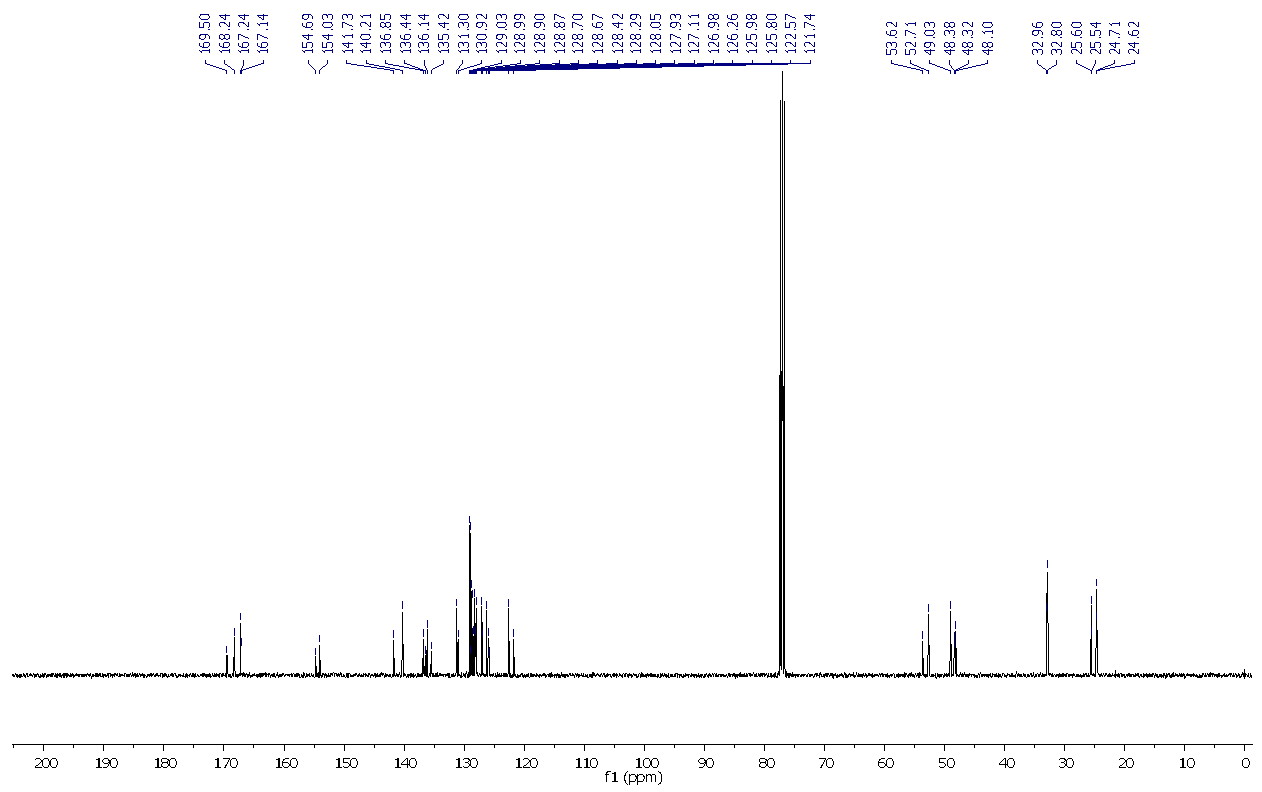


**Figure S2.34.** (Top) 400 MHz ^1^H NMR and (bottom) 100 MHz ^13^C NMR spectra in CDCl_3_ of **U4**.


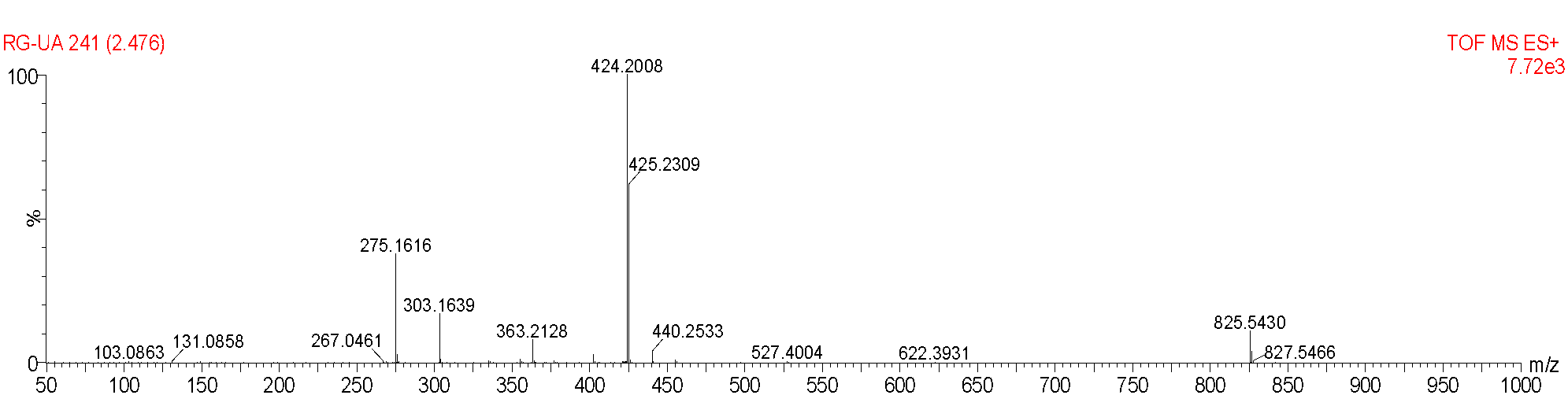


**Figure S2.35.** HRMS spectra of **U4**.


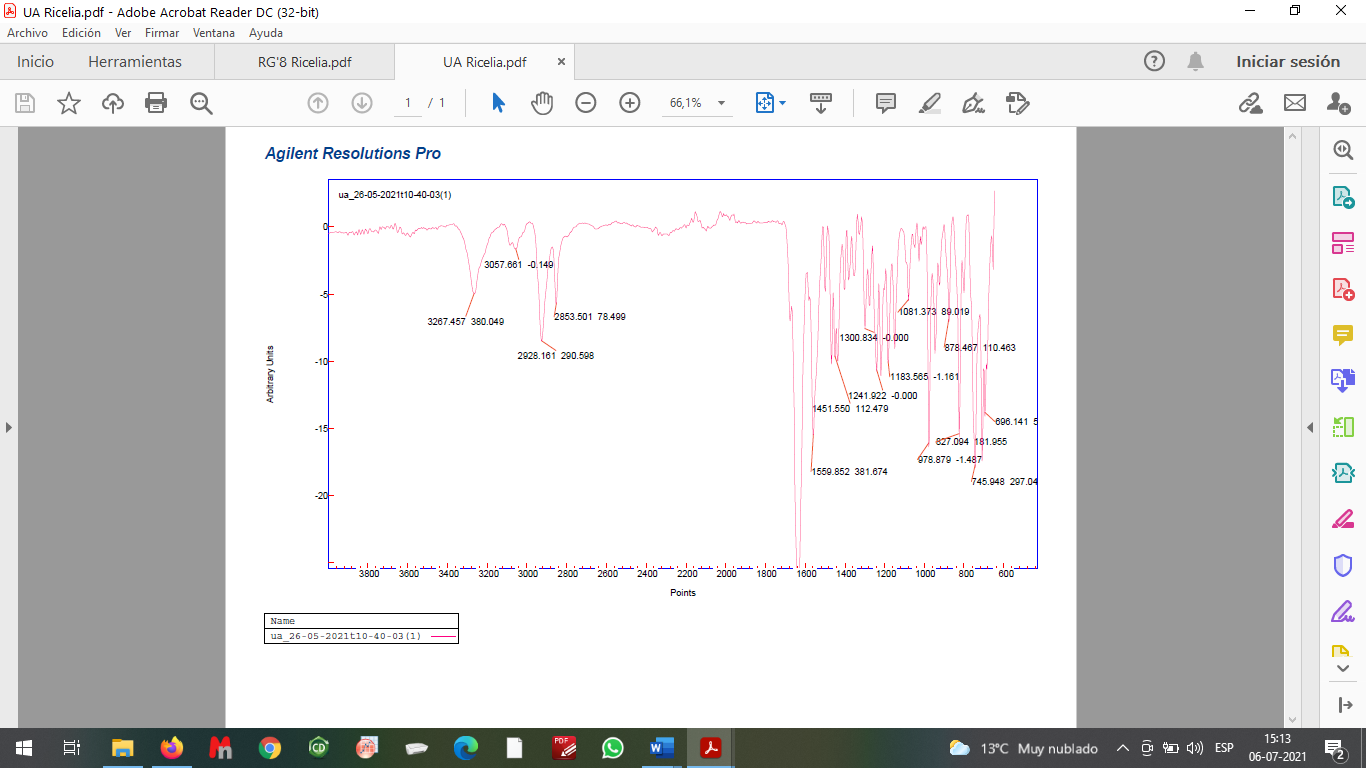


**Figure S2.36.** FT-IR spectra of **U4**.


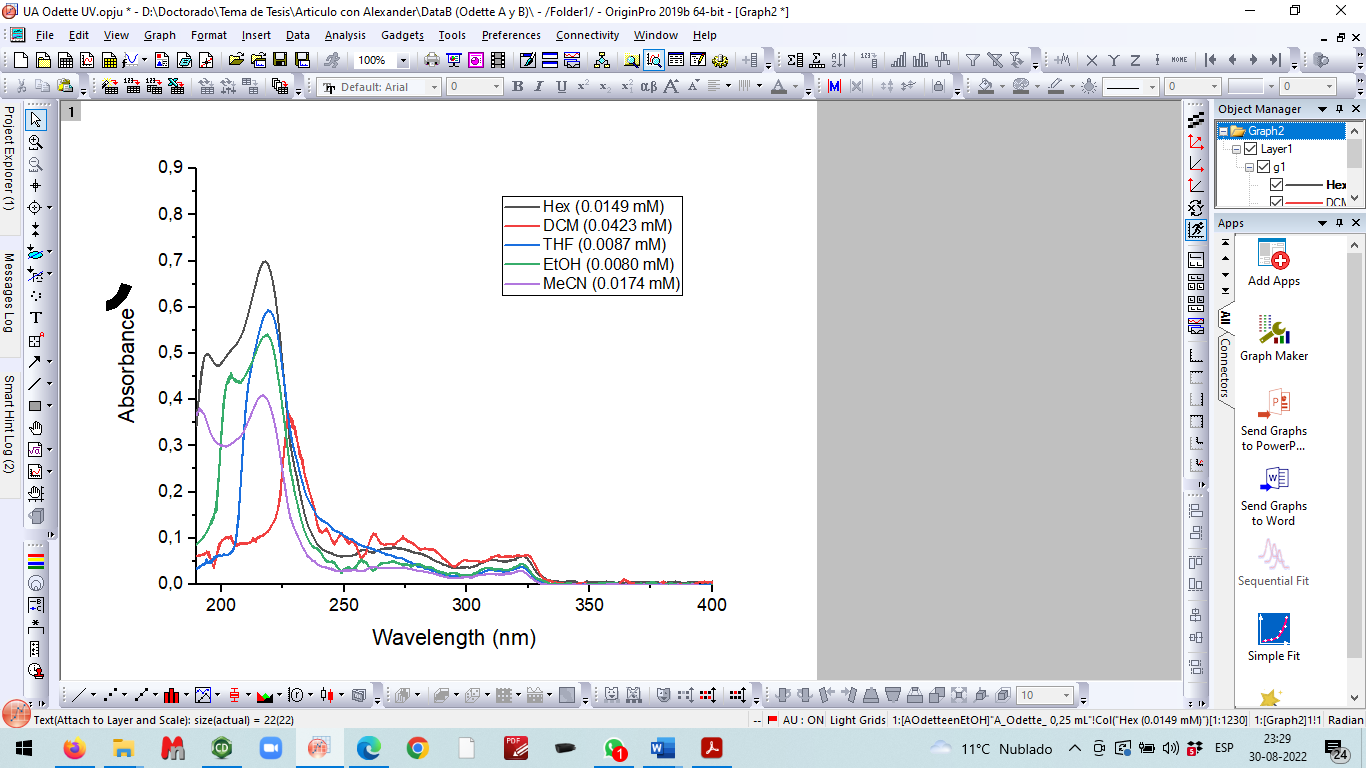


**Figure S2.37.** UV spectra and solvents comparison of **U4**.

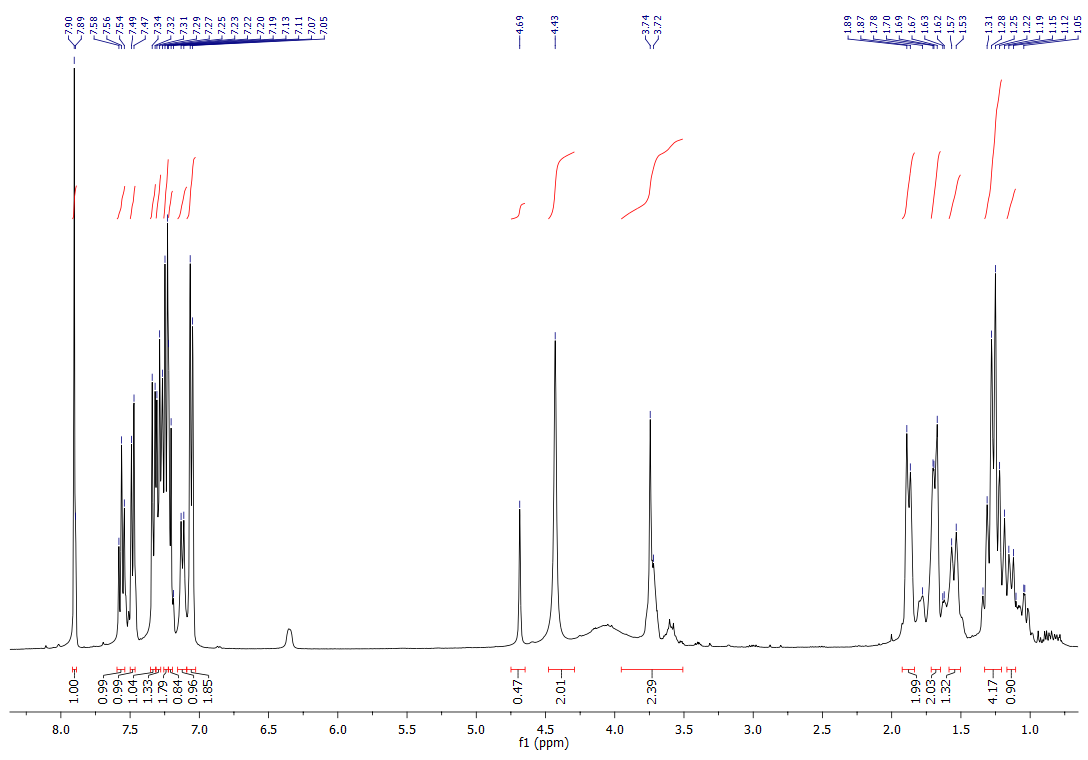


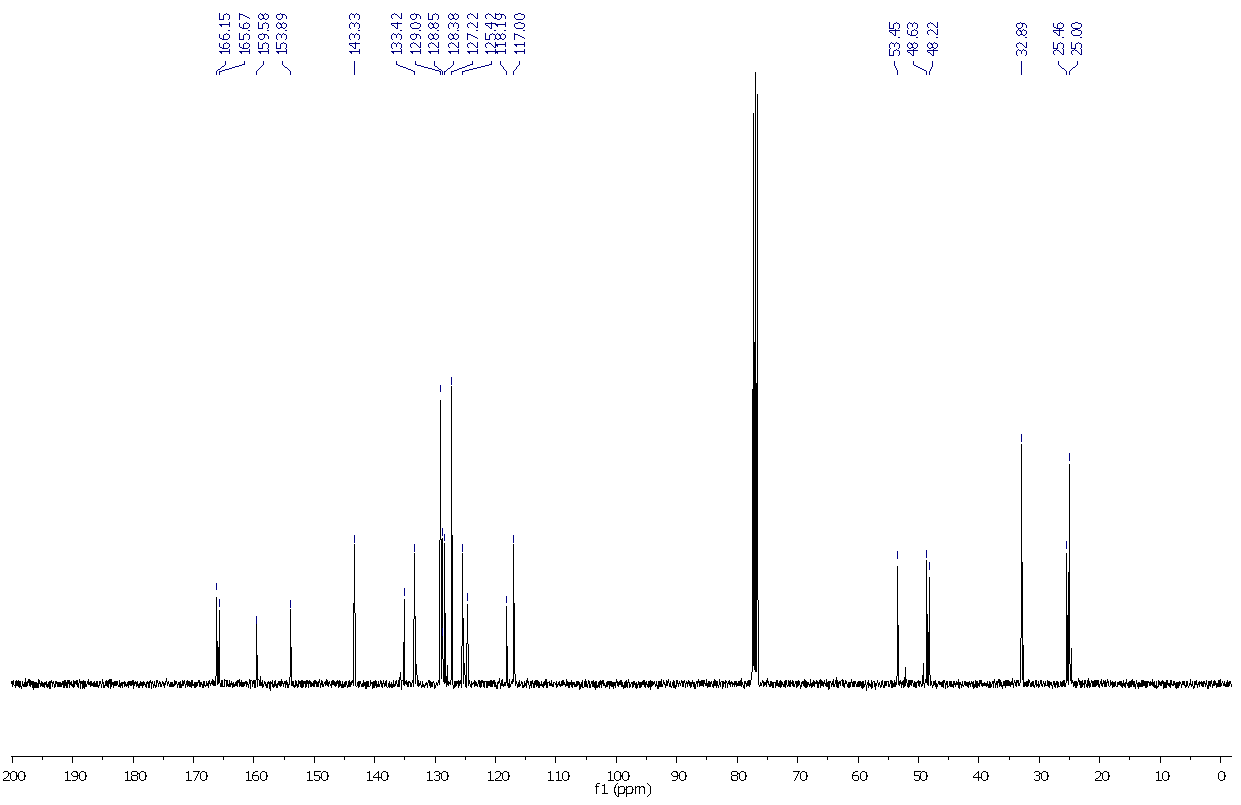


**Figure S2.38.** (Top) 400 MHz ^1^H NMR and (bottom) 100 MHz ^13^C NMR spectra in CDCl_3_ of **U5**.


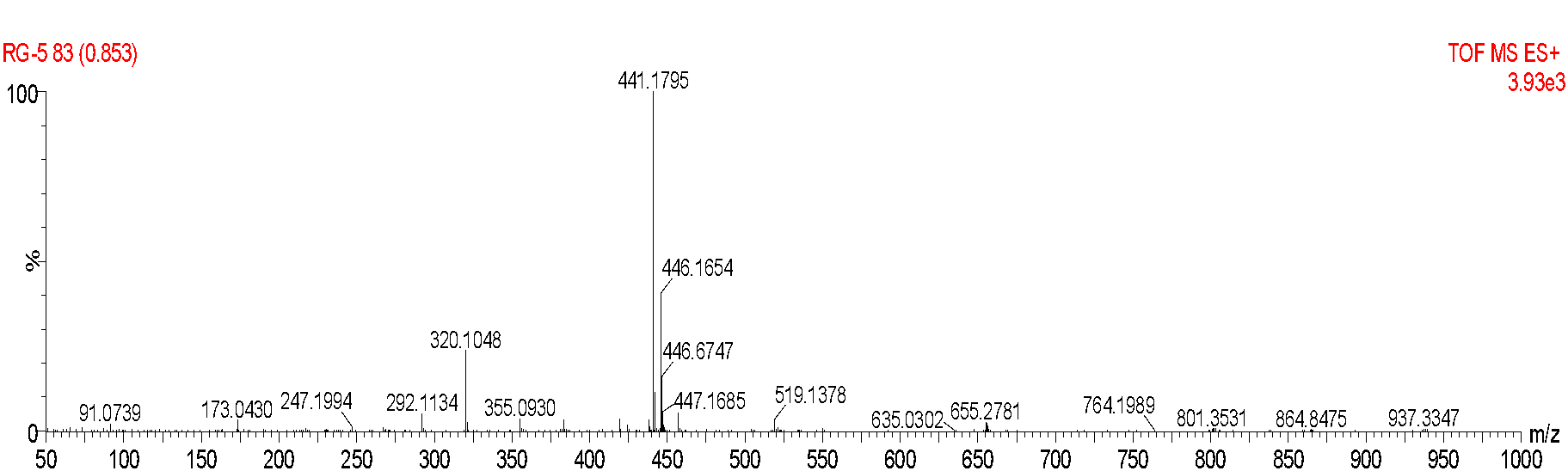


**Figure S2.39.** HRMS spectra of **U5**.


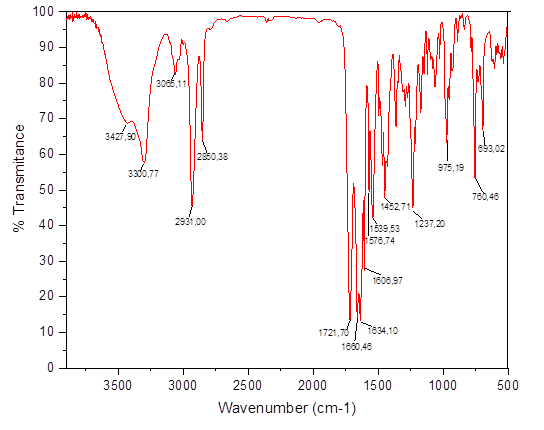


**Figure S2.40.** FT-IR spectra of **U5**.


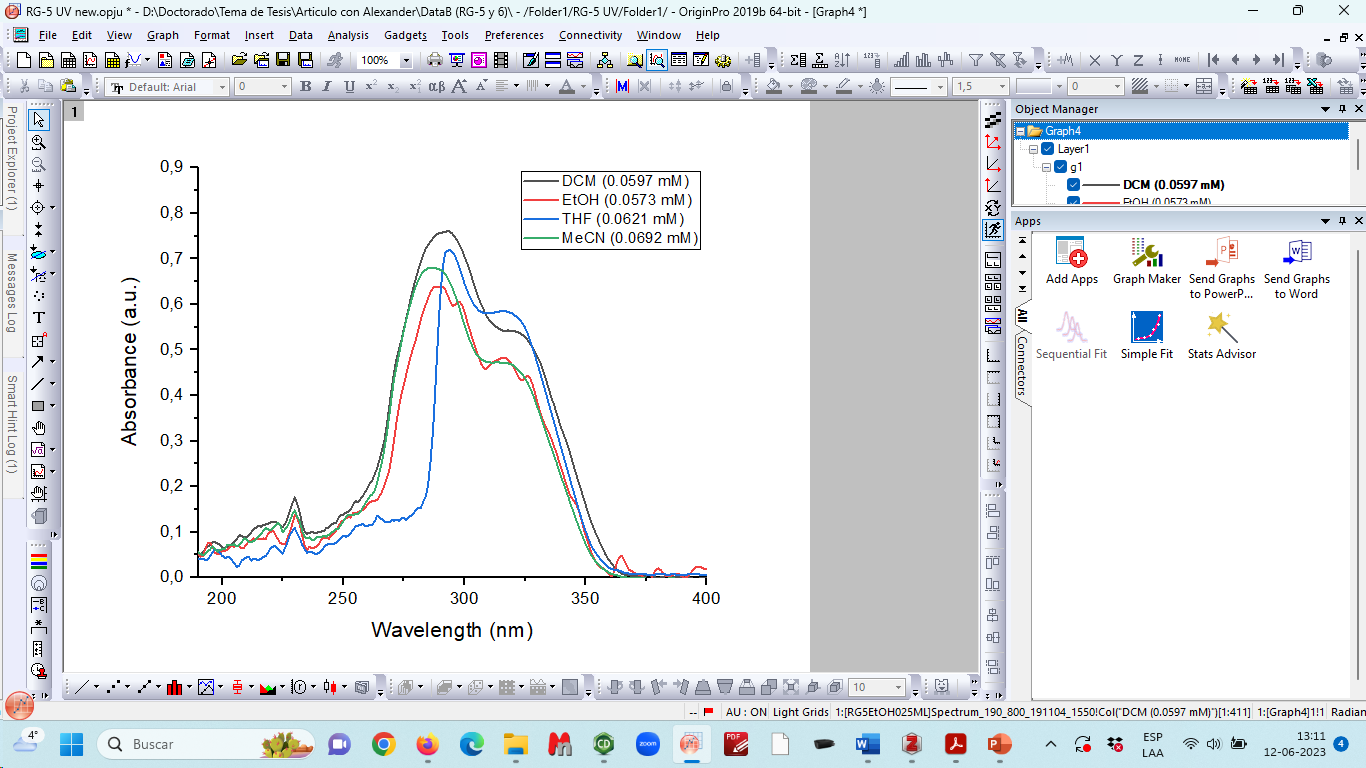


**Figure S2.41.** UV spectra and solvents comparison of **U5**.

^
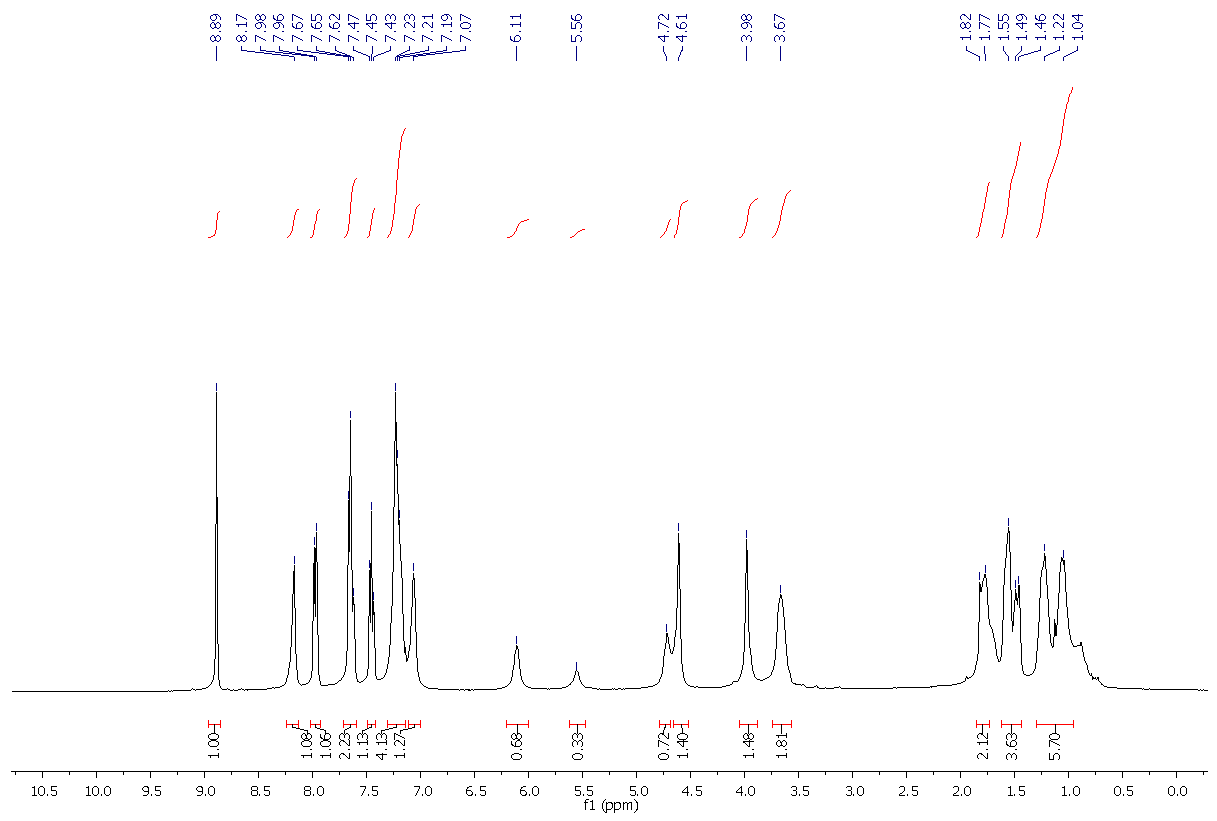
^


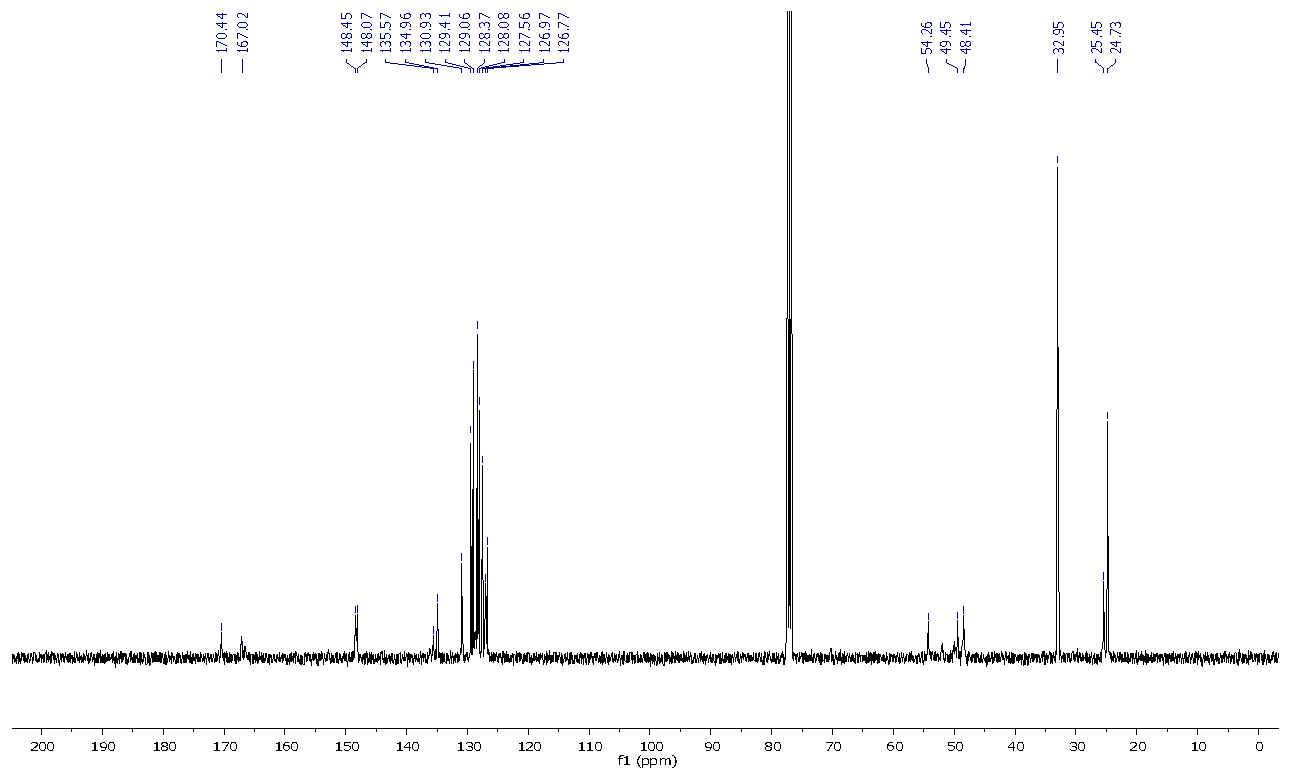


**Figure S2.42.** (Top) 400 MHz ^1^H NMR and (bottom) 100 MHz ^13^C NMR spectra in CDCl_3_ of **U6**.


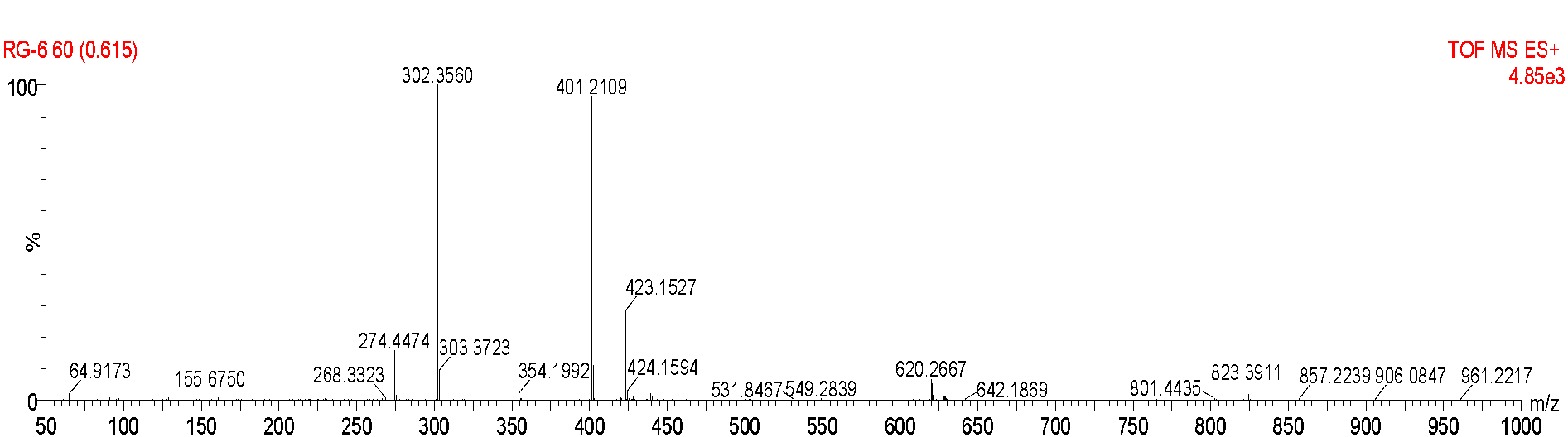


**Figure S2.43.** HRMS spectra of **U6**.


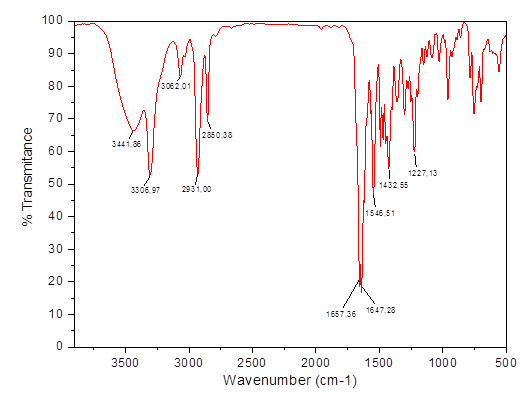


**Figure S2.44.** FT-IR spectra of **U6**.


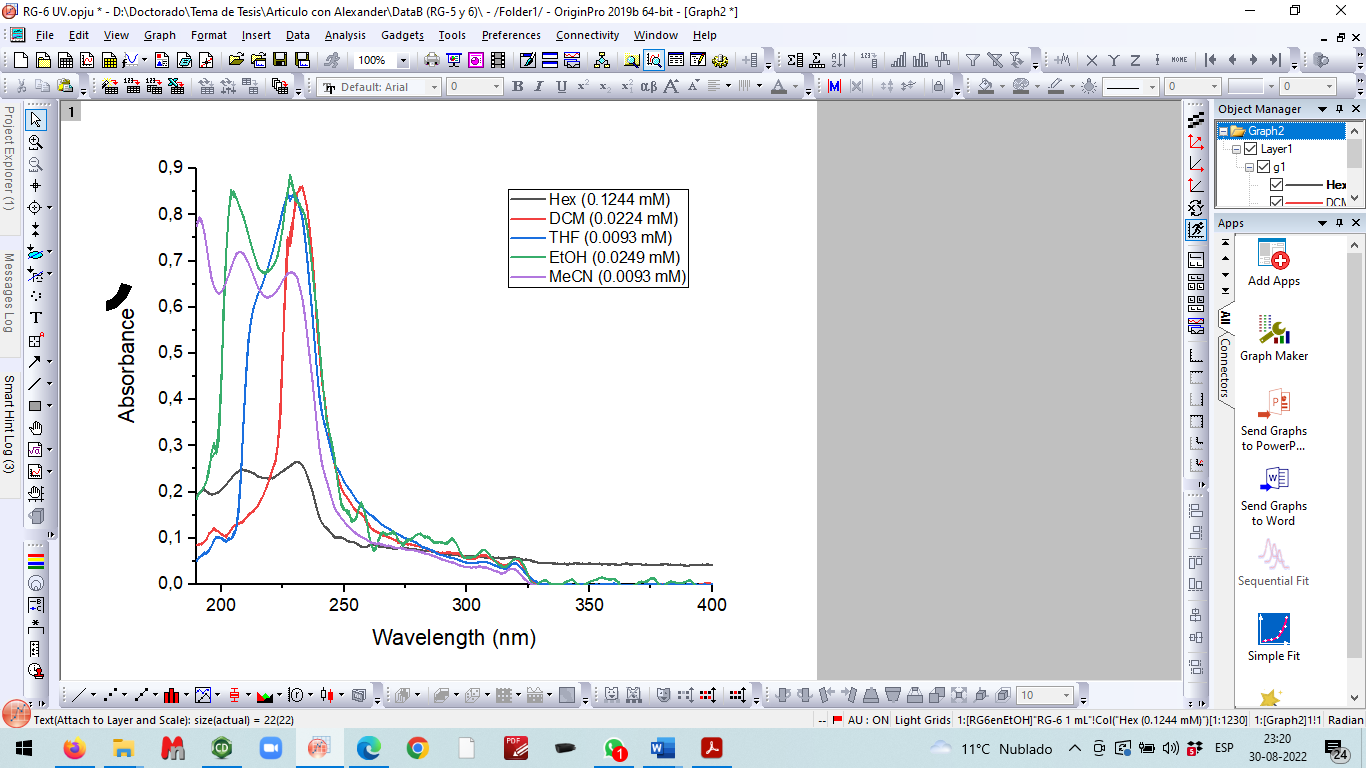


**Figure S2.45.** UV spectra and solvents comparison of **U6**.

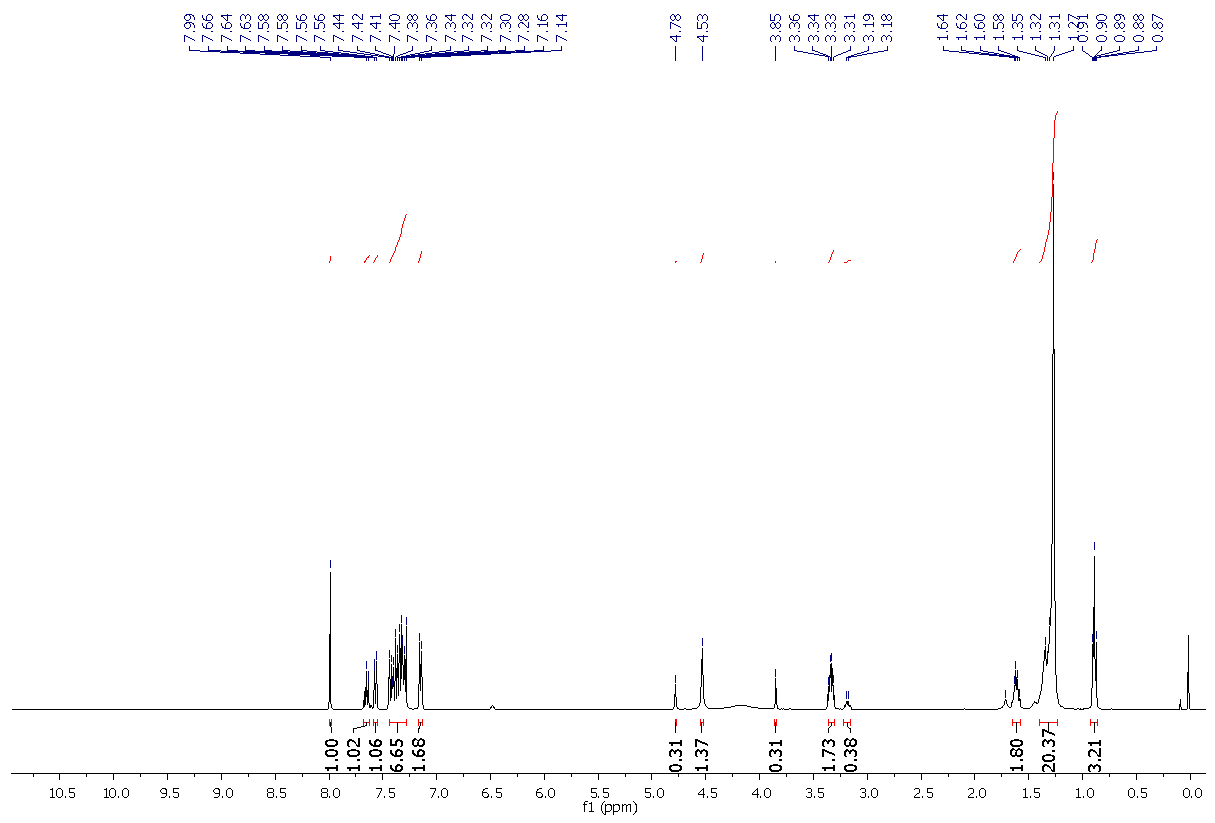


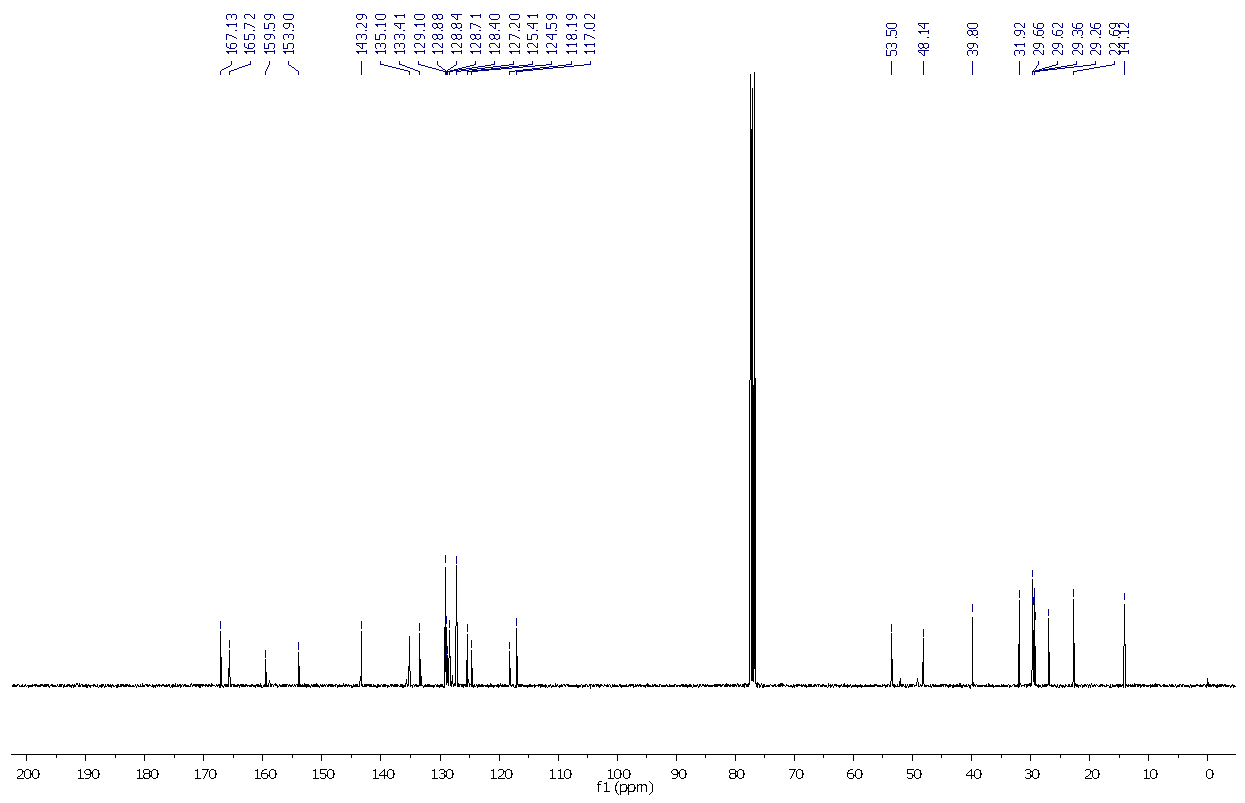


**Figure S2.46.** (Top) 400 MHz ^1^H NMR and (bottom) 100 MHz ^13^C NMR spectra in CDCl_3_ of **U7**.


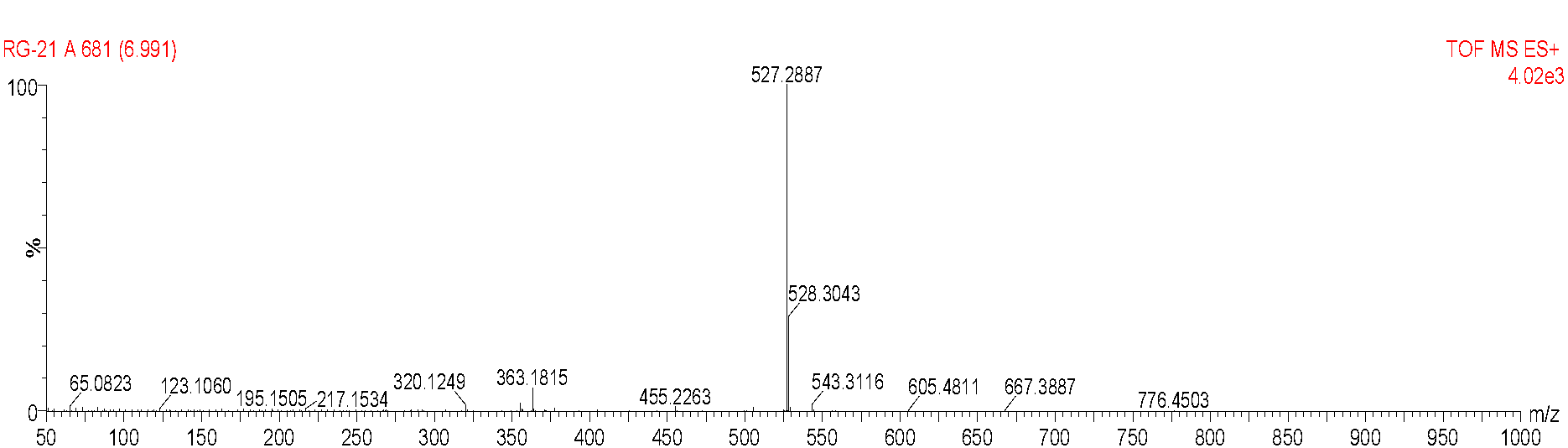


**Figure S2.47.** HRMS spectra of **U7**.


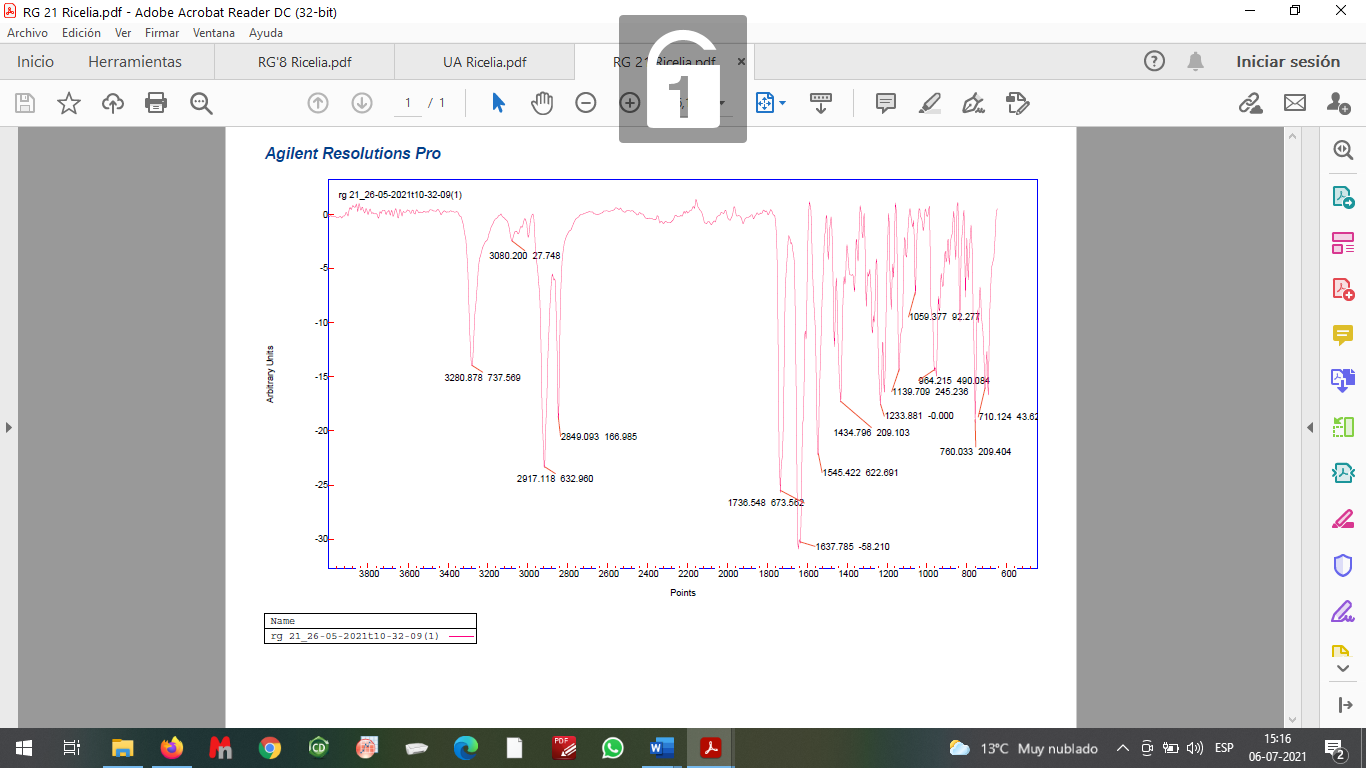


**Figure S2.48.** FT-IR spectra of **U7**.


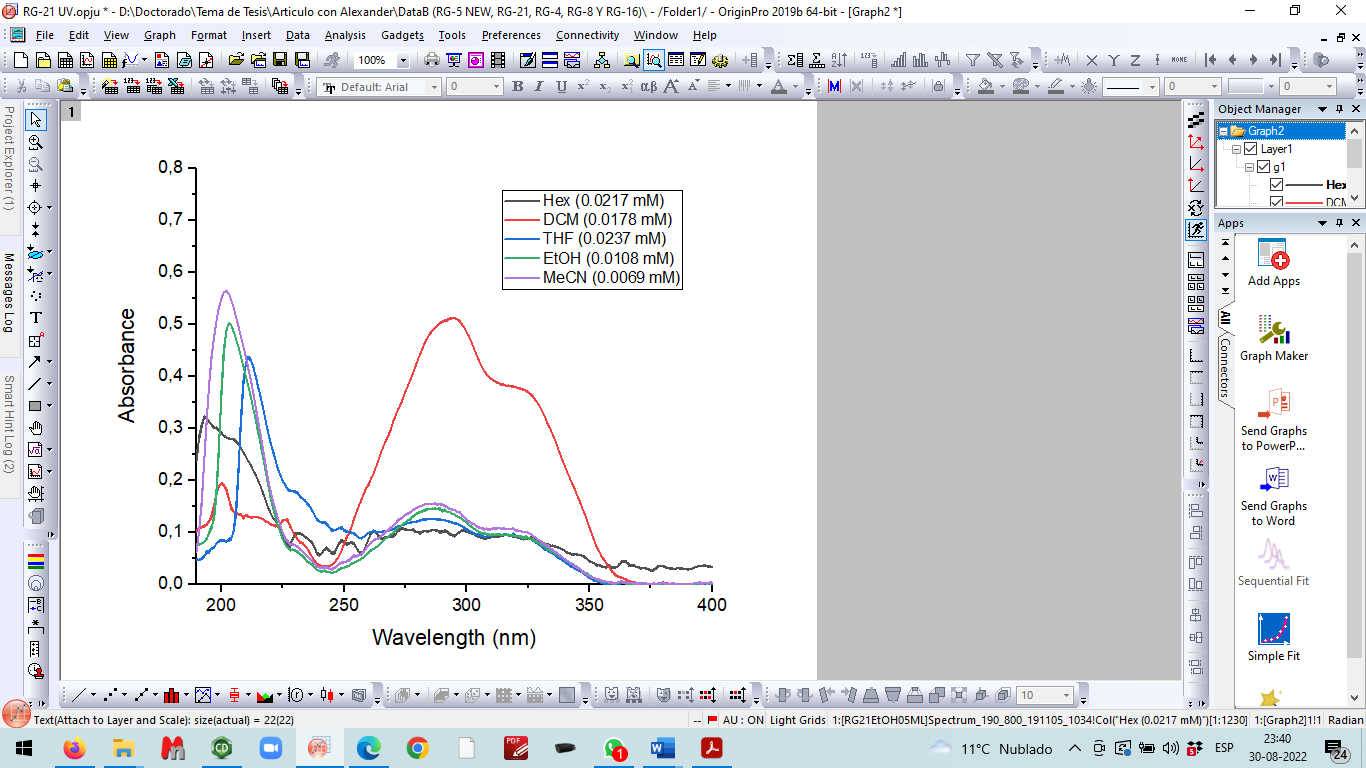


**Figure S2.49.** UV spectra and solvent comparison of **U7**.

a)
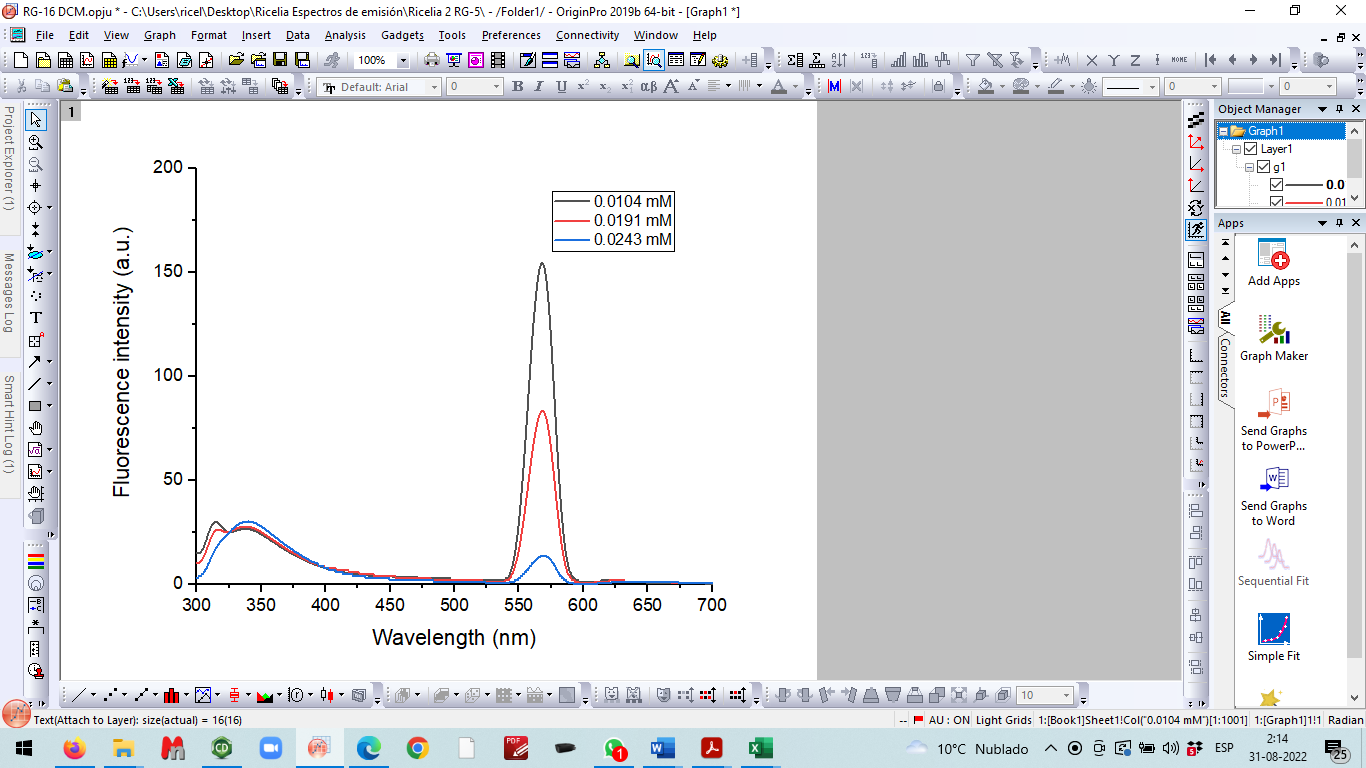
b)
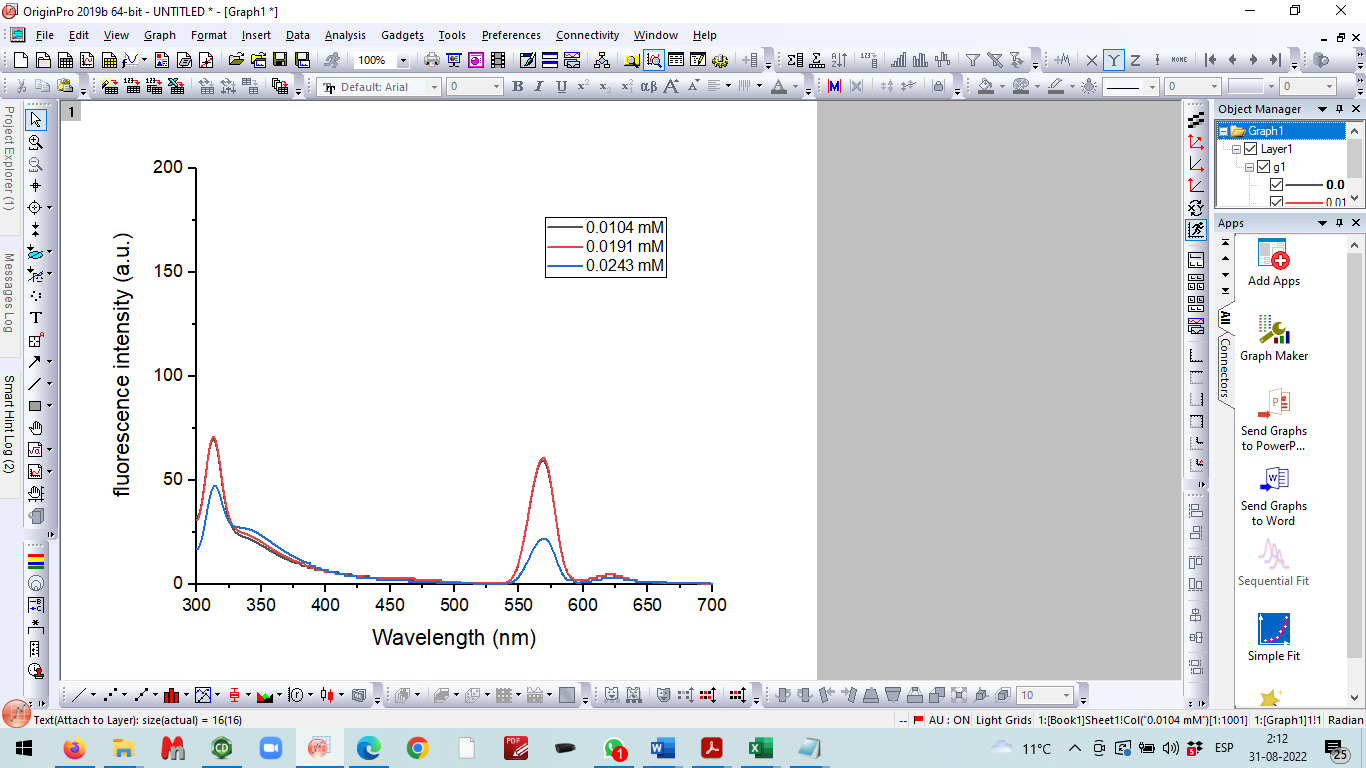


**Figure S2.50.** PL spectra of **P2** at different concentrations a) in DCM, b) in EtOH

a)
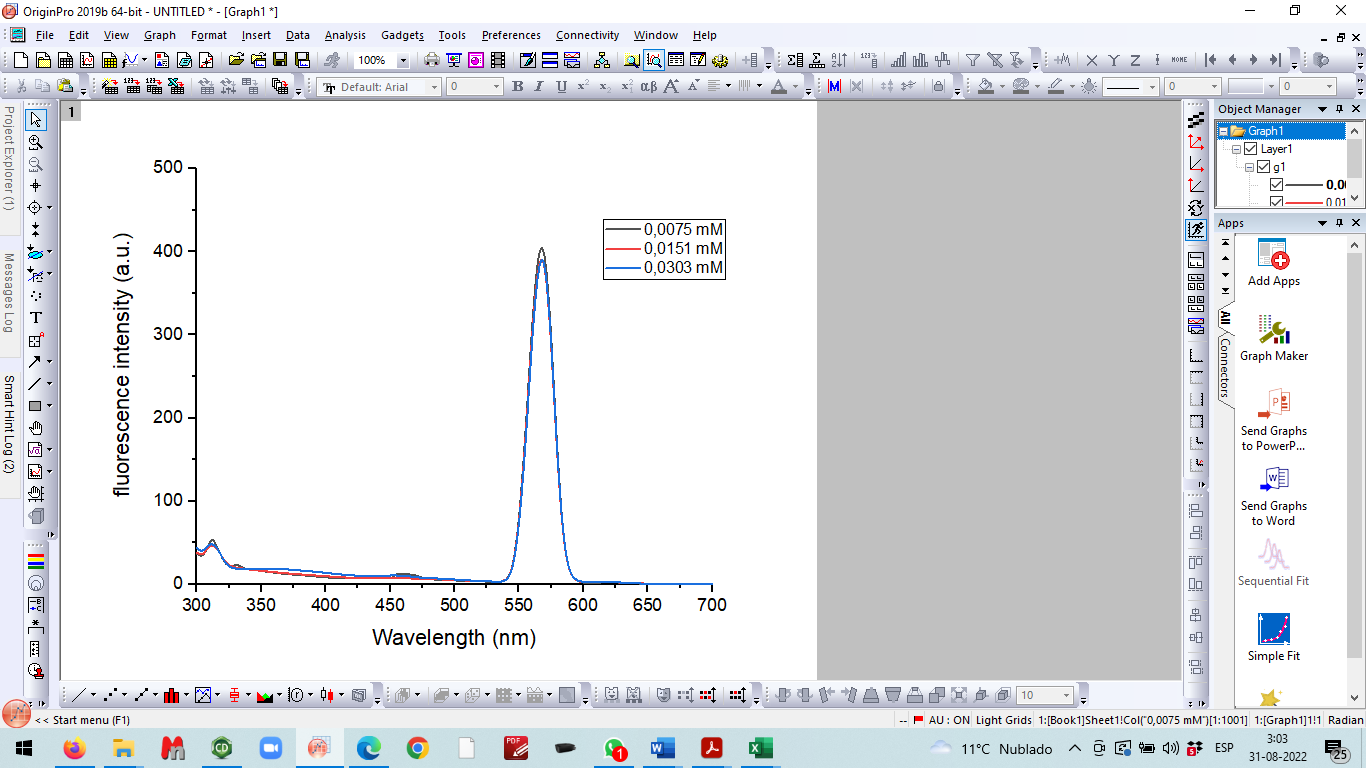
b)
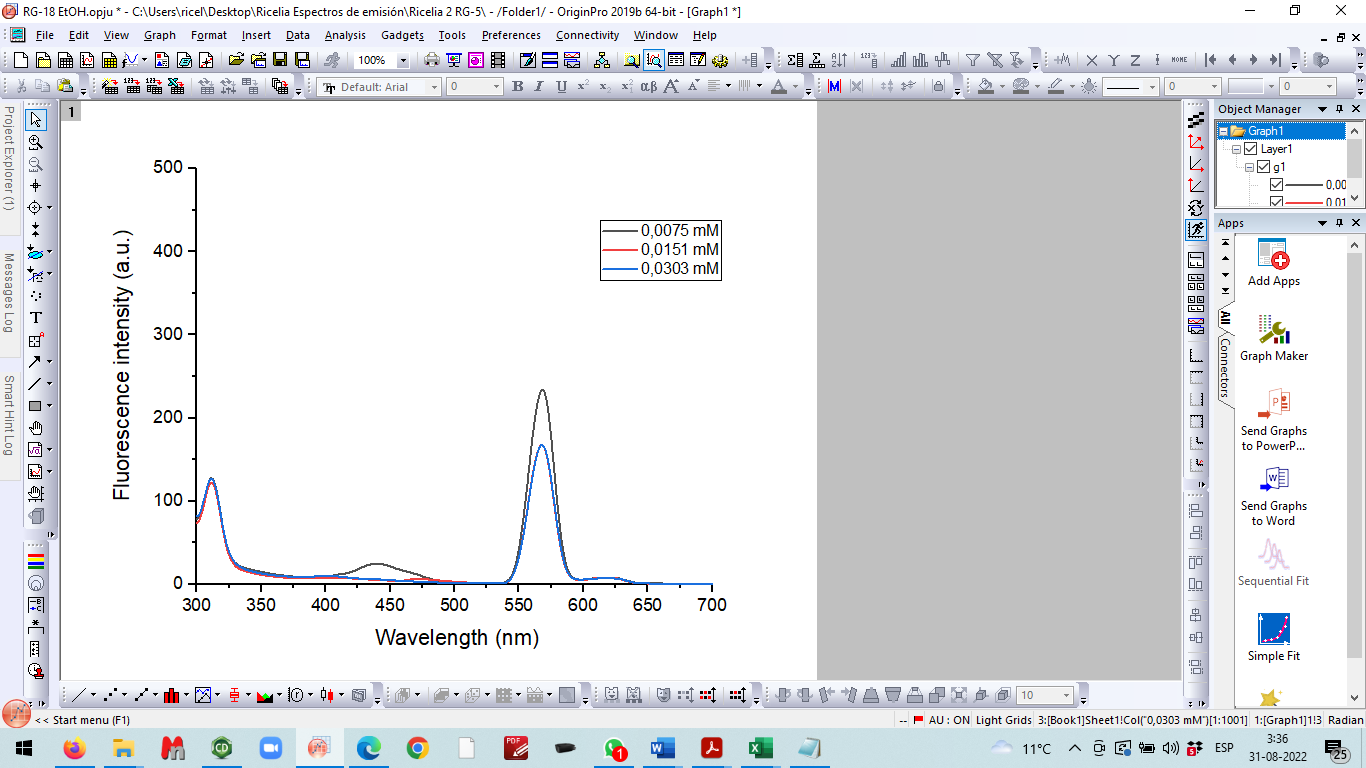
 **Figure S2.51.** PL spectra of **P5** at different concentrations a) in DCM, b) in EtOH

1.
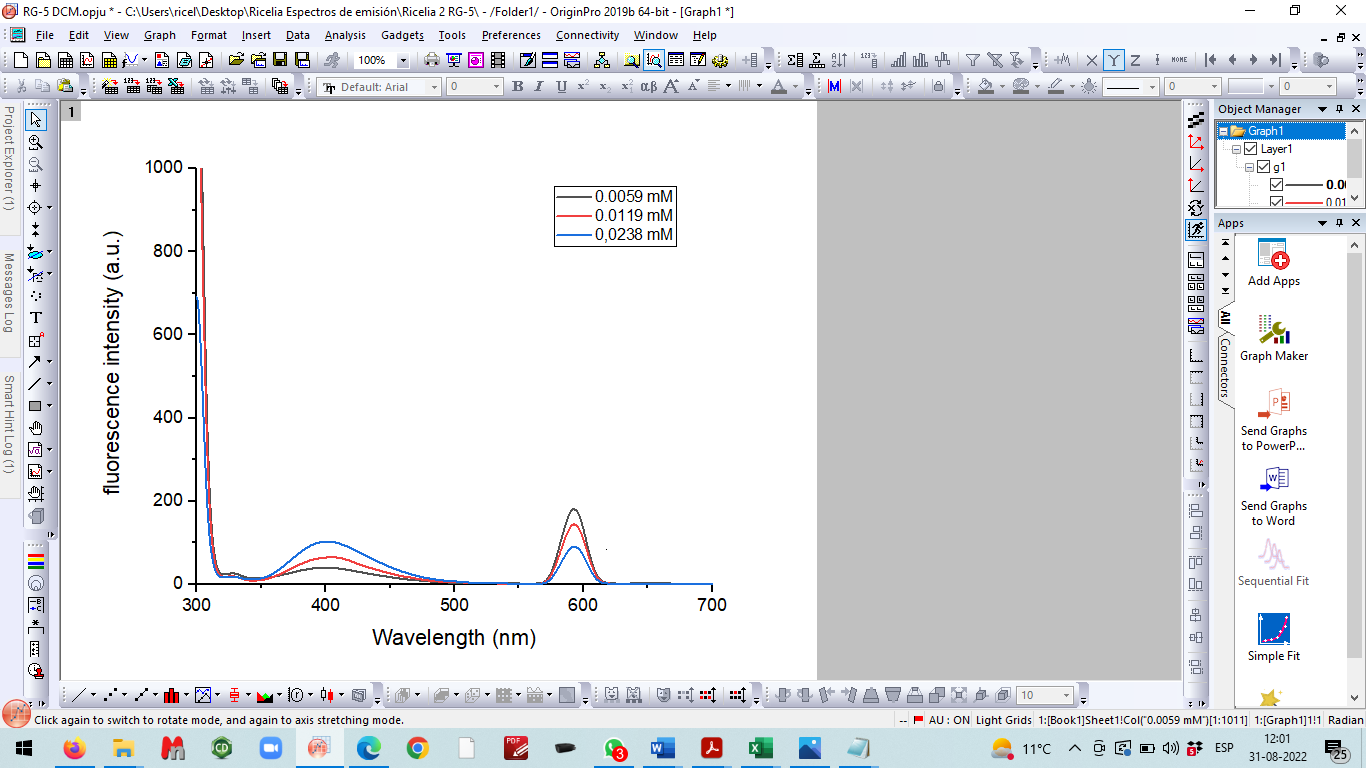
b)
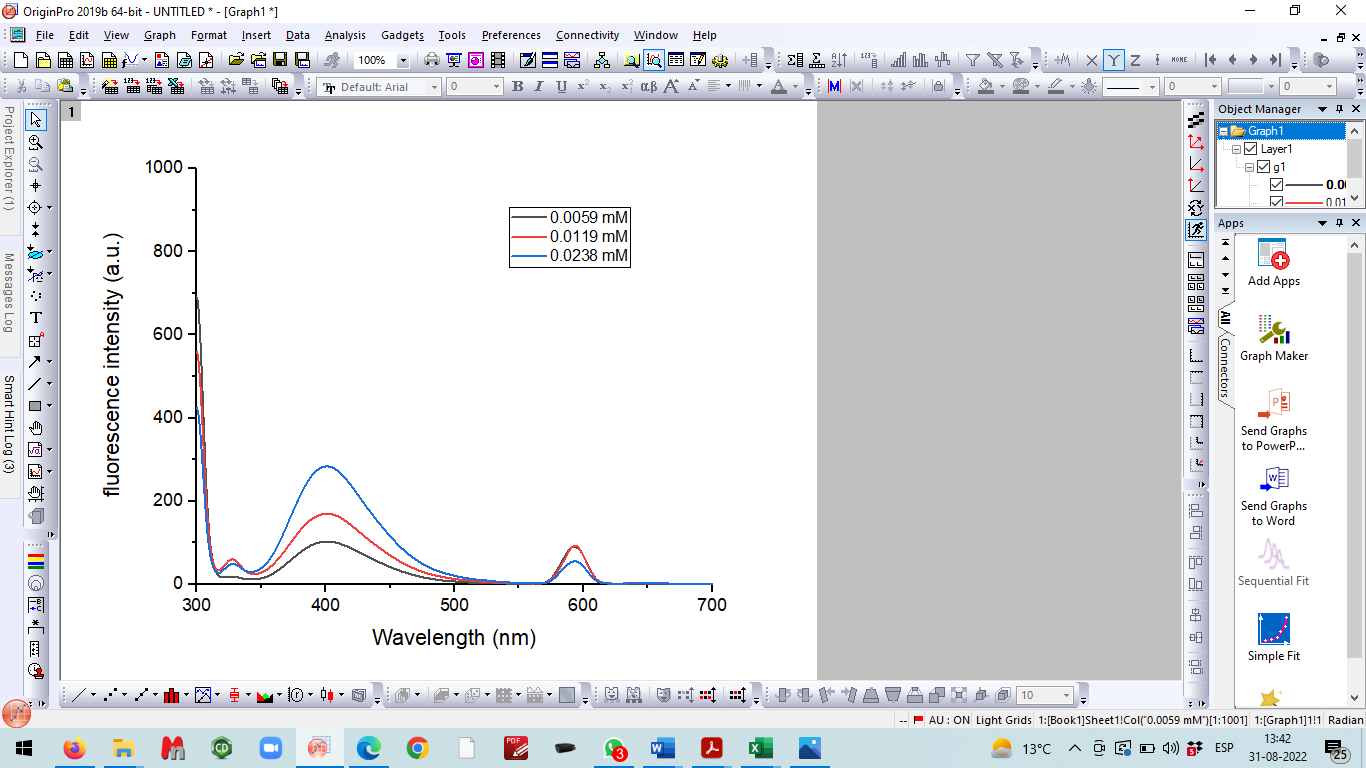


**Figure S2.52.** PL spectra of **U5** at different concentrations a) in DCM, b) in EtOH

**S3.** Fluorescence and Photostability test (Spectrophotometric and HPLC results).


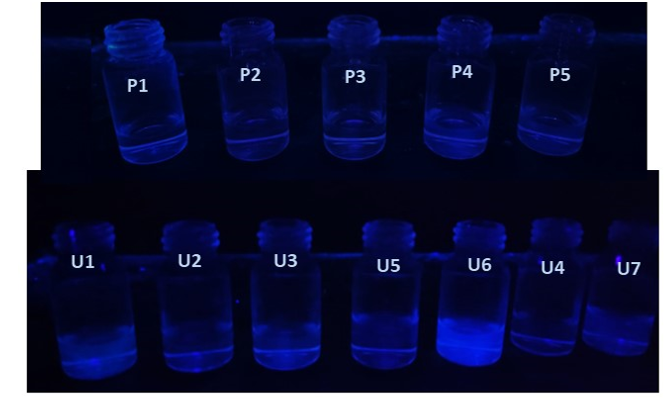


**Figure S3.1.** Fluorescence of compounds under UV light at 365 nm, compounds were dissolved in EtOH. (Only **U6** has fluorescence after excitation by exposure to a UV lamp).

Photostability test: 25 mg of **U5** were placed in a transparent container, without a lid and exposed in a chamber to a UV lamp, with controlled radiation at 254 and 365 nm, trying to comply with the protocols established for photostability studies described in the ICH HARMONIZED TRIPARTITE GUIDELINE (Q1B), at a controlled temperature. The UV spectra, just after preparation (*t* = 0) and at the following times: 3, 5, 10, 20 min to 254 nm and 10, 20 and 30 min at 365 nm, were recorded.

**Table S3.1.** Spectrophotometric data of the behavior of the peptoid **U5** against exposure to UV radiation over time

| **254 nm** | **UV conc (mM)** | **λ _max_** | **Absorbance** |
| --- | --- | --- | --- |
| 3 min | 0.015 | 291 | 0.476 |
| 5 min | 0.025 | 292 | 0.537 |
| 10 min | 0.017 | 291 | 0.545 |
| 20 min | 0.013 | 292 | 0.584 |
| **365 nm** |  |  |  |
| 10 min | 0.023 | 291 | 0.522 |
| 20 min | 0.021 | 291 | 0.541 |
| 30 min | 0.019 | 291 | 0.528 |

.

Samples were prepared in DCM and measured at a concentration of approximately 0.020 mM. When the sample was exposed directly to UV light, there were no alterations in the absorbent properties of the compound and probably so little structural, providing some insight into its stability against radiation.


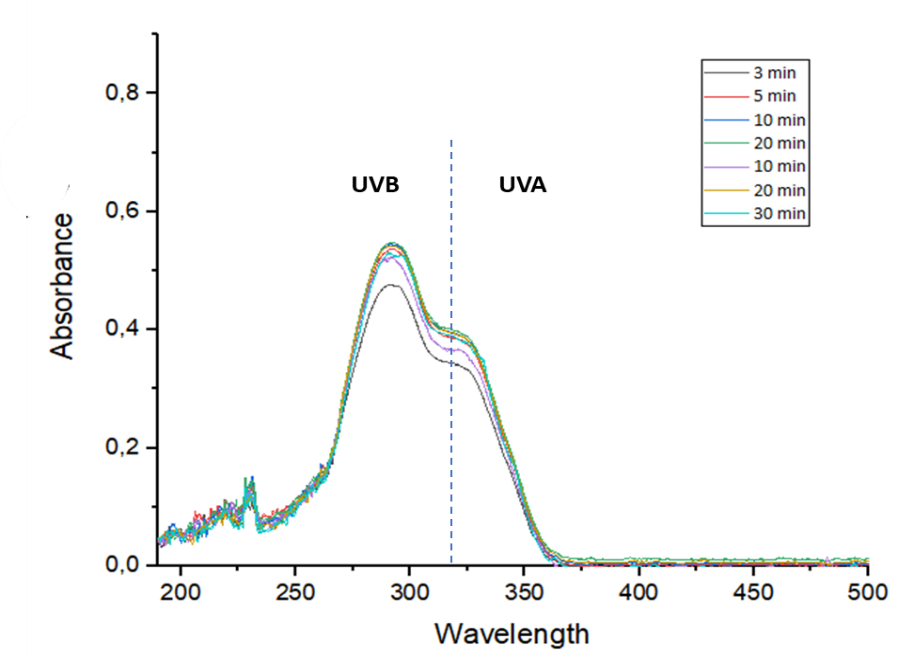


**Figure S3.2.** Behavior of **U5** to UV radiation at 254 and 365 nm over time.

HPLC results: Compounds **U2** and **U5** were measured, based on their stability against UV radiation, using HPLC. 1 mg of sample was dissolved in MeCN, the experiment was carried out using a mixture of solvents, solution A: 0.01% TFA and solution B: 0.01% TFA/MeCN 20/80. C-18 column, injection volume 30 microliters, flow rate 1 mL/min, detection at 254 nm. A chromatogram was taken prior to radiation exposure. And then after 1 h and 3 h of exposure, for both compounds.


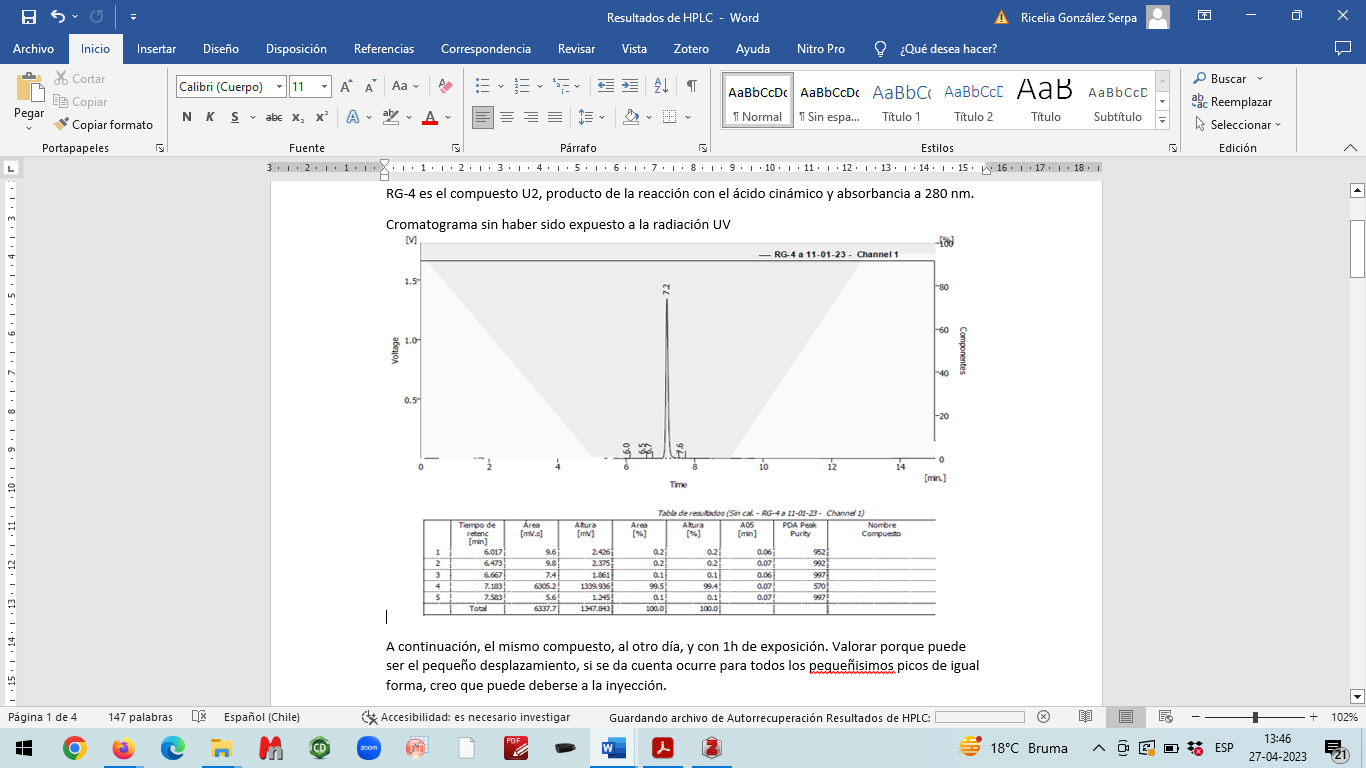
(1)


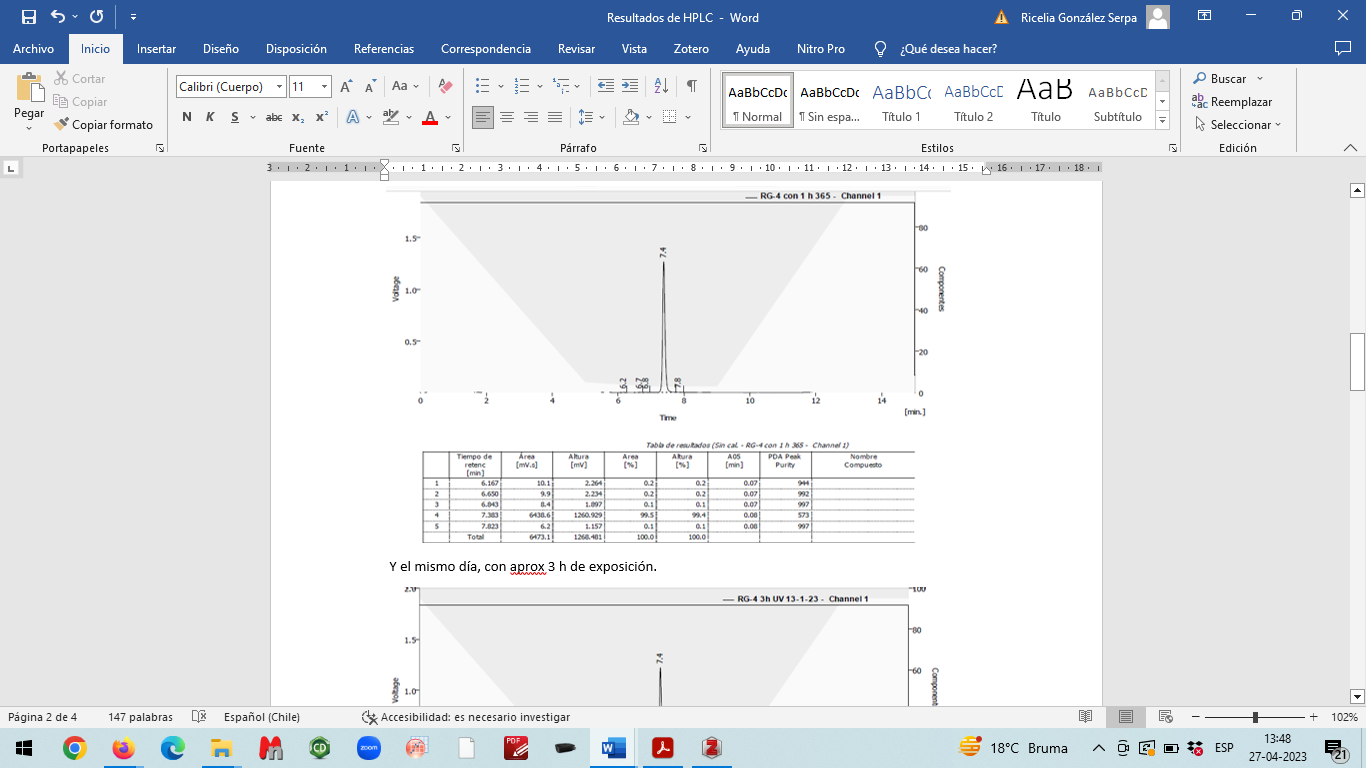
(2)


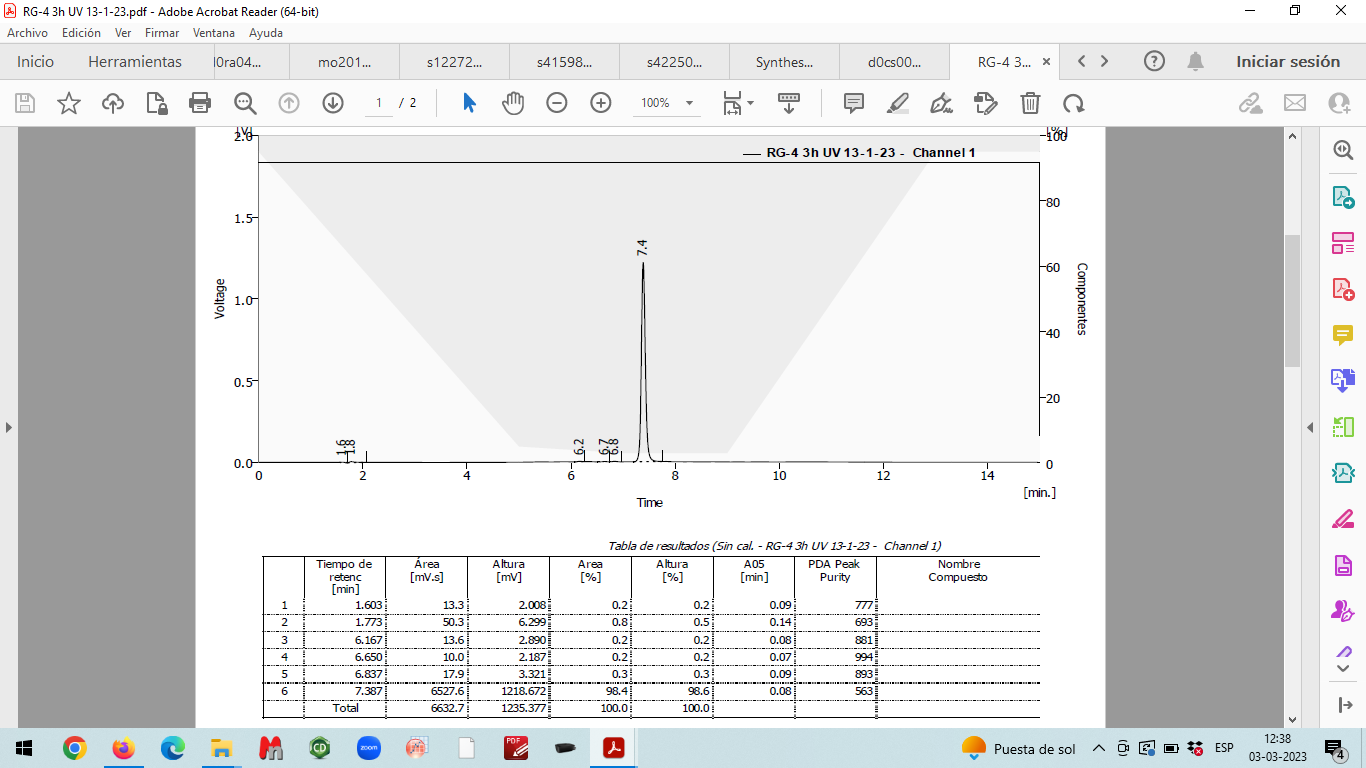
(3)

**Figure S3.3.** Chromatograms of **U2** Prior to radiation exposure (1) after 1 h (2) and 3 h (3) of exposure at 356 nm.


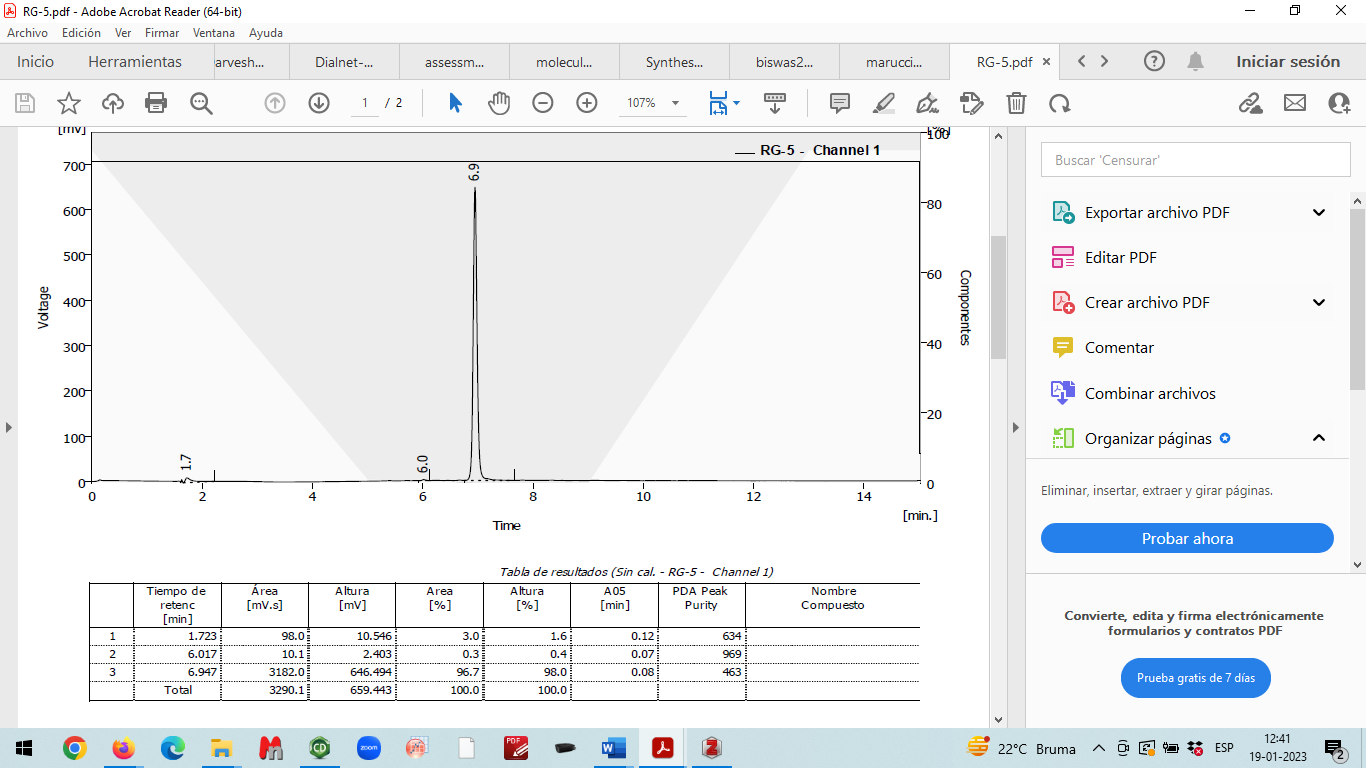
(1)


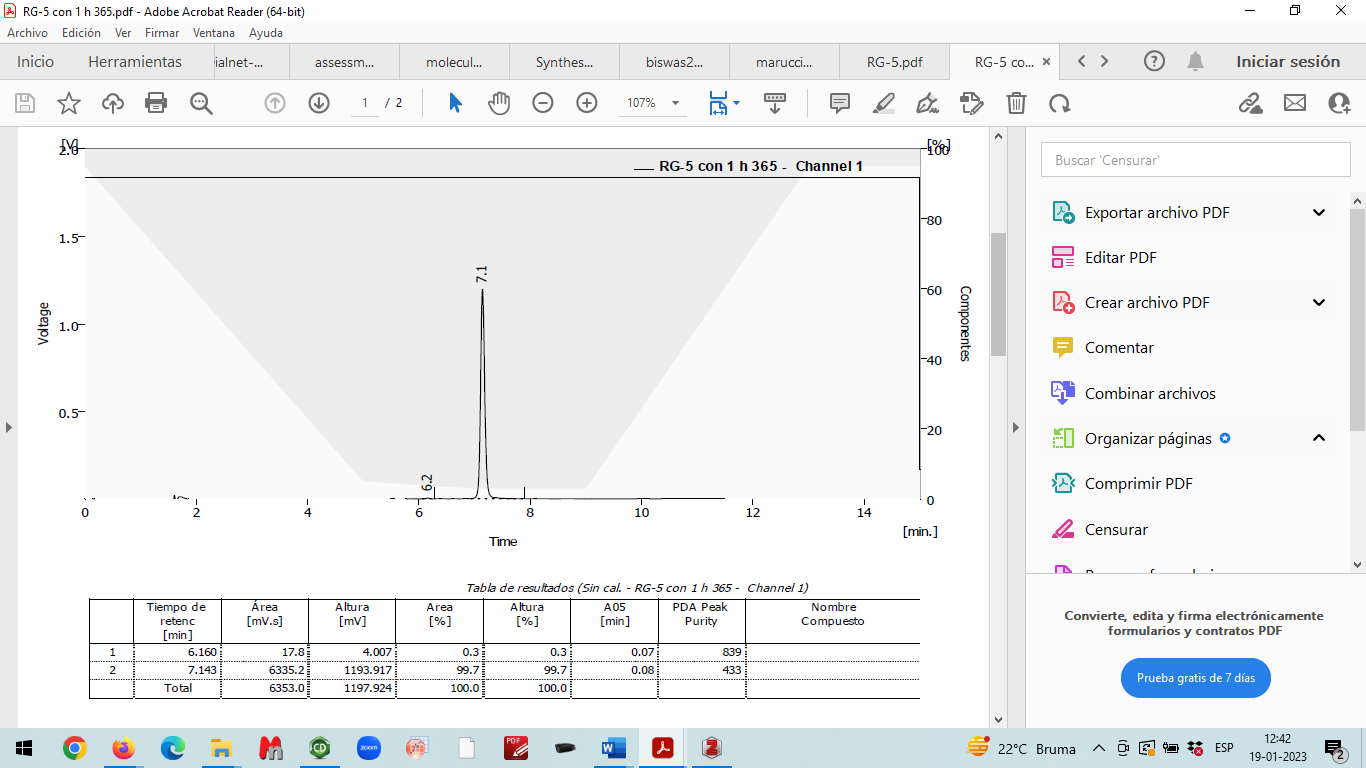
(2)


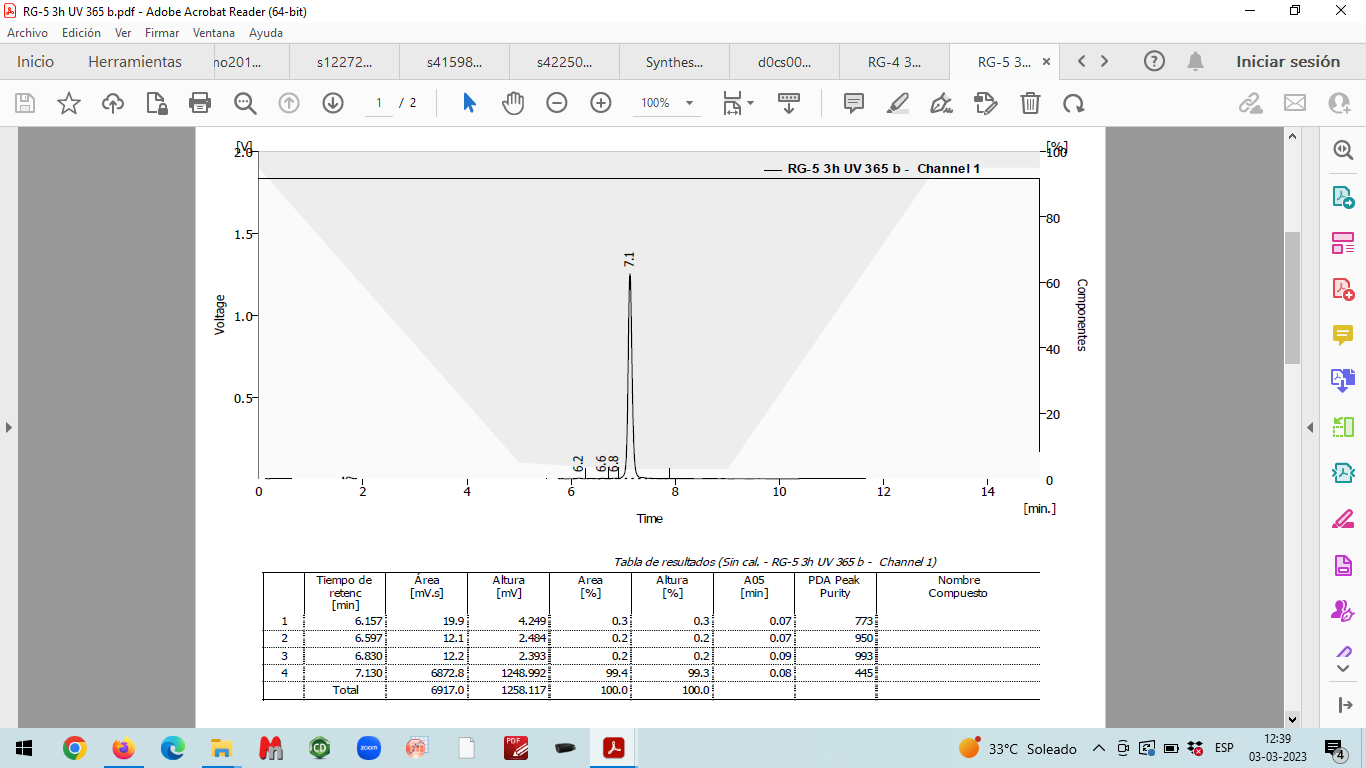
(3)

**Figure S3.4.** Chromatograms of **U5** Prior to radiation exposure (1) after 1 h (2) and 3 h (3) of exposure at 356 nm.
